# A survey of green plant tRNA 3'-end processing enzyme tRNase Zs, homologs of the candidate prostate cancer susceptibility protein ELAC2

**DOI:** 10.1186/1471-2148-11-219

**Published:** 2011-07-23

**Authors:** Lijuan Fan, Zhikang Wang, Jinyu Liu, Weili Guo, Jie Yan, Ying Huang

**Affiliations:** 1 Laboratory of Yeast Genetics and Molecular Biology, School of Life Sciences, Nanjing Normal University, 1 Wenyuan Road, Nanjing 210046, China; 2 Jiangsu Key Laboratory for Microbes and Genomics, School of Life Sciences, Nanjing Normal University, 1 Wenyuan Road, Nanjing 210046, China; 3 Jiangsu Engineering and Technology Research Center for Industrialization of Microbial Resources, School of Life Sciences, Nanjing Normal University, 1 Wenyuan Road, Nanjing 210046, China; 4 Jiangsu Key Laboratory for Biodiversity and Biotechnology, School of Life Sciences, Nanjing Normal University, 1 Wenyuan Road, Nanjing 210046, China

**Keywords:** tRNA precursor (pre-tRNA), endonuclease, tRNase Z, ELAC2, post-transcriptional processing, green plant, phylogenetics

## Abstract

**Background:**

tRNase Z removes the 3'-trailer sequences from precursor tRNAs, which is an essential step preceding the addition of the CCA sequence. tRNase Z exists in the short (tRNase Z^S^) and long (tRNase Z^L^) forms. Based on the sequence characteristics, they can be divided into two major types: bacterial-type tRNase Z^S ^and eukaryotic-type tRNase Z^L^, and one minor type, *Thermotoga maritima *(TM)-type tRNase Z^S^. The number of tRNase Zs is highly variable, with the largest number being identified experimentally in the flowering plant *Arabidopsis thaliana*. It is unknown whether multiple tRNase Zs found in *A. thaliana *is common to the plant kingdom. Also unknown is the extent of sequence and structural conservation among tRNase Zs from the plant kingdom.

**Results:**

We report the identification and analysis of candidate tRNase Zs in 27 fully sequenced genomes of green plants, the great majority of which are flowering plants. It appears that green plants contain multiple distinct tRNase Zs predicted to reside in different subcellular compartments. Furthermore, while the bacterial-type tRNase Z^S^s are present only in basal land plants and green algae, the TM-type tRNase Z^S^s are widespread in green plants. The protein sequences of the TM-type tRNase Z^S^s identified in green plants are similar to those of the bacterial-type tRNase Z^S^s but have distinct features, including the TM-type flexible arm, the variant catalytic HEAT and HST motifs, and a lack of the PxKxRN motif involved in CCA anti-determination (inhibition of tRNase Z activity by CCA), which prevents tRNase Z cleavage of mature tRNAs. Examination of flowering plant chloroplast tRNA genes reveals that many of these genes encode partial CCA sequences. Based on our results and previous studies, we predict that the plant TM-type tRNase Z^S^s may not recognize the CCA sequence as an anti-determinant.

**Conclusions:**

Our findings substantially expand the current repertoire of the TM-type tRNase Z^S^s and hint at the possibility that these proteins may have been selected for their ability to process chloroplast pre-tRNAs with whole or partial CCA sequences. Our results also support the coevolution of tRNase Zs and tRNA 3'-trailer sequences in plants.

## Background

tRNA 3'-end maturation is a process through which the 3'-trailer sequence of precursor tRNAs (pre-tRNAs) is removed, and processed tRNAs acquire the CCA end which is absolutely essential for tRNA aminoacylation and protein synthesis (for reviews, see [1-3]). In prokaryotes, this process can be either exonucleolytic or endonucleolytic depending on whether the 3'-CCA sequence is genomically encoded. CCA-containing pre-tRNAs are generally processed by the exonucleases that tend to stop removing nucleotides from the 3'-end upon encountering the transcriptionally encoded CCA, whereas CCA-less pre-tRNAs are processed by a 3'-endonuclease termed tRNase Z (also termed RNase Z or 3'-tRNase; for reviews, see [4-7]) that cleaves immediately after the N^73 ^discriminator nucleotide (the first unpaired base after the acceptor stem) to allow subsequent addition of the CCA sequence.

Unlike prokaryotic pre-tRNAs, eukaryotic nuclear and organellar pre-tRNAs generally lack the 3'-CCA sequence (which is added post-transcriptionally) and their 3'-trailer sequences are removed by tRNase Z. Also unlike prokaryotic pre-tRNAs, eukaryotic nuclear pre-tRNAs contain oligo (U) at their 3'-ends, which are recognized and bound by the La protein (for reviews see [2,8]). In the budding yeast *Saccharomyces cerevisiae *and fission yeast *Schizosaccharomyces pombe*, the endonucleolytic cleavage of nuclear pre-tRNAs requires the presence of the yeast La protein [9,10]. In the absence of the yeast La protein, the 3'-trailer sequence of nuclear pre-tRNAs is trimmed by 3'-exoribonucleases including Rex1p [11]. However, organellar pre-tRNAs lack terminal oligo (U). Furthermore, unlike nuclear pre-tRNAs which are typically monocistronic, most organellar pre-tRNAs are polycistronic [12,13].

tRNase Z is present in all kingdoms of life. It exists in two forms: tRNase Z^S ^[300-400 amino acids (aa)] and tRNase Z^L ^(700-800 aa), which are encoded by different genes. It is believed that the tRNase Z^L ^gene has evolved from a tandem duplication of the tRNase Z^S ^gene, followed by divergence of the sequence [14]. In prokaryotes, only tRNase Z^S ^is identified. By contrast, all eukaryotes possess tRNase Z^L^, and some have both forms.

The species distribution of tRNase Z is complex. The majority of eukaryotic species analyzed to date, including *S. cerevisiae*, the fruit fly *Drosophila melanogaster *and the nematode worm *Caenorhabditis elegans *contain a single tRNase Z^L ^[15-17]. In contrast, *S. pombe *have two tRNase Z^L^s [18,19]. Interestingly, two tRNase Z^L^s and two tRNase Z^S^s have been experimentally identified in the flowering plant *Arabidopsis thaliana *[20]. In humans, one tRNase Z^S ^(also termed ELAC1) and one tRNase Z^L ^(also termed ELAC2) are found [14]. Our BLAST searches against public genomic and expressed sequence tag (EST) databases reveal that with few exceptions, vertebrates contain one tRNase Z^L ^and one tRNase Z^S ^(a detailed description of tRNase Z protein distribution in the animal kingdom will be provided elsewhere).

tRNase Z belongs to the metallo-*β*-lactamase (MBL) superfamily [14,21-24]. The typical MBL domain contains five conserved sequence motifs termed Motifs I-V. Motifs I and IV each harbor an invariant Asp, Motif II (HxHxDH), which is also called the His motif, is the signature motif of the superfamily, whereas Motifs III and V each contain a conserved His residue. Structural studies of tRNase Z^S^s from *E. coli*, *T. maritima *and *B. subtilis *[25-28] and mutation analyses of tRNase Zs from a variety of species [29-35] reveal that the His and Asp residues of Motifs II-V form the active site for coordination of two catalytic zinc ions. In particular, the Asp residue of Motif II may participate in both zinc ion coordination and act as a general base to generate a hydroxide ion for nucleophilic attack on the scissile phosphodiester bond at the cleavage site [25,29]. The Asp residue of Motif I is also catalytically important and appears to stabilize the catalytic site [33].

Besides tRNase Zs, some nucleic acid processing enzymes are also members of the MBL superfamily. Most of these proteins belong to the *β*-CASP (MBL-associated CISF Artemis SNM1/PSO2) subfamily of the MBL [23]. This subfamily includes the 73-kD subunit of the cleavage and polyadenylation specificity factor (CPSF-73) and its yeast homolog Ysh1p, which are involved in endonucleolytic cleavage of pre-mRNA, the Intergrator complex subunit 11 (Int11) involved in the 3'-end formation of small nuclear RNAs (snRNA) [36], bacterial RNase J, which participates in rRNA 5'-end maturation [37] and RNA decay [38], and the eukaryotic Pso/Snm1/Artemis proteins, which function in DNA repair and V(D)J recombination [23]. However, unlike tRNase Zs, *β*-CASP proteins contain conserved *β*-CASP sequence motifs in place of Motif V.

tRNase Z is distinguished from other MBL members by their unique substrate binding domain termed the flexible arm (also termed the exosite). Based on flexible arm type, there are two major types (bacterial- and eukaryotic-types) and one minor type [*T. maritima *(TM)-type] of tRNase Zs [39]. The bacterial-type tRNase Zs, which are present predominantly in bacteria, possess the bacterial-type flexible arm. The bacterial-type flexible arm is ~55 aa in length and contains the Gly- and Pro-rich GP motif (GxPxGP, sometimes GxPPGP) [39]. The eukaryotic-type tRNase Zs, which are found only in eukaryotes, contain the ELAC2-type flexible arm. This type of flexible arm harbors the GP motif and is ~62 aa long, which is slightly longer than the bacterial-type flexible arm.

The TM-type tRNase Z was believed to be the minor type at the time of discovery since it was found only in *T. maritima *and *A. thaliana *[39]. The flexible arm found in TM-type tRNase Zs appears to be shorter (~30 aa) and lacks the GP motif but instead contains one short basic residue-rich region [39]. In addition, both the bacterial- and eukaryotic-type tRNase Zs contain the PxKxRN, HEAT and HST motifs, which form part of loop structures, whereas the TM-type tRNase Z lacks these motifs [33,40,41]. The PxKxRN motif has been suggested to function in CCA anti-determination (tRNase Z activity is inhibited by 3'-CCA) [25,33], whereas the HEAT and HST motifs have been suggested to play a role in facilitating proton transfer at the final stage of reaction [25,29,40].

tRNase Z has diverse functions besides its primary role in tRNA 3'-end processing. This is perhaps best exemplified by ELAC2, which serves a multitude of functions within cells. Recent studies have shown that ELAC2 is involved in the generation of MALAT1, a cancer-associated long noncoding RNA which participates in regulation of pre-mRNA splicing [42], tRNA-derived small RNAs [43,44], and viral microRNAs (miRNAs) [45,46]. Overexpression of *ELAC2 *delays cell cycle progression, suggesting that ELAC2 may be involved in cell cycle control either directly or indirectly via its role as tRNA processing enzyme [47]. ELAC2 also potentiates TGF-*β*(transforming growth factor-*β*/Smad-induced transcription response, indicating a role for ELAC2 in TGF-*β*/Smad signaling mediated growth arrest [48]. Interestingly, a recent study has shown that destruction of human mitochondria through depletion of mitochondrial DNA results in down-regulation of *ELAC2 *and a delay in cell cycle progression [49]. Since ELAC2 may be involved in cell cycle regulation, it is likely that ELAC2 may link mitochondrial function and cell cycle control. It is important to note that *ELAC2 *is a candidate prostate cancer susceptibility gene as its mutations are associated with prostate cancer [14]. However, the underlying mechanisms are unknown. In *S. cerevisiae*, either inactivating mutations or overexpression of tRNase Z^L ^causes a petite phenotype, suggesting that the action of tRNase Z^L ^may be related to mitochondrial function [15]. In addition, the *S. cerevisiae *tRNase Z^L ^has also been suggested to play a role in 35S rRNA processing [50].

The study of tRNase Z evolution has been facilitated by the increasing availability of genome sequences. A previous study showed that only tRNase Z^S ^is found in bacteria and that its presence in bacteria is widespread [6]. We recently reported on a systematic survey of tRNase Zs in fungi [51]. Our analysis reveals that while the majority of fungal species contain one tRNase Z^L^, all four sequenced *Schizosaccharomyces *species contain two distinct tRNase Z^L^s either demonstrated or predicted to be localized to the nucleus and mitochondria, respectively. In addition, the presence of tRNase Z^S ^in fungi is restricted to the phylum Basidiomycota and the basal fungal phyla.

Green plants (Viridiplantae) represent a monophyletic group of land plants and green algae that evolved near the base of the tree of eukaryotic life. Flowering plants (angiosperms), which are typically polyploidy, represent the largest, most diverse and most evolutionary advanced phylum of land plants making up 90% of the plant kingdom. It can be divided into two major groups: dicotyledons (dicots), which accounts for the majority of the angiosperm species, and monocotyledons (moncots). At present, there are at least 27 sequenced and annotated genomes representing the major taxonomic groups within green plants, although the majority of them are those of flowering plants. The public availability of these genome sequences enabled us to identify tRNase Zs in green plants and to study their evolution.

In this study, we undertook a comprehensive survey of candidate tRNase Zs from annotated green plant genomes. To understand the evolutionary relationships among green plant tRNase Zs, we further conducted a phylogenetic analysis of these newly identified candidates. Finally, we presented a detailed sequence analysis of tRNase Zs with the intent of further delineating the distinct features of green plant tRNase Zs.

## Results

### Identification of candidate green plant tRNase Zs

To extend our previous study of tRNase Z diversity and evolution, we searched public genome databases for putative green plant tRNase Zs with significant matches to known bacterial and eukaryotic tRNase Zs. Since most of candidate sequences identified from the databases are computationally generated without subsequent manual annotation, it is likely that many predictions may contain errors. Therefore, we verified each candidate. We first validated each prediction by reciprocal searches against the GenBank. In back-searches, a candidate was confirmed if reverse BLAST also gave tRNase Z hits in the top matches. Accuracy of prediction was further evaluated by multiple sequence alignment. All discordant candidate sequences were checked manually for possible errors including sequencing errors, intron mispredictions and existence of gaps in the genome sequences. We found that many candidate sequences are apparently incomplete or contain annotation errors. For example, the predicted coding sequence of the monkeyflower (*Mimulus guttatus*) MguTRZ2 (Phytozome accession no. mgv1a024577 m.g) in the database was incomplete lacking the N-terminal region. We were able to predict this region from the genomic DNA sequence based on sequence similarity. The predicted full-length coding region of MguTRZ2 has 364 aa. As another example, the sequence annotated as the candidate castor bean (*Ricinus communis*) tRNase Z^L ^(Phytozome accession no. 30146.t000117) appears to be mispredicted due to the presence of sequence gaps. Thus, this sequence was excluded from the list.

Several incorrect predictions are apparently caused by the presence of the non-canonic GC-AG splice site pairs. While the GT donor splice site is a canonical 5'-splice site for introns in eukaryotic genes, the GC donor splice sites account for the majority of the non-canonical donor splice site. Thus, for those that cannot be accurately predicted by the conventional FGENESH program, we carried out gene prediction using FGENESH _GC, which is a new version of the FGENESH program including noncanonical GC dinucleotide in donor splice sites. Indeed, by doing so, we could predict some exons encoding missing conserved motifs. For example, the HEAT motif was originally missing in several annotated candidate tRNase Z^L^s from flowering plants including papaya (*Carica papaya *CpaTRZ3), cassava (*Manihot esculenta *MesTRZ4), *Medicago *(*Medicago truncatula *MtrTRZ3) and black cottonwood (*Populus trichocarpa *PtrTRZ3). After re-evaluation of intron splice sites using FGENESH_GC, we were able to recover their HEAT motifs. Because the intron sequences of some candidate sequences appear to be extremely difficult to predict correctly, we could not conclusively rule out the possibility of errors in certain candidates.

In total, 54 candidate tRNase Z^S ^and 32 candidate tRNase Z^L ^were identified from 27 green plant species including 21 flowering plants, 1 moss, 1 lycophyte and 4 green algae (Additional file 1). The names of these candidates follow the *A. thaliana *tRNase Z nomenclature [52]. Of these, only tRNase Zs from *A. thaliana *have been experimentally characterized [20]. While most of these sequenced genomes examined belong to the flowering plants, the availability of the genome sequences from two basal land plants (the bryophyte *Physcomitrella patens *and the lycophyte *Selaginella moellendorffii*) and four green algae (*Chlamydomonas reinhardtii*, *Volvox carteri*, *Micromonas pusilla *and *Ostreococcus lucimarinus*) allows for evaluation of differences between flowering plant tRNase Zs and those from basal land plants and green algae.

The flowering plants examined to date appear to contain multiple tRNase Zs. Foxtail millet (*Setaria italica*) contains the largest number of tRNase Zs (5) so far identified in a flowering plant. Most flowering plant species have a single tRNase Z^L^. In contrast, six flowering plants including two *Arabidopsis *species (*A. thaliana *and *Arabidopsis lyrata*) harbor two tRNase Z^L^s. It should be noted that the presence of two tRNase Z^L^s are not species-specific since the plant species containing two tRNase Z^L^s come from diverse taxonomic groups.

Unlike most flowering plants that possess two tRNase Z^S^s, two members of the *Panicoideae *subfamily of grasses, sorghum (*Sorghum bicolor*) and foxtail millet have three tRNase Z^S^s and four tRNase Z^S^s, respectively. The presence of multiple tRNase Z^S^s appears not to be grass-specific, since three other grass plants including rice (*Oryza sativa*), *Brachypodium *(*Brachypodium distachyon*) and maize (*Zea mays*) contain only two tRNase Z^S^s. Calculation of the percentage identity and similarity between candidate tRNase Zs from these two grass species shows strong conservation of the proteins at the amino acid level with the most identity (93%) and similarity (95%) between *S. bicolor *SbiTRZ2 and *S. italica *SitTRZ2 (Additional files 2 and 3). To our surprise, all candidate tRNase Z^S^s identified in flowering plants are highly similar and belong to the TM-type tRNase Z^S ^(see below for a detailed discussion).

The number and type of tRNase Zs appear to be highly variable in the two primitive plant species and four green algae (Table 1 and Additional file 1). The largest number of tRNase Zs is found in the moss *P. patens*, which contains two TM-type and one bacterial-type tRNase Z^S^s, and one tRNase Z^L^. In contrast, two green algae *C. reinhardtii *and *V. carteria *have the least number of tRNase Zs, comprising one TM-type tRNase Z^S ^and one tRNase Z^L^. The two other green algae *M. pusilla *and *O. lucimarinus *contain one TM-type and one bacterial-type tRNase Z^S^s and one tRNase Z^L^. The lycophyte *S. moellendorffii*, which has the smallest genome size of any land plant reported, contains two tRNase Z^L^s in addition to one TM-type tRNase Z^S^.

**Table 1:** Distribution of candidate tRNase Zs from representative green plants

Species	Protein name	Type	Accession number	Database	No. aa^+^
**DICOTS**					
*Aquilegia coerulea*	AcoTRZ1	TM-type tRNase Z^S^	AcoGoldSmith_v1.019418 m.g	Phytozome	283
*Aquilegia coerulea*	AcoTRZ2	TM-type tRNase Z^S^	AcoGoldSmith_v1.007495 m.g	Phytozome	356
*Aquilegia coerulea*	AcoTRZ3	tRNase Z^L^	AcoGoldSmith_v1.000749 m.g	Phytozome	997*
*Aquilegia coerulea*	AcoTRZ4	tRNase Z^L^	AcoGoldSmith_v1.024294 m.g	Phytozome	848*
*Arabidopsis thaliana*	AthTRZ1	TM-type tRNase Z^S^	NP_177608.2	NCBI	280
*Arabidopsis thaliana*	AthTRZ2	TM-type tRNase Z^S^	NP_178532.2	NCBI	354
*Arabidopsis thaliana*	AthTRZ3	tRNase Z^L^	NP_175628.2	NCBI	890
*Arabidopsis thaliana*	AthTRZ4	tRNase Z^L^	NP_188247.2	NCBI	942
*Carica papaya*	CpaTRZ1	TM-type tRNase Z^S^	evm.TU.supercontig_17.217	Phytozome	306
*Carica papaya*	CpaTRZ2	TM-type tRNase Z^S^	evm.TU.supercontig_132.51	Phytozome	359*
*Carica papaya*	CpaTRZ3	tRNase Z^L^	evm.TU.supercontig_34.31	Phytozome	943*
*Citrus clementina*	CclTRZ1	TM-type tRNase Z^S^	clementine0.9_017657 m.g	Phytozome	288
*Citrus clementina*	CclTRZ2	TM-type tRNase Z^S^	clementine0.9_014182 m.g	Phytozome	355
*Citrus clementina*	CclTRZ3	tRNase Z^L^	clementine0.9_001700 m.g	Phytozome	938
*Cucumis sativus*	CsaTRZ1	TM-type tRNase Z^S^	Cucsa.369470	Phytozome	301*
*Cucumis sativus*	CsaTRZ2	TM-type tRNase Z^S^	Cucsa.185180	Phytozome	347
*Cucumis sativus*	CsaTRZ3	tRNase Z^L^	Cucsa.359360	Phytozome	977
*Eucalyptus grandis*	EgrTRZ1	TM-type tRNase Z^S^	Egrandis_v1_0.020509 m.g	Phytozome	309
*Eucalyptus grandis*	EgrTRZ2	TM-type tRNase Z^S^	Egrandis_v1_0.021229 m.g	Phytozome	357*
*Eucalyptus grandis*	EgrTRZ3	tRNase Z^L^	Egrandis_v1_0.001658 m.g	Phytozome	971
*Glycine max*	GmaTRZ1	TM-type tRNase Z^S^	Glyma20g01480	Phytozome	279
*Glycine max*	GmaTRZ2	TM-type tRNase Z^S^	ACU18735.1	NCBI	353
*Glycine max*	GmaTRZ3	tRNase Z^L^	Glyma13g43270	Phytozome	923*
*Glycine max*	GmaTRZ4	tRNase Z^L^	Glyma15g02070	Phytozome	931*
*Mimulus guttatus*	MguTRZ1	TM-type tRNase Z^S^	mgv1a010642 m.g	Phytozome	306
*Mimulus guttatus*	MguTRZ2	TM-type tRNase Z^S^	mgv1a024577 m.g	Phytozome	364*
*Mimulus guttatus*	MguTRZ3	tRNase Z^L^	mgv1a000815 m.g	Phytozome	976
*Vitis vinifera*	VviTRZ1	TM-type tRNase Z ^S^	XP_002273058.1	NCBI	290
*Vitis vinifera*	VviTRZ2	TM-type tRNase Z ^S^	XP_002277241.1	NCBI	354
*Vitis vinifera*	VviTRZ3	tRNase Z^L^	XP_002278956.1	NCBI	951

**MONOCOTS**					
*Oryza sativa japonica*	OsaTRZ1	TM-type tRNase Z^S^	NP_001046280.1	NCBI	302
*Oryza sativa japonica*	OsaTRZ2	TM-type tRNase Z^S^	LOC_Os09g30466	Phytozome	365
*Oryza sativa japonica*	OsaTRZ3	tRNase Z^L^	B9EUI7	UniProt	964
*Zea mays*	ZmaTRZ1	TM-type tRNase Z^S^	GRMZM2G147727	Phytozome	302
*Zea mays*	ZmaTRZ2	TM-type tRNase Z^S^	ACG47847.1	NCBI	357
*Zea mays*	ZmaTRZ3	tRNase Z^L^	GRMZM2G379286	Phytozome	930

**MOSSES**					
*Physcomitrella patens*	PpaTRZ1	TM-type tRNase Z^S^	XP_001785449.1	NCBI	295
*Physcomitrella patens*	PpaTRZ2	TM-type tRNase Z^S^	Pp1s17_61V6	Phytozome	420
*Physcomitrella patens*	PpaTRZ3	Bacterial-type tRNase Z^S^	Pp1s409_46V6	Phytozome	336*
*Physcomitrella patens*	PpaTRZ4	tRNase Z^L^	Pp1s126_39V6	Phytozome	984*
*Physcomitrella patens*	PpaTLP1	tRNase Z^L^-like	Pp1s3_568V6	Phytozome	923*

**LYCOPHYTES**					
*Selaginella moellendorffii*	SmoTRZ1	TM-type tRNase Z^S^	167717	Phytozome	299
*Selaginella moellendorffii*	SmoTRZ2	tRNase Z^L^	111409	Phytozome	771*
*Selaginella moellendorffii*	SmoTRZ3	tRNase Z^L^	97243	Phytozome	819*
*Selaginella moellendorffii*	SmoTLP1	tRNase Z^S^-like	404484	Phytozome	303
*Selaginella moellendorffii*	SmoTLP2	tRNase-like	XP_002967989	NCBI	346
*Selaginella moellendorffii*	SmoTLP3	tRNase Z^L^-like	416540	Phytozome	731

**GREEN ALGAE**					
*Chlamydomonas reinhardtii *	CreTRZ1	TM-type tRNase Z^S^	Cre10.g431400	Phytozome	335
*Chlamydomonas reinhardtii*	CreTRZ2	tRNase Z^L^	Cre01.g039900	Phytozome	880*
*Chlamydomonas reinhardtii*	CreTLP1	tRNase Z^S^-like	Cre12.g492250	Phytozome	425*
*Chlamydomonas reinhardtii*	CreTLP2	tRNase Z^S ^-like	Cre07.g321900	Phytozome	378
*Chlamydomonas reinhardtii*	CreTLP3	tRNase Z^L^-like	Cre01.g068350	Phytozome	710*
*Chlamydomonas reinhardtii*	CreTLP4	tRNase Z^L^-like	Cre09.g394700	Phytozome	1073
*Micromonas pusilla *	MpuTRZ1	TM-type tRNase Z^S^	5692	JGI	383*
*Micromonas pusilla *	MpuTRZ2	Bacterial-type tRNase Z^S^	18700	JGI	398*
*Micromonas pusilla *	MpuTRZ3	tRNase Z^L^	54013	JGI	953*
*Micromonas pusilla *	MpuTLP1	tRNase Z^L^-like	C1N4P0	UniProt	866
*Ostreococcus lucimarinus*	OluTRZ1	TM-type tRNase Z^S^	26345	JGI	313
*Ostreococcus lucimarinus*	OluTRZ2	Bacterial-type tRNase Z^S^	XP_001416263	NCBI	369*
*Ostreococcus lucimarinus*	OluTRZ3	tRNase Z^L^	A4S9E8	UniProt	781*
*Ostreococcus lucimarinus*	OluTLP1	tRNase Z^L^-like	A4RR41	UniProt	780
*Volvox carteri*	VcaTRZ1	TM-type tRNase Z ^S^	80918	Phytozome	308*
*Volvox carteri*	VcaTRZ2	tRNase Z^L^	118548	Phytozome	881*
*Volvox carteri*	VcaTLP1	tRNase Z^S^-like	103834	Phytozome	445*
*Volvox carteri*	VcaTLP2	tRNase Z^L^-like	100314	Phytozome	723*

^**+**^The number of amino acids in plant tRNase Z and tRNase Z-like proteins.

*Indicates that mispredicted sequences obtained from the databases have been corrected.

**Figure 1:**
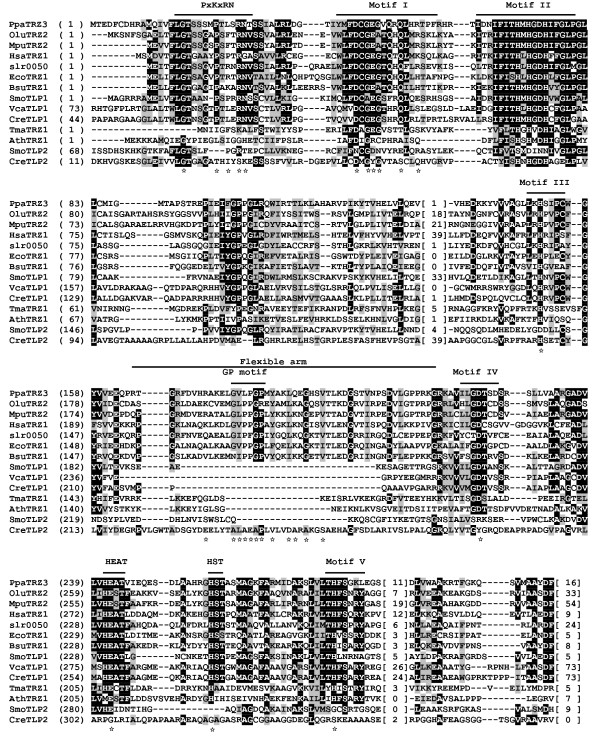
**Alignment of bacterial-type and tRNase Z^S^-like candidates in green plants**. Bacterial-type tRNase Z^S^s are from *P. patens *(PpaTRZ3), *O. lucimarinus *(OluTRZ2), *M. pusilla *(MpuTRZ2), *Synechocystis *sp. PCC 6803 (slr0050), *E. coli *(EcoTrz1) [72], *B. subtilis *(BsuTrz1) [60] and humans (HsaTrz1) [14]. Plant tRNase Z^S^-like (TLP) candidates are from *S*. *moellendorffii *(SmoTLP1 and SmoTLP2), *V. carteri *(VcaTLP1), *C. reinhardtii *(CreTLP1 and CreTLP2). TM-type tRNase Z^S^s from *T. maritima *(TmaTrz1) [30] and *A. thaliana *(AthTRZ1) [20] are included for comparison. The alignment was constructed using Clustal W [68]. Identical residues are on a black ground and conserved residues shared in gray. Also indicated above the alignment are the conserved motifs of tRNase Zs, which are labeled according to references [33,40,41]. The numbers in brackets indicate the length of the region in the protein, which are species-specific and could not be correctly aligned. Hyphens represent gaps introduced into sequences for maximum alignment. Amino acid residues predicted to be critical for activity are indicated by a star.

Interestingly, tRNase Z-like proteins (TLP) which apparently lack one or more conserved motifs of tRNase Zs necessary for the enzymatic activity of the protein are widespread in basal land plant and green algal genomes that have been analyzed here (Table 1 and Additional file 1). Many tRNase Z-like proteins appear to lack the flexible arm but contain all other conserved motifs of tRNase Zs (Figure 1 and see below for a more detailed discussion of the motifs). At one extreme, one *S. moellendorffii *tRNase Z-like protein (SmoTLP2) appears to lack all of the conserved motifs.

Based on their sizes, tRNase Z-like proteins can be divided into tRNase Z^S^-like and tRNase Z^L^-like proteins which are comparable in size to tRNase Z^S^s and tRNase Z^L^s, respectively. The number and form of tRNase Z-like proteins vary among the species, being largest in *C. reinhardtii *(two tRNase Z^S^-like and two tRNase Z^L^-like proteins) and next largest in *S. moellendorffii *(two tRNase Z^S^-like and one tRNase Z^L^-like proteins). The basal land plant *P. patens *and the two green algae *M. pusilla *and *O. lucimarinus *contain one tRNase Z^L^-like protein, whereas the green alga *V. carteria *contains one tRNase Z^S^-like and one tRNase Z^L^-like proteins. In contrast, black cottonwood (*P. trichocarpa*) appears to be the only species among the flowering plant genomes examined that contains the tRNase Z^L^-like protein. This species contains one tRNase Z^L^-like protein in which the second His in the His motif is mutated to Gln (data not shown).

### Prediction of subcellular localization of candidate tRNase Zs from flowering plants

To help understand the function of candidate tRNase Zs, we predicted *in silico *the subcellular localization of each of flowering plant tRNase Zs using different bioinformatic prediction programs. Most flowering plants have two tRNase Z^S^s (tRNase Z^S1 ^and tRNase Z^S2^). All tRNase Z^S1 ^proteins apparently lack any predictable signal sequences and therefore predicted to be cytoplasmic proteins, with the exception of *O. sativa *tRNase Z^S1 ^(OsaTRZ1), which is predicted to be localized in the chloroplasts (Table 2). On the other hand, all tRNase Z^S2 ^except *P. trichocarpa *tRNase Z^S2 ^(PtrTRZ2), contain a predicted chloroplast targeting signal (Table 2). The predicted chloroplast targeting signal of PtrTRZ2 is unusual in that it is only seven residues long since the majority of the known chloroplast targeting signals consist of N-terminal 20-80 amino acid residues [53]. In the two flowering plants (*S. bicolor *and *S. italica*) carrying multiple tRNase Z^S^s, only one tRNase Z^S ^(tRNase Z^S2^) from each species is predicted to be chloroplast-localized, whereas the rest are predicted to be cytoplasmic (Table 2).

**Table 2:** Prediction of the chloroplast targeting signals in candidate TM-type tRNase Z^S^s from flowering plants

Protein name	Form	Chl	Chloroplast targeting signal
AcoTrz1	AcoTrz^S1^	-	
AcoTrz2	AcoTrz^S2^	C	MKISLGISTSPILLPFSKPTLKIPHNRVCIP (31)
AlyTrz1	AlyTrz^S1^	-	
AlyTrz2	AlyTrz^S2^	C	MQLSSFPISPSKIFPFTKYHAPPVIIHQLAAQIQSHRSNFVSPVKVSGYFSSISRAIEEEEEYRKAR (67)
AthTrz1	AthTrz^S1^	-	
AthTrz2	AthTrz^S2^	C	MQLSSSFPISPPKIFPSTKHHKPPVITHQLAAQIQSNRRHFVSPVKVSGYFSSISRAIEEEEEYRKAR (68)
BdiTrz1	BdiTrz^S1^	-	
BdiTrz2	BdiTrz^S2^	C	MATISLFSLPPLRLTRGLLSPSSGPASRFQTL (32)
CpaTrz1	CpaTrz^S1^	-	
CpaTrz2	CpaTrz^S2^	C	MLTSVPILPSKLQSVFPFGHHHYHSISHKPTPIHQFSFLV (40)
CclTrz1	CclTrz^S1^	-	
CclTrz2	CclTrz^S2^	C	MQLSLPTSPSKLPTIFPFHPSSIPKTPQSPHHLSLQSHVGPLNALKSAGFLSSISRAIDEEEEYRKAR (68)
CsiTrz1	CsiTrz ^S1^	-	
CsiTrz2	CsiTrz ^S2^	C	MQLSLPTSPSKLPTIFPFHPSSIPKTPQSPHHLSLQSHVGPLNALKSAGFLSSISRAIDEEEEYRKAR (68)
CsaTrz1	CsaTrz^S1^	-	
CsaTrz2	CsaTrz^S2^	C	MQISIPISPLRPPQVFPFHQPLLHTPKPPGVALQSHLNPVNSFRDSGLLSTIGVE (55)
EgrTrz1	EgrTrz^S1^	-	
EgrTrz2	EgrTrz^S2^	C	MRISIPISPSKSPQIFPFHHHHRRRHQIPDLRRRPPALSLPASAVNPLKSSGYLSTIHR (59)
GmaTrz1	GmaTrz^S1^	-	
GmaTrz2	GmaTrz^S2^	C	MQISLSDLAFKTPQLFPIHHPIFPPKPPLNHQVST (35)
MesTrz1	MesTrz^S1^	-	
MesTrz2	MesTrz^S2^	C	MQTSFPVSPSKIPSIFPFNHPILHNPTAPTNHHQLPLQTYLKRPINSTSLKSSGFLSAIGRAIEEEEEYKKAR (73)
MtrTrz1	MtrTrz^S1^	-	
MtrTrz2	MtrTrz^S2^	C	MQIPLTNPTFKPPQIFPFHHTIPSPKQQLSFPT (33)
MguTrz1	MguTrz^S1^	-	
MguTrz2	MguTrz^S2^	C	MITLNPTTSNLLNLHPFHPTPATHKHHLPPQTRRSVIACVSDAVV (45)
OsaTrz1	OsaTrz^S1^	C	MANSGKSSPAATSTTAPPPGRPKAKAPPLTVEGYPVEGISIGGQETCVIFPT (52)
OsaTrz2	OsaTrz^S2^	C	MAATSLLSLPSLRLTHRLLVPASSSAPASRSQFQTL (36)
PtrTrz1	PtrTrz^S1^	-	
PtrTrz2	PtrTrz^S2^	C	MQISIPL (7)
PpeTrz1	PpeTrz^S1^	-	
PpeTrz2	PpeTrz^S2^	C	MQIFLTISPCKAPLILPFHPPISKTPKTQRTSLKTLSKPYRLARSQVLRKGV (52)
RcoTrz1	RcoTrz^S1^	-	
RcoTrz2	RcoTrz^S2^	C	MQTSLPISHSKFPSIFPFNHPISHKPTTTR (30)
SitTrz1	SitTrz^S1^	-	
SitTrz2	SitTrz^S2^	C	MATASLFSPPSLRLLSRTTARLSRFQTLAARKPP (34)
SitTrz3	SitTrz^S3^	-	
SitTrz4	SitTrz^S4^	-	
SbiTrz1	SbiTrz^S1^	-	
SbiTrz2	SbiTrz^S2^	C	MATASLFSLPSLRALSRTSARCSRFQTLAARKPVESSSSTATS (43)
SbiTrz3	SbiTrz^S3^	-	
VviTrz1	VviTrz^S1^	-	
VviTrz2	VviTrz^S2^	C	MQISLPFSTSKVPYLSPLPNPTPQPPLTIPKPHPKSYIT (39)
ZmaTrz1	ZmaTrz^S1^	-	
ZmaTrz2	ZmaTrz^S2^	C	MVTASLFSLPSLRVLSRTSAHGPRFQILAARKPVESSTATSGSRRGGSKGAGLLSVLDR (59)

Identification of the putative chloroplast targeting signals was made by using ChloroP http://www.cbs.dtu.dk/services/ChloroP/. Chl stands for the chloroplast. C indicates chloroplast localization. "-", the localization could not be predicted. The numbers refer to amino acid positions starting from the N-terminus.

Besides tRNase Z^S^, flowering plants also contain one (tRNase Z^L1^) or two (tRNase Z^L1 ^and tRNase Z^L2^) tRNase Z^L^s. Most tRNase Z^L1 ^proteins contain a predicted mitochondrial targeting signal between two putative translational initiation sites at the N-terminus and also have a nuclear localization signal (Table 3). Some tRNase Z^L2 ^proteins have a putative mitochondrial targeting signal, while others are predicted to have both nuclear and mitochondrial targeting signals. The length of the putative mitochondrial targeting signals found in these candidates are within the expected size-range (20-80 aa) [54]. Consistent with the prediction, *A. thaliana *tRNase Z^L1 ^(AthTRZ3) was found in both the nucleus and the mitochondria [20]. However, *A. thaliana *tRNase Z^L2 ^(AthTRZ4) predicted to have both nuclear and mitochondrial targeting signals is actually found only in the mitochondria [20].

**Table 3:** Prediction of the nuclear and mitochondrial targeting sequences in tRNase Z^L^s from flowering plants

Protein name	From	Nuc	Mito	Predicted mitochondrial targeting sequences
AcoTrz3	AcoTrz^L1^	N	-	
AcoTrz4	AcoTrz^L2^	-	M	MPQISSNLKLFFSKTNHSPLFQFSFKASFCSSFLLSSKTPYKPLSSVTVISSSSSSSSSSRKGPKFPPLRSRS (73)
AlyTrz3	AlyTrz^L1^	N	M	MINSMPYLHKNLRLLLLLSSKSSPFPLSLRPFSPRSFSLSTLFS (44)
AlyTrz4	AlyTrz^L2^	N	M	MLTSSMPHRHVPQNLSLFGFSPLKSSSFALFLRPFSLYPPIFASSSPSPSRRPPRTAGYRRS (62)
AthTrz3	AthTrz^L1^	N	M	MINSMPYLHKNLRLLRLLSSKSSPFPLSLRPFSPRSFSLSTLFS (44)
AthTrz4	AthTrz^L2^	N	M	MLTSSMPQNLSLFGFSPLKSSSFALILRPFSLYPPIFASSSPAPSRRPPRTAGYRRSGPSPPRRK (65)
BdiTrz3	BdiTrz^L1^	-	M	MPQVAAPLRLLLPLSQTLAPPAPLLHLSRRLLSFCSPASFRRAASLRALAYRRSRHPEPRRG (62)
CpaTrz3	CpaTrz^L1^	N	M	MFLIYPNLRLLLNPPLFLFSKPNSTPLSLFTVFASSSHKRHRSVSYRDSPFGLHRRRRNFTTFKERD (67)
CclTrz3	CclTrz^L1^	N	M	MPFITPNLRLLFSSSSSSLFPLKLSVPLLSTKPTNRHRSLFTILSYSKRQRSTPFPQQNQRRN (63)
CsiTrz3	CsiTrz^L1^	N	M	MPFITPNLRLLFSSSSSSSLFPLKLSVPLLSTKPTNRHHSLFTILSYSKRQRSTPFPQQNQRRN(64)
CsaTrz3	CsaTrz^L1^	N	M	MPLPHLSTLRFLFFSPSKLPFSPSLYSPKSHSLFTVLASSPPKRRRSATAPPSLNFKRRN (60)
EgrTrz3	EgrTrz^L1^	N	M	MPCVYSNLRLLFSSSATAAAATATAAASPFLSPLKLKLRRPSSSSSAFPLLPLPPLSSLRS (61)
GmaTrz3	GmaTrz^L1^	N	M	MAQVSKFGYFLLHSSLPKPSNIQFRSLLTVLASSSKRHRRK (41)
GmaTrz4	GmaTrz^L2^	N	M	MAQVSKFGHLLLHSSLPKPSNSNIQFRSLLTLLASSSKRHRSIPPFRRKS (50)
MesTrz3	MesTrz^L1^	N	M	MPQISNLRFLLSPIKPSLPFPFSKPKPYSLFTVLCSSSSSRRHRTTPNHQSLNFRSRS (58)
MesTrz4	MesTrz^L2^	-	M	MPNVLNFKLCLSDLLTQCCKICLQHYILTFSSLSHVLVSGKLQSSNPQTYFSINNPLPRSKNSRT (65)
MtrTrz3	MtrTrz^L1^	N	M	MAQILNFRNFLFLPSYKPTTHFRLRFLSTLVSSSSRRSNINAPPLHLRRRS (51)
MguTrz3	MguTrz^L1^	N	M	MPQSTNLRLLLSSANCHRRHPFSAASNFFPKHLSFSSSFQFFLKPQFKTREIPLLFATFSSYSKKPYATNNNSNNNNKNSRSFNRN (86)
OsaTrz3	OsaTrz^L1^	-	M	MPQLPSPLRRLLPLSQTLAAATPAPLLHLSRRLFSSSSSPSPSPSPRAACLRALAYRGGQAGGGGRRGHHNNLLRRG (77)
PtrTrz3	PtrTrz^L1^	N	M	MSHISNLRLLLSPLNPTLRFPFSSKHRPYSLLTILSSSSPYPKRRHRTTPNHPSLNFRSRS (61)
PpeTrz3	PpeTrz^L1^	N	M	MPQVTNLRLLFFSPFPRLSLSSLSFKPLKPRTLFTALASSYRKRHRPIPNQSPNTGARN (59)
SitTrz5	SitTrz^L1^	-	M	MPQVAAPLRLLLPLSQTLAPAPLLHLSRRLFTSSSPSFGRAASLRALAYRRHHHPRRGSSTLRK (64)
SbiTrz4	SbiTrz^L1^	N	M	MPQVAGPLRRLLPLSQTLASAPAPLLHLSRRLLSSCSPASFGRAASLRALAYRRRRHPEPRRG (63)
VviTrz3	VviTrz^L1^	N	M	MPHLTSFRLLYCSPLLSPFKSPFLSFSTLSKSKSPLLNPPSFFTVLSSSSGRYPKLRRHPHHLRRRN (67)
ZmaTrz3	ZmaTrz^L1^	N	M	MPQVAAPLRLLLPLSQTLAAPAPLLHHSRRLLSSCPASLSRAAGLRALAYRRRRHTEPRRG (61)

The putative nuclear localization signals were predicted using PSORT http://psort.hgc.jp/form.html, while the putative mitochondrial targeting signals were predicted using MITOPROT http://ihg2.helmholtz-muenchen.de/ihg/mitoprot.html. Nuc: stand for the nucleus; Mito: mitochondria. N indicates nuclear localization and M denotes mitochondrial localization. "-", the localization could not be predicted. The numbers refer to amino acid positions starting from the N-terminus.

### Phylogenetic analysis

**Figure 2:**
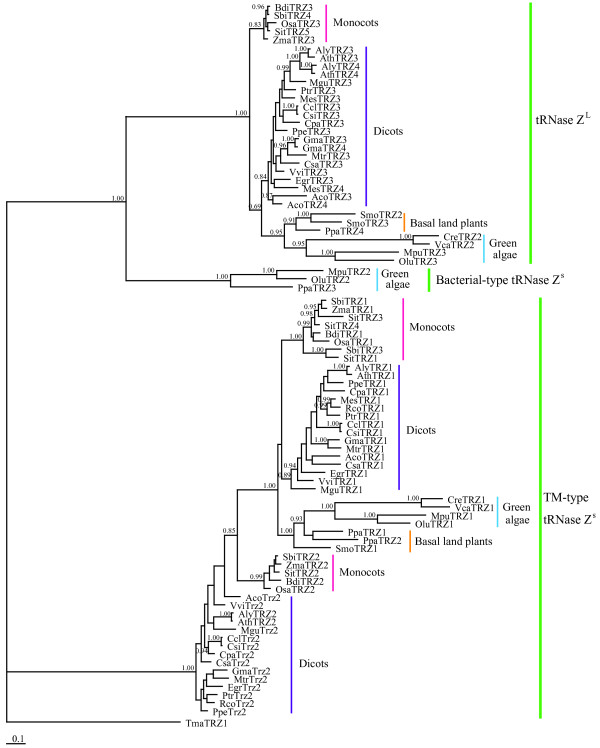
**Phylogram showing phylogenetic relationships among candidate green plant tRNase Zs**. Posterior probabilities, which are indicated at the nodes, are generated by the Bayesian analysis. The scale bar indicates 0.1 nucleotide substitutions per site. For each protein, the species, the accession number and the database can be found in Additional file 1. Taxonomic designations are indicated on the right side of the tree.

To gain insights into the evolutionary relationship among plant tRNase Zs, we reconstructed the phylogeny of 86 candidate sequences using Bayesian phylogenetics. Although most of plant species are flowering plants, they are taxonomically diverse. In addition to tRNase Zs from plants, we also include tRNase Z^S ^from *T. maritima *as outgroup. Phylogenetic analysis reveals the presences of two well-supported clades: one formed by including all TM-type tRNase Z^S^s, and the other all tRNase Z^L^s and bacterial-type tRNase Z^S^s (Figure 2). The latter clade is further divided into two subclades, of which one contains all tRNase Z^L^s, and the other bacterial-type tRNase Z^S^s. Notably, two tRNase Z^L^s (AlyTRZ4 and AthTRZ4) from the two *Arabidopsis *species (*A. thaliana *and *A. lyrata*) together form a group sister to a group formed by another two tRNase Z^L^s (AlyTRZ3 and AthTRZ3) from the same two species. Likewise, the two tRNase Z^L^s found in some species including *Aquilegia coerulea *(AcoTRZ3 and AcoTRZ4), *Glycine max *(GmaTRZ3 and GmaTRZ4) and *S. moellendorffii *(SmoTRZ2 and SmoTRZ3) are sister to each other with a posterior probability value of 1. These observations suggest that tRNase Z^L ^gene duplication took place in certain species.

### Conservation of candidate green plant tRNase Z^S^s

**Figure 3:**
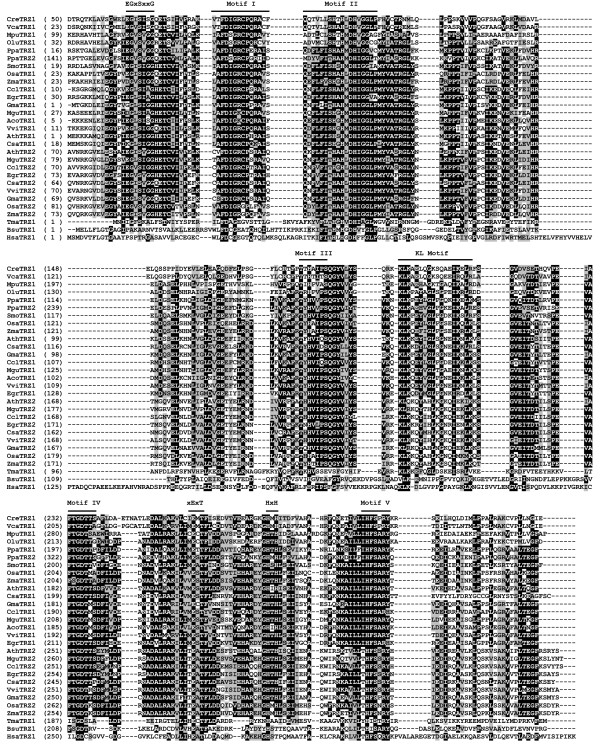
**Amino acid sequence alignment of the representative TM-type green plant and non-green plant tRNase Z^S^s**. Plant TM-type tRNase Z^S^s are from *C. reinhardtii *(CreTRZ1), *V. carteri *(VcaTRZ1), *M. pusilla *(MpuTRZ1) *O. lucimarinus *(OluTRZ1), *P. patens *(PpaTRZ1 and PpaTRZ2), *S. moellendorffii *(SmoTRZ1), *O. sativa japonica *(OsaTRZ1 and OsaTRZ2), *Z. mays *(ZmaTRZ1 and ZmaTRZ2), *A*. *thaliana *(AthTRZ1 and AthTRZ2) [20], *C. sativus *(CsaTRZ1 and CsaTRZ2), *G. max *(GmaTRZ1 and GmaTRZ2), *C. clementina *(CclTRZ1 and CclTRZ2), *M. guttatus *(MguTRZ1 and MguTRZ2), *A*. *coerulea *(AcoTRZ1), *V. vinifera *(VviTRZ1 and VviTRZ2) and *E. grandis *(EgrTRZ1 and EgrTRZ2). Non-green plant tRNase Z^S^s are from *T. maritima *(TmaTRZ1) [30], *B. subtilis *(BsuTRZ1) [60], humans (HsaTRZ1) [14]. Protein accession numbers are shown in Table 1. Alignment presentations are as described in the legend to Figure 1.

To assess the presence and conservation of motifs in candidate green plant tRNase Zs, we performed multiple sequence alignments of identified tRNase Zs sequences. Candidate tRNase Z^S^s are analyzed first. A list of aligned tRNase Z^S^s from representative green plants is shown in Figures 1 and 3. For comparison, tRNase Z^S^s from *T. maritima*, *B. subtilis*, *E. coli*, the cyanobacterium *Synechocystis *sp. PCC 6803 and humans are included as needed. A full list of all aligned green plant tRNase Z^S^s is presented in Additional file 4.

Sequence comparison reveals that except for the N-terminal regions, tRNase Z^S^s from the flowering plants appear to be more similar to each other than to either the basal land plants or green algae (Figure 3). Furthermore, the sequences of the green plant tRNase Z^S^s are highly divergent from those of *T. maritima*, *B. subtilis*, *E. coli *and human tRNase Z^S^s. For example, *A. thaliana *tRNase Z^S1 ^(AthTRZ1) and tRNase Z^S2 ^(AthTRZ2) exhibit only 17% - 24% and 16% - 21% identity (25% - 35% and 27% - 31% similarity), respectively, with those from *T. maritima*, *B. subtilis*, *E. coli *and humans. Overall, the sequence conservation between the green plant tRNase Z^S^s and non-plant species is largely confined to the highly conserved motifs of tRNase Zs.

**Figure 4:**
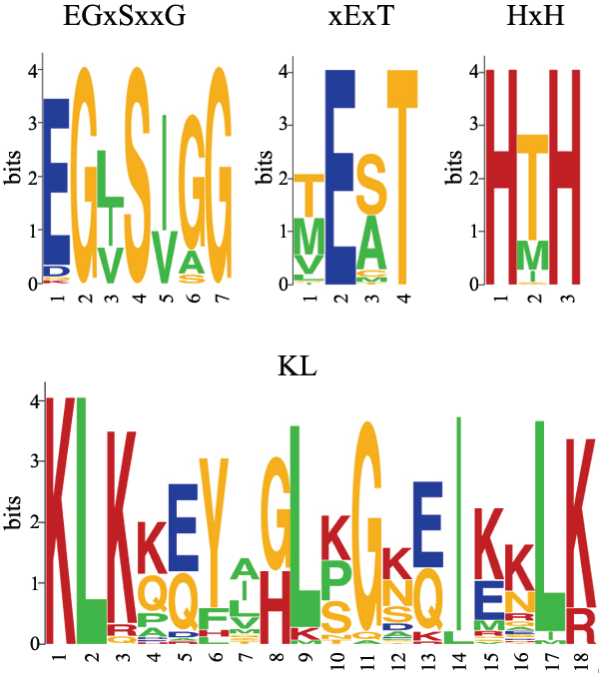
**Sequence logos of unique motifs found in candidate TM-type tRNase Z^S^s from green plants**. The sequence logos of motifs specific to candidate TM-type tRNase Z^S^s from green plants were derived from the 51 alignments. The sequence logos were created using WebLogo http://weblogo.berkeley.edu. The height of each amino acid indicates the level of conservation at that position. Amino acids are colored as follows: red, basic; blue, acidic; orange; polar; and green, hydrophobic.

Unexpectedly, careful examination of the sequences of green plant tRNase Z^S^s reveals that most of the proteins possess several unique features that distinguish them from bacterial-type tRNase Z^S^s and thus justify their classification as TM-type tRNase Z^S^. The sequence logos for motifs unique to TM-type tRNase Z^S^s are presented in Figure 4. First, most green plant tRNase Z^S^s harbor the TM type flexible arm. The plant TM-type flexible arms show only weak protein sequence homology to the bacterial-type flexible arms, and have distinctive features including the absence of the GP motif and the presence of a consensus sequence KLKxxYxxLxGxxIxxLK, here termed the KL motif (Figures 3 and 4). This Lys- and Leu-rich motif was previously unappreciated in *A. thaliana*, likely due to the limited number of plant sequences available at the time analysis was performed [39]. As might be expected, there are variations in the consensus sequence (Figures 3 and 4).

Second, most green plant tRNase Z^S^s lack the PxKxRN motif normally present on the amino side of Motif I. Instead, they share a Gly-rich consensus sequence EGxSxxG in an analogous position to the PxKxRN motif (Figure 3). In some cases, variants in the consensus sequence are found (Figures 3 and 4). Notably, there is very weak sequence similarity between this motif and the corresponding region in *T. maritima *tRNase Z^S^.

Third, most green plant tRNase Z^S^s contain conserved xExT and HxH motifs in place of the HEAT and HST motifs, respectively. The xExT motif is related to the HEAT motif except that the invariant His is replaced by a Thr/Met/Val/Leu residue and the invariant Ala is often replaced by Ser, or occasionally Met, Thr and Cys (Figures 3 and 4). In contrast, the HxH motif is related to the HST motif, except that the conserved Ser is mostly replaced by Thr, and the conserved Thr is substituted by His (Figures 3 and 4). Based on the structural and mutagenesis studies, it has been suggested that the Glu of the HEAT motif and His of the HST motif play a role in facilitating proton transfer at the final stage of reaction [25,29,40]. In particular, the His residue is implicated as the proton donor. It is highly likely that the conserved Glu-His pair from the xExT and HxH motifs in the TM-type tRNase Z^S^s may also participate in the terminal proton transfer reaction.

Multiple sequence alignment of bacterial-type tRNase Z^S^s from the basal plant *P. patens *(PpaTRZ3) and green algae reveals that in addition to the well conserved Motifs I-V, these candidates possess a bacterial-type flexible arm containing the GP, HEAT and HST motifs, and the variant PxKxRN motif, in which Lys is replaced with Leu (Figure 1).

### Conservation of tRNase Z^L^s in green plants

**Figure 5:**
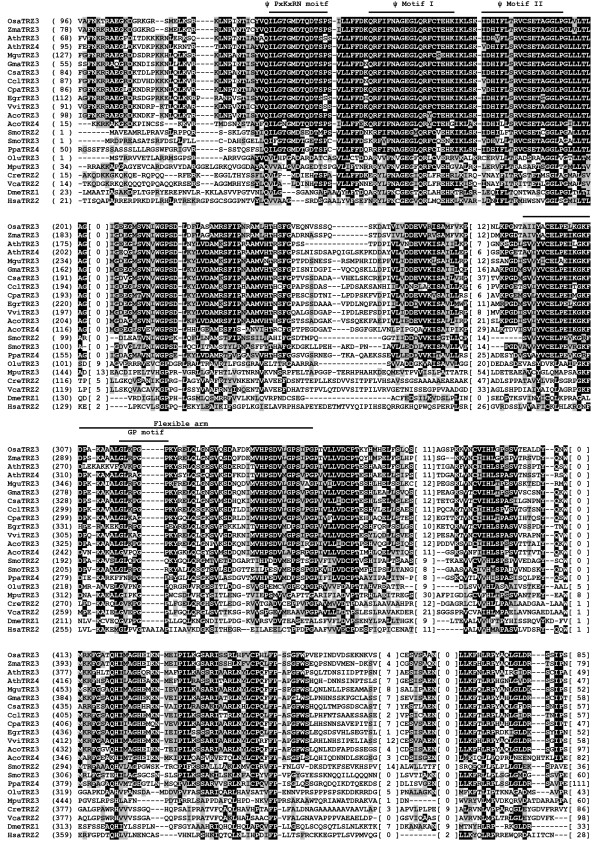
**Sequence alignment of N-terminal halves of representative green plant and non-green plant tRNase Z^L^s**. Green plant tRNase Z^L^s are from *O. sativa japonica *(OsaTRZ3), *Z. mays *(ZmaTRZ3), *A. thaliana *(AthTRZ3 and AthTRZ4) [20], *M. guttatus *(MguTRZ3), *G. max *(GmaTRZ3), *C. sativus *(CsaTRZ3), *C. clementina *(CclTRZ3), *C. papaya *(CpaTRZ3), *E. grandis *(EgrTRZ3), *V. vinifera *(VviTRZ3), *A. coerulea *(AcoTRZ3 and AcoTRZ4), *S. moellendorffii *(SmoTRZ2 and SmoTRZ3), *P. patens *(PpaTRZ4), *O. lucimarinus *(OluTRZ3), *M. pusilla *(MpuTRZ3), *Chlamydomonas reinhardtii *(CreTRZ2) and *V. carteri *(VcaTRZ2). Non-green plant *D. melanogaster *(DmeTRZ1) [16] and humans (HsaTRZ2) [14] are included for comparison. The annotation of the alignment is as described in the legend to Figure 1.

**Figure 6:**
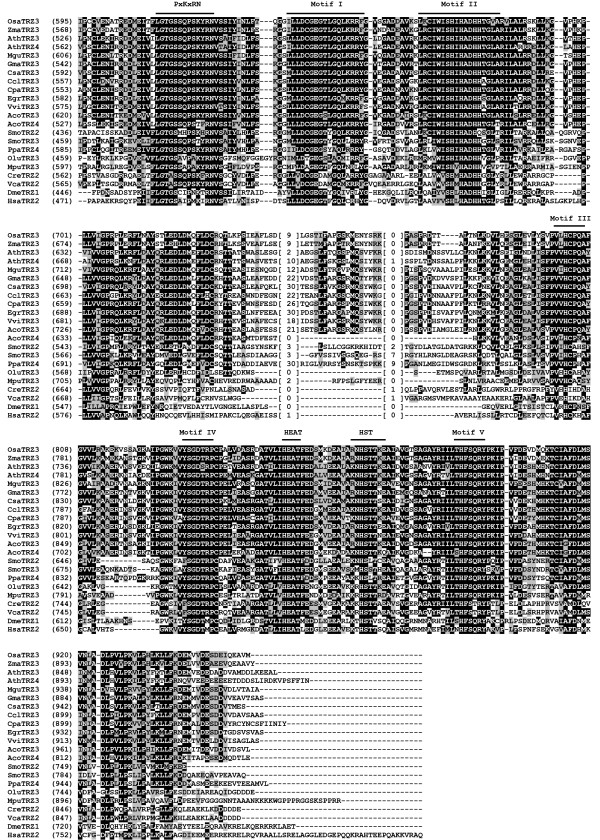
**Sequence alignment of C-terminal halves of representative green plant and non-green plant tRNase Z^L^s**. Same legend as in Figure 5.

Since tRNase Z^L ^can be divided into the N-terminal and C-terminal halves, which are related by weak sequence similarity, we aligned these two halves separately. The alignment of the N-terminal and C-terminal halves of representative candidate tRNase Z^L^s from diverse green plant species are shown in Figures 5 and 6, respectively. Two non-plant eukaryotic tRNase Z^L^s from *D. melanogaster *and humans were included for comparison. A complete list of all aligned green plant tRNase Z^L^s identified here is given in Additional file 5.

Except for the N-terminal regions, candidate tRNase Z^L^s from land plants share a high degree of overall sequence similarity to each other. In contrast, candidate tRNase Z^L^s from the four green algae show relatively low overall sequence similarity to each other and to the land plant tRNase Z^L^s. In particular, their N-terminal halves are highly divergent compared to those from the land plants. Furthermore, sequence similarity between land plant tRNase Z^L^s and their homologs in green algae, *D. melanogaster *and humans is limited to the conserved motifs identified in tRNase Zs.

The N-terminal halves of all green plant tRNase Z^L^s contain a conserved eukaryotic-type flexible arm domain containing the GP motif. In addition, the N-terminal halves of the proteins also contain a pseudo-PxKxRN motif and pseudo-Motifs I and II, which differ from their original motifs in many positions including critical residues for tRNase Z functions (Figure 5). These pseudo-motifs were previously identified in candidate fungi tRNase Z^L^s [51]. In contrast, the C-terminal halves of green plant tRNase Z^L^s harbor conserved Motifs I-V and the PxKxRN, HEAT and HST motifs (Figure 6). These motifs appear in the same relative order in all the sequences in which they are present.

### Many chloroplast tRNA genes encode a portion of the 3'-CCA sequence

It has been suggested that many chloroplast tRNA genes encode partial CCA sequences [55]. However, this conclusion was primarily based on the examination of the 3'-flanking sequences of tRNA genes from three chloroplast genomes. To determine if this conclusion holds true when more chloroplast genome sequences are now available for analysis, we examined the presence of tRNA genes encoding whole or partial CCA sequences in 15 chloroplast genomes. The results obtained were tabulated in Table 4.

**Table 4:** The distribution of the trinucleotide sequences immediately after the discriminator nucleotide in chloroplast tRNA genes

Species	^**74**^**CCA**^**76**^	^**74**^**CCN**^**76**^	^**74**^**CNN**^**76**^	Total genes examined	%^+^
*Arabidopsis thaliana*	0	1	11	37	32
*Brachypodium distachyon*	1	1	8	38	26
*Carica papaya*	0	1	8	37	24
*Citrus sinensis*	2	1	7	37	27
*Cucumis sativus*	0	1	7	37	22
*Eucalyptus grandis*	0	1	8	36	25
*Glycine max*	0	1	9	37	27
*Manihot esculenta*	0	1	7	37	22
*Medicago truncatula*	0	0	5	29	17
*Oryza sativa japonica*	1	2	7	38	26
*Populus trichocarpa*	0	0	10	37	27
*Prunus persica*	0	1	8	37	24
*Sorghum bicolor*	0	2	7	38	24
*Vitis vinifera*	0	1	8	37	24
*Zea mays*	1	2	7	37	27

Nucleotide sequences of chloroplast tRNA genes and their 3' flanking regions were obtained from the GenBank genome database http://www.ncbi.nlm.nih.gov/genomes/genlist.cgi?taxid=2759&type=4&name=Eukaryotae%20Organelles, the Chloroplast Genome Database (ChloroplastDB; http://chloroplast.cbio.psu.edu/organism.cgi), and the Organelle Genome Database (GOBASE; http://gobase.bcm.umontreal.ca/searches/gene.php). The discriminator nucleotide was predicted using tRNAscan-SE 1.21 http://lowelab.ucsc.edu/tRNAscan-SE/. ^+^Percentage of chloroplast tRNA genes encoding ^74^CNN^76^, ^74^CCN^76 ^and ^74^CCA^76^.

Indeed, we found that many tRNA genes in the chloroplast genomes encode partial CCA sequences, which could serve as part of the CCA sequence (Table 4). For instance, in the *A. thaliana *chloroplast genome, 31% of tRNA genes encode the whole or partial CCA sequences. Moreover, many chloroplast tRNA genes encode the first base of the CCA sequence. For example, of the 37 *A. thaliana *chloroplast tRNA genes examined, 11 had C after the discriminator.

## Discussion

### The presence of multiple tRNase Zs in green plants

Unlike *C. elegans*, *D. melanogaster*, humans and most fungal species examined to date, green plants are unique in that they possess multiple tRNase Zs. Of the 27 complete green plant genomes analyzed, the majority of them encode two tRNase Z^S^s and one or two tRNase Z^L^s. It is reasonable to expect that the existence of multiple tRNase Zs would be common to green plants. This phenomenon appears to have arisen from genome doubling (polyploidy), which seems to be a driving force in plant evolution and variation [56]. However, there seems to be no correlation between the number of tRNase Zs and genome size (Table 5).

**Table 5:** The genome size and ploidy level of representative green plants and the number of tRNase Zs encoded by their genomes

Species	Common name	No. tRNase Z^#^	Ploidy level	Genome size (Mbp)	Refs
*Arabidopsis thaliana*	Mouse-ear cress	4	Diploid	125	[73]
*Brachypodium distachyon*	Purple false brome	3	Diploid	272	[74]
*Carica papaya*	Papaya	3	Diploid	372	[75]
*Cucumis sativus*	Cucumber	3	Diploid	367	[76]
*Glycine max*	Soybean	4	Diploid	1100	[77]
*Oryza sativa japonica*	Rice	3	Diploid	389	[78]
*Populus trichocarpa*	Black cottonwood or poplar	3	Diploid	485	[79]
*Ricinus communis*	Castor bean	3	Diploid	350	[80]
*Setaria italica*	Foxtail millet	5	Diploid	490	[81]
*Sorghum bicolor*	Sorghum	4	Diploid	730	[82]
*Vitis vinifera*	Grape	3	Diploid	505	[83]
*Zea mays*	Maize	3	Diploid	2300	[84]

The genome size and ploidy level were obtained from the Plant DNA C-values database http://www.kew.org/cvalues/.

^#^The number of tRNase Zs

**Figure 7:**
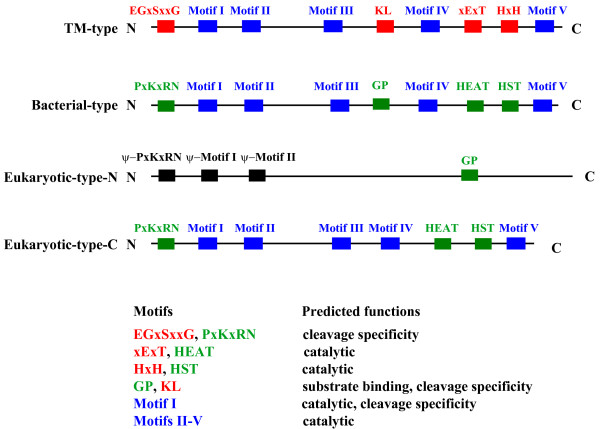
**Schematic representation of the three different types of tRNase Zs identified in green plants**. The N-terminal half (eukaryotic-type-N) and C-terminal half (eukaryotic-type-C) of the eukaryotic-type tRNase Z^L ^are shown separately. Motifs are indicated by colored boxes: red, specific to TM-type; green, specific to both bacterial- and eukaryotic-type; and blue, common to all three types. N and C denote N and C terminus, respectively. Relative positions of these motifs are not drawn to scale. The predicted functions of each motif are also indicated.

An unexpected observation in this study is that most green plant tRNase Z^S^s, which are clearly distinct from the bacterial-type tRNase Z^S^, represent the TM-type tRNase Z^S^. A comparison of motifs found in three different types of tRNase Zs from green plants is provided in Figure 7. Our phylogenetic analysis also supports the existence of both the TM- and bacterial-types of tRNase Z^S^s in green plants (Figure 2). The TM-type tRNase Z^S ^was previously found only in the hyperthermophilic bacterium *T. maritima *and the flowering plant *A. thaliana*, and thus, it was originally thought to be a minor type. Our data greatly expand the repertoire of this type of tRNase Z. Although it remains to be determined if the TM-type tRNase Z^S ^is also widespread in other taxonomic groups, the prevalence of the TM-type tRNase Z^S ^in green plants suggests that this type might be plant-specific.

In contrast to the TM-type tRNase Z^S^, which is widespread in the green plants, the bacterial-type tRNase Z^S ^is only present in the basal land plants and green algae. Sequence analysis reveals that the two types of tRNase Z^S^s are related to but distinct from each other, suggesting that they likely arose from the same ancestral tRNase Z^S ^gene, followed by sequence divergence. Since the flowering plants possess only the TM-type tRNase Z^S^, the bacterial-type tRNase Z^S ^has apparently been lost during the course of plant evolution whereas the TM-type tRNase Z^S ^has been maintained. Interestingly, the bacterial-type tRNase Z^S ^is also found in humans. Although the precise function of human tRNase Z^S ^is unknown, it may play a role in degradation of a subset of microRNAs (miRNA) in the cytoplasm [57].

It is likely that multiple tRNase Zs found in the flowering plants are localized in different subcellular compartments. In most of the flowering plants examined, the two TM-type tRNase Z^S^s (tRNase Z^S1 ^and tRNase Z^S2^) are predicted to be either in the cytosol or in the chloroplast, whereas one of tRNase Z^L ^(tRNase Z^L1^) is predicted to contain both nuclear and mitochondrial targeting signals. The second tRNase Z^L ^(tRNase Z^L2^) found in some plants either contains or lacks a predicted mitochondrial targeting signal. These predictions are generally supported by recent subcellular localization studies of *A. thaliana *tRNase Zs [20]. *A. thaliana *tRNase Z^S1 ^(AthTRZ1) and tRNase Z^S2 ^(AthTRZ2) are localized in the cytoplasm and chloroplast, respectively, whereas *A. thaliana *tRNase Z^L1 ^(AthTRZ3) and tRNase Z^L2 ^(AthTRZ4) are targeted to both the nucleus and mitochondria, and the mitochondria, respectively [20].

Based on their predicted subcellular localization, it is most likely that tRNase Z^S2 ^and tRNase Z^L1 ^are involved in the 3'-ends processing of pre-tRNAs in the chloroplasts, and both the nucleus and mitochondria, respectively, whereas tRNase Z^L2 ^either serves as a back-up for mitochondrial pre-tRNA 3'-end processing or plays a specialized, yet to be discovered function. The function of tRNase Z^S1 ^is unknown. It may play a role in repair of incorrectly processed 3'-ends of tRNAs in the cytosol. Alternatively, tRNase Z^S1 ^could also participate in nuclear pre-tRNA 3'-end processing, as suggested by the observation that deletion of both *A. thaliana *tRNase Z^S1 ^(AthTRZ1) and tRNase Z^L1 ^(AthTRZ3) results in a lethal phenotype [20].

### The plant TM-type tRNase Z^S^s may not recognize the CCA sequence as an anti-determinant

The CCA triplet following the discriminator generally inhibits tRNase Z activity, with the first C at nucleotide position 74 having the greatest effect [25,33,58]. This anti-determinant property of the CCA sequence is thought to prevent removal of the 3'-terminal CCA sequence from mature tRNAs, and thus avoids futile cycles of CCA addition and removal [33,58-60]. However, unlike other types of tRNase Zs, the TM-type tRNase Z does not appear to recognize the CCA sequence that is downstream the discriminator and present in the trailer sequence as an anti-determinant. The recombinant *T. maritima *tRNase Z^S ^cleaves after the CCA sequence which is encoded by all but one tRNA gene, leaving the CCA sequence intact [30]. Similarly, the recombinant *A. thaliana *tRNase Z^S ^can process pre-tRNAs with the 3'-terminal CCA sequences embedded in the trailer sequence, albeit at a different position [61]. Like the recombinant proteins, a partially purified spinach chloroplast tRNase Z fraction can also cleave pre-tRNAs containing complete or partial CCA sequences after the first C^74 ^regardless of the sequence of the flanking region [55]. Importantly, the remainder of the CCA sequence can be added by chloroplast tRNA nucleotidyltransferease [55].

However, *A. thaliana *tRNase Z^S ^appears to be unusual in that it can also cleave off the 3'-terminal CCA sequence from mature tRNAs *in vitro *[61]. Since mature tRNAs must be protected from counterproductive cleavage by tRNase Z^S ^*in vivo*, it is likely that the plant chloroplast protein may acquire the ability to recognize the 3'-terminal CCA sequence as the mature tRNA 3'-end via cofactors [61].

### Sequence determinants potentially involved in cleavage specificity

The mechanisms responsible for cleavage site selection and CCA inhibition are not fully understood, but appear to involve a combination of sequence features. First, the flexible arm has been suggested to contribute to cleavage site selection and the inhibitory effect of the CCA sequence [62]. This tRNase Z-specific element is located on the opposite side of the active site and binds primarily the D and T loops of the pre-tRNA [28,39,63]. Notably, the flexible arms of the *T. maritima *and plant tRNase Z^S^s lack the GP motif but contain the KL motif. Additionally, they are significantly smaller in length relative to those of other tRNase Zs possessing the anti-determinant function. Structural studies reveal that although TM- and bacterial-types of flexible arms share a similar overall structure which is composed of a compact globular domain and an extended two-stranded stalk and protrudes from the protein core, they have different globular domains [25-28,64]. In the TM-type flexible arm, the globular domain consists of one very short *α*-helix, one long helix and one 3_10_-helix, whereas in the bacterial-type flexible arm, it is composed of two α-helices, two *β*-strands and one 3_10_-helix. It would be interesting to know how the differences in the sequence feature, length and topology of the flexible arm may contribute to cleavage specificity by the enzymes.

Another possible motif involved in selection of the cleavage site has been suggested to be Motif I which appears to participate in binding the acceptor stem of pre-tRNA substrates [33,62]. In vitro studies using the recombinant *T. maritima *tRNase Z^S ^has suggested that the Ser31 and Thr33 residues of Motif I are involved in the cleavage site selection, with the former residue being more critical [30]. However, only individual, but not simultaneous mutations of these two non-Gln residues to Gln (which are found at the corresponding positions in other tRNase Zs with the CCA anti-determinant) in *T. maritima *tRNase Z^S ^affects the cleavage site selection [62].

In addition to the flexible arm and Motif I, the PxKxRN motif, in particular, the two basic residues in the motif, has also been suggested to be involved in the cleavage specificity and, by inference, CCA inhibition of tRNase Zs, since this motif is absent from *T. maritima *and *A. thaliana *[33]. Strikingly, tRNase Z^S^s found in all flowering plants examined so far also lack this motif (Figure 3). However, since the archaeon *Methanococcus jannaschii *tRNase Z^S ^harbors a PxKxRN motif but cannot recognize the CCA sequence as an anti-determinant *in vitr*o, suggesting that other sequence elements may also be involved in the cleavage specificity of tRNase Z [61]. Alternatively, it has been suggested that a high enzyme concentration used in the assay may contribute to the lack of a CCA anti-determinant effect with the *M. jannaschii *tRNase Z^S ^[33].

### Why is the TM-type tRNase Z^S ^developed for chloroplast pre-tRNA 3'-end processing?

The discovery that the TM-type tRNase Z^S ^is widespread in green plants raises a question as to why plants adopt the TM-type tRNase Z^S ^over other types of tRNase Z for the 3'-end processing of chloroplast pre-tRNAs. One possible explanation is that the TM-type tRNase Z^S ^identified in plants has evolved to adapt to chloroplast pre-tRNA 3'-end processing. It has previously been suggested that many plant chloroplast tRNA genes encode C^74 ^based on a limited number of available chloroplast tRNA genes [55]. To see if this conclusion could be extended to more flowering plants, we have examined the 3'-flanking region for tRNA genes in additional chloroplast genomes. Indeed, many chloroplast tRNA genes seem to encode partial CCA sequences (Table 4).

The development of the TM-type tRNase Z^S ^may be particularly important for chloroplasts, which have a limited but sufficient number of self-encoded tRNA species and do not import tRNAs [65,66]. As all possible codons are used in the chloroplast protein-encoding genes, all chloroplast tRNA species appear to be used in protein synthesis [66]. Thus, efficient 3'-end processing of each pre-tRNA by tRNase Z may be critical in ensuring maximum efficiency in chloroplast protein synthesis.

### The presence of candidate tRNase Z-like proteins in green plants

tRNase Z^S^-like proteins have previously been found in cyanobacteria including *Synechocystis *sp. PCC 6803 [67] and fungi [51], whereas tRNase Z^L^-like proteins have not been reported. In *Synechocystis*, one tRNase Z^S^-like protein (sll1036) has been identified. This protein does not exhibit any tRNase Z activity in vitro, consistent with the lack of some of the most conserved motifs of tRNase Zs in the protein sequence [67]. Since candidate tRNase Z-like proteins found in basal land plants and green algae either lack all or some of the conserved motifs of tRNase Zs, they most likely possess no tRNase Z activity.

It has been suggested that tRNase Z^L ^has arisen from the fusion of duplicated tRNase Z^S ^genes with further sequence diversification. It is possible that tRNase Z^S^-like proteins found in the basal land plants and green algae may represent relics of original tRNase Z^S ^that were mutated during diversification of eukaryotic tRNase Z genes. Alternatively, these proteins may play species-specific functions as suggested for cyanobacterial tRNase Z^S^-like proteins [67]. The predominant presence in the basal land plants and green algae of tRNase Z-like proteins also suggests that duplication of ancestral tRNase Z^S ^genes may occur early in green plant evolution.

## Conclusions

This study represents the first large-scale identification and analysis of green plant tRNase Zs. Our survey of current plant genome databases shows that green plants are represented by multiple tRNase Zs, which include one or two tRNase Z^L^s and two tRNase Z^S^s. One tRNase Z^L ^is predicted to participate in 3'-end processing of nuclear and mitochondrial pre-tRNA, whereas the other is likely to provide a backup for mitochondrial pre-tRNA processing. It appears that most tRNase Z^S^s, which is widespread throughout the green plants, belong to a minor but highly distinct type of tRNase Z^S ^(TM-type). In contrast, the typical bacterial-type tRNase Z^S ^is restricted to basal land plants and green algae. The apparent lack of the bacterial-type tRNase Z^S ^in flowering plants suggest that while both types were present in the basal land plants, the bacterial-type tRNase Z^S ^was discarded in favor of TM-type during plant evolution. Based on our results and previous studies, we propose that like *T. maritima *tRNase Z^S^, TM-type tRNase Z^S^s found in green plants seem not to recognize the CCA sequence as an anti-determinant and that the rise of this type of tRNase Z^S ^appears to accommodate the 3'-end processing of chloroplast pre-tRNAs with partial or whole CCA sequences. This unusual property of green plant tRNase Z^S^s is likely due to multiple sequence determinants including the TM-type-specific flexible arm comprising the KL motif, Motif I and lack of the PxKxRN motif.

However, it should be noted that bioinformatics analysis alone cannot resolve possible differences in cleavage specificity among TM-type tRNase Z^S^s. The complete understanding of the mechanisms of the cleavage specificities of the TM-type tRNase Z^S^s awaits the determination of the structures of these enzymes bound to pre-tRNA and the accumulation of more precise biochemical data.

## Methods

### Plant genome database search and protein sequence analysis

Candidate tRNase Zs were identified by BLAST and PSI-BLAST searches against the genome databases using known tRNase Zs as query sequences. The databases used include Phytozome http://www.phytozome.net/, the NCBI nonredundant protein sequence database http://blast.ncbi.nlm.nih.gov/Blast.cgi, Joint Genome Institute (JGI; http://www.jgi.doe.gov/ and Universal Protein Resource (Uniprot; http://www.uniprot.org/. An E-value cutoff of 0.001 was used in all searches. With this value, no *β*-CASP protein or other MBL protein was found. The resulting sequences were subject to validation as described [51]. The splicing pattern was verified using the FGENESH and FGENESH_GC programs provided at the Softberry website http://linux1.softberry.com/berry.phtml?topic=fgenesh. Prediction of subcellular localization of proteins was made using web-based prediction programs such as MITOPROT http://ihg2.helmholtz-muenchen.de/ihg/mitoprot.html, PSORT http://psort.hgc.jp/form.html and ChloroP http://www.cbs.dtu.dk/services/ChloroP/. Multiple sequence alignments were done by Clustal W [68].

### Phylogenetic analysis

Phylogenetic analysis was performed using the Bayesian approach, with *T. maritima *tRNase Z^S ^(TmaTrz1) as an outgroup as described [51]. Briefly, full-length amino acid sequences of candidate plant tRNase Zs and TmaTrz1 were aligned by using Clustal W implemented in MEGA 5.0 [69]. After excluding gaps and the ambiguous sites, we used ProtTest 2.4 [70] to choose the most appropriate evolutionary model for our data set. The phylogenies were estimated by Bayesian inference with MrBayes 3.1.2 [71] using a mixture of the fixed amino acid models and I + G distribution. Statistical confidence was assessed by using Markov Chain Monte Carlo (MCMC) sampling approaches. Two separate runs including a total of four independent tree searches were conducted. All searches consisted of one 'cold' and three 'heated' Markov chains estimated for 10^7 ^generations, and every 1000 generations were sampled. The burn-in parameter was estimated by plotting -ln*L *against the generation number using Tracer 1.4.1 http://beast.bio.ed.ac.uk/Tracer, and the retained trees were used to estimate the consensus tree and the Bayesian posterior probabilities.

## List of abbreviations

pre-tRNAs: tRNA precursors; miRNA: microRNAs; tRNase Z: tRNA 3' endonuclease; tRNase Z^S^: the short form of tRNase Z; tRNase Z^L^: the long form of tRNase Z; *β*-CASP: MBL-associated CISF Artemis SNM1/PSO2; CPSF-73: the cleavage and polyadenylation specificity factor; MBL: the metallo-*β*-lactamase superfamily; TGF-*β*: transforming growth factor-*β*; TM: *T. maritima*.

## Competing interests

The authors declare that they have no competing interests.

## Authors' contributions

LF, ZW and JL performed online database searches and sequence analysis. JY carried out a phylogenetic analysis. YH conceived this study, analyzed the data and drafted the manuscript. All authors have read and approved the final version of the manuscript.

## Supplementary Material

Additional file 1 **Distribution of candidate tRNase Zs identified in green plants**. Abbreviations for species names are indicated in the parentheses. ^**+**^The number of amino acids in plant tRNase Z and tRNase Z-like proteins. *Indicates that mispredicted sequences obtained from the databases have been corrected. ^?^Indicates the sequence could not be correctly predicted. Click here for file

Additional file 2 **Pairwise sequence comparisons of *S. bicolor *and *S. italica *tRNase Z^S^s**. The accession numbers for proteins are listed in Additional file 1. The pairwise percent identity (I) and percent similarity (S) between tRNase Z^S^s from *S. bicolor *and *S. italica *were calculated using the Clustal W program [68]. Click here for file

Additional file 3 **Alignment of candidate tRNase Z^S^s from *S. bicolor *and *S. italic***. The accession numbers for the candidates are listed in Additional file 1. The annotation of the alignment is described in the legend to Figure 1. Click here for file

Additional file 4 **Alignment of candidate TM-type tRNase Z^S^s identified in green plants**. The accession numbers for the candidates are listed in Additional file 1. Annotation is as described in the legend to Figure 1. Click here for file

Additional file 5 **Alignment of candidate tRNase Z^L^s identified in green plants**. The accession numbers for the candidates are listed in Additional file 1. The annotation of the alignment is as described in the legend for Figure 1. Click here for file
