# Supplementary material for: A survey of green plant tRNA 3'-end processing enzyme tRNase Zs, homologs of the candidate prostate cancer susceptibility protein ELAC2
Source: BMC Evol Biol. 2011 Jul 23;11:219. doi: 10.1186/1471-2148-11-219 (PMC3161902; doi:10.1186/1471-2148-11-219)
Supplement: Additional file 1 — Distribution of candidate tRNase Zs identified in green plants. Abbreviations for species names are indicated in the parentheses. +The number of amino acids in plant tRNase Z and tRNase Z-like proteins. *Indicates that mispredicted sequences obtained from the databases have been corrected. ?Indicates the sequence could not be correctly predicted. [file 1471-2148-11-219-S1.DOC]

Additional file 1: Candidate tRNase Zs identified in green plant species

| Species^#^ | Protein name | Type | Accession number | Database | No. aa^+^ |
| --- | --- | --- | --- | --- | --- |
| **DICOTS** |  |  |  |  |  |
| *Aquilegia coerulea* (Aco) | AcoTRZ1 | TM-type tRNase Z^S^ | AcoGoldSmith_v1.019418m.g | Phytozome | 283 |
| *Aquilegia coerulea* (Aco) | AcoTRZ2 | TM-type tRNase Z^S^ | AcoGoldSmith_v1.007495m.g | Phytozome | 356 |
| *Aquilegia coerulea* (Aco) | AcoTRZ3 | tRNase Z^L^ | AcoGoldSmith_v1.000749m.g | Phytozome | 997* |
| *Aquilegia coerulea* (Aco) | AcoTRZ4 | tRNase Z^L^ | AcoGoldSmith_v1.024294m.g | Phytozome | 848* |
| *Arabidopsis lyrata* (Aly) | AlyTRZ1 | TM-type tRNase Z^S^ | 476606 | Phytozome | 280 |
| *Arabidopsis lyrata* (Aly) | AlyTRZ2 | TM-type tRNase Z^S^ | 480092 | Phytozome | 353 |
| *Arabidopsis lyrata* (Aly) | AlyTRZ3 | tRNase Z^L^ | 474332 | Phytozome | 882* |
| *Arabidopsis lyrata* (Aly) | AlyTRZ4 | tRNase Z^L^ | 341756 | Phytozome | 946 |
| *Arabidopsis thaliana* (Ath) | AthTRZ1 | TM-type tRNase Z^S^ | NP_177608.2 | NCBI | 280 |
| *Arabidopsis thaliana* (Ath) | AthTRZ2 | TM-type tRNase Z^S^ | NP_178532.2 | NCBI | 354 |
| *Arabidopsis thaliana* (Ath) | AthTRZ3 | tRNase Z^L^ | NP_175628.2 | NCBI | 890 |
| *Arabidopsis thaliana* (Ath) | AthTRZ4 | tRNase Z^L^ | NP_188247.2 | NCBI | 942 |
| *Carica papaya* (Cpa) | CpaTRZ1 | TM-type tRNase Z^S^ | evm.TU.supercontig_17.217 | Phytozome | 306 |
| *Carica papaya* (Cpa) | CpaTRZ2 | TM-type tRNase Z^S^ | evm.TU.supercontig_132.51 | Phytozome | 359* |
| *Carica papaya* (Cpa) | CpaTRZ3 | tRNase Z^L^ | evm.TU.supercontig_34.31 | Phytozome | 943* |
| *Citrus clementina* (Ccl) | CclTRZ1 | TM-type tRNase Z^S^ | clementine0.9_017657m.g | Phytozome | 288 |
| *Citrus clementina* (Ccl) | CclTRZ2 | TM-type tRNase Z^S^ | clementine0.9_014182m.g | Phytozome | 355 |
| *Citrus clementina* (Ccl) | CclTRZ3 | tRNase Z^L^ | clementine0.9_001700m.g | Phytozome | 938 |
| *Citrus sinensis* (Csi) | CsiTRZ1 | TM-type tRNase Z^S^ | orange1.1g023022m.g | Phytozome | 288 |
| *Citrus sinensis* (Csi) | CsiTRZ2 | TM-type tRNase Z^S^ | orange1.1g018507m.g | Phytozome | 355 |
| *Citrus sinensis* (Csi) | CsiTRZ3 | tRNase Z^L^ | orange1.1g002976m.g | Phytozome | 939* |
| *Cucumis sativus* (Csa) | CsaTRZ1 | TM-type tRNase Z^S^ | Cucsa.369470 | Phytozome | 301* |
| *Cucumis sativus* (Csa) | CsaTRZ2 | TM-type tRNase Z^S^ | Cucsa.185180 | Phytozome | 347 |
| *Cucumis sativus* (Csa) | CsaTRZ3 | tRNase Z^L^ | Cucsa.359360 | Phytozome | 977 |
| *Eucalyptus grandis* (Egr) | EgrTRZ1 | TM-type tRNase Z^S^ | Egrandis_v1_0.020509m.g | Phytozome | 309 |
| *Eucalyptus grandis* (Egr) | EgrTRZ2 | TM-type tRNase Z^S^ | Egrandis_v1_0.021229m.g | Phytozome | 357* |
| *Eucalyptus grandis* (Egr) | EgrTRZ3 | tRNase Z^L^ | Egrandis_v1_0.001658m.g | Phytozome | 971 |
| *Glycine max* (Gma) | GmaTRZ1 | TM-type tRNase Z^S^ | Glyma20g01480 | Phytozome | 279 |
| *Glycine max* (Gma) | GmaTRZ2 | TM-type tRNase Z^S^ | ACU18735.1 | NCBI | 353 |
| *Glycine max* (Gma) | GmaTRZ3 | tRNase Z^L^ | Glyma13g43270 | Phytozome | 923* |
| *Glycine max* (Gma) | GmaTRZ4 | tRNase Z^L^ | Glyma15g02070 | Phytozome | 931* |
| *Manihot esculenta* (Mes) | MesTRZ1 | TM-type tRNase Z^S^ | cassava4.1_012878m.g | Phytozome | 297 |
| *Manihot esculenta* (Mes) | MesTRZ2 | TM-type tRNase Z^S^ | cassava4.1_013366m.g | Phytozome | ? |
| *Manihot esculenta* (Mes) | MesTRZ3 | tRNase Z^L^ | cassava4.1_001149m.g | Phytozome | 944 |
| *Manihot esculenta* (Mes) | MesTRZ4 | tRNase Z^L^ | cassava4.1_001661m.g | Phytozome | 923* |
| *Medicago truncatula* (Mtr) | MtrTRZ1 | TM-type tRNase Z^S^ | Medtr5g101320 | Phytozome | 323* |
| *Medicago truncatula* (Mtr) | MtrTRZ2 | TM-type tRNase Z^S^ | Medtr5g092020 | Phytozome | 345* |
| *Medicago truncatula* (Mtr) | MtrTRZ3 | tRNase Z^L^ | Medtr2g122460 | Phytozome | 950* |
| *Mimulus guttatus* (Mgu) | MguTRZ1 | TM-type tRNase Z^S^ | mgv1a010642m.g | Phytozome | 306 |
| *Mimulus guttatus* (Mgu) | MguTRZ2 | TM-type tRNase Z^S^ | mgv1a024577m.g | Phytozome | 364* |
| *Mimulus guttatus* (Mgu) | MguTRZ3 | tRNase Z^L^ | mgv1a000815m.g | Phytozome | 976 |
| *Populus trichocarpa* (Ptr) | PtrTRZ1 | TM-type tRNase Z^S^ | POPTR_0015s07570 | Phytozome | 301 |
| *Populus trichocarpa* (Ptr) | PtrTRZ2 | TM-type tRNase Z^S^ | POPTR_0014s15880 | Phytozome | 366 |
| *Populus trichocarpa* (Ptr) | PtrTRZ3 | tRNase Z^L^ | POPTR_0001s18750 | Phytozome | 941* |
| *Populus trichocarpa* (Ptr) | PtrTRL1 | tRNase Z^L^-like | POPTR_0001s25430 | Phytozome | ? |
| *Prumus persica* (Ppe) | PpeTRZ1 | TM-type tRNase Z^S^ | ppa009179m.g | Phytozome | 303 |
| *Prumus persica* (Ppe) | PpeTRZ2 | TM-type tRNase Z^S^ | ppa011171m.g | Phytozome | 330* |
| *Prumus persica* (Ppe) | PpeTRZ3 | tRNase Z^L^ | ppa000849m.g | Phytozome | 982 |
| *Ricinus communis* (Rco) | RcoTRZ1 | TM-type tRNase Z^S^ | 30147.t000355 | Phytozome | 300* |
| *Ricinus communis* (Rco) | RcoTRZ2 | TM-type tRNase Z^S^ | 29620.t000010 | Phytozome | 361 |
| *Ricinus communis* (Rco) | RcoTRZ3 | tRNase Z^L^ | 30146.t000117 | Phytozome | ? |
| *Vitis vinifera* (Vvi) | VviTRZ1 | TM-type tRNase Z^S^ | XP_002273058.1 | NCBI | 290 |
| *Vitis vinifera* (Vvi) | VviTRZ2 | TM-type tRNase Z^S^ | XP_002277241.1 | NCBI | 354 |
| *Vitis vinifera* (Vvi) | VviTRZ3 | tRNase Z^L^ | XP_002278956.1 | NCBI | 951 |
| **MONOCOTS** |  |  |  |  |  |
| *Brachypodium distachyon* (Bdi) | BdiTRZ1 | TM-type tRNase Z^S^ | Bradi3g08030 | Phytozome | 306 |
| *Brachypodium distachyon* (Bdi) | BdiTRZ2 | TM-type tRNase Z^S^ | Bradi4g33440 | Phytozome | 359 |
| *Brachypodium distachyon* (Bdi) | BdiTRZ3 | tRNase Z^L^ | Bradi2g09010 | Phytozome | 931* |
| *Brachypodium distachyon* (Bdi) | BdiTRZ4 | tRNase Z^L^ | Bradi2g07850 | Phytozome | ? |
| *Oryza sativa japonica* (Osa) | OsaTRZ1 | TM-type tRNase Z^S^ | NP_001046280.1 | NCBI | 302 |
| *Oryza sativa japonica* (Osa) | OsaTRZ2 | TM-type tRNase Z^S^ | LOC_Os09g30466 | Phytozome | 365 |
| *Oryza sativa japonica* (Osa) | OsaTRZ3 | tRNase Z^L^ | B9EUI7 | UniProt | 964 |
| *Setaria italica* (Sit) | SitTRZ1 | TM-type tRNase Z^S^ | Si017923m.g | Phytozome | 304* |
| *Setaria italica* (Sit) | SitTRZ2 | TM-type tRNase Z^S^ | Si030288m.g | Phytozome | 357 |
| *Setaria italica* (Sit) | SitTRZ3 | TM-type tRNase Z^S^ | Si019811m.g | Phytozome | 312* |
| *Setaria italica* (Sit) | SitTRZ4 | TM-type tRNase Z^S^ | Si017880m.g | Phytozome | 309 |
| *Setaria italica* (Sit) | SitTRZ5 | tRNase Z^L^ | Si000227m.g | Phytozome | 921 |
| *Sorghum bicolor* (Sbi) | SbiTRZ1 | TM-type tRNase Z^S^ | Sb04g009740 | Phytozome | 308* |
| *Sorghum bicolor* (Sbi) | SbiTRZ2 | TM-type tRNase Z^S^ | Sb02g028070 | Phytozome | 3593* |
| *Sorghum bicolor* (Sbi) | SbiTRZ3 | TM-type tRNase Z^S^ | Sb04g007440 | Phytozome | 324* |
| *Sorghum bicolor* (Sbi) | SbiTRZ4 | tRNase Z^L^ | Sb03g000750 | Phytozome | 932 |
| *Zea mays* (Zma) | ZmaTRZ1 | TM-type tRNase Z^S^ | GRMZM2G147727 | Phytozome | 302 |
| *Zea mays* (Zma) | ZmaTRZ2 | TM-type tRNase Z^S^ | ACG47847.1 | NCBI | 357 |
| *Zea mays* (Zma) | ZmaTRZ3 | tRNase Z^L^ | GRMZM2G379286 | Phytozome | 930 |
| **MOSSES** |  |  |  |  |  |
| *Physcomitrella patens* (Ppa) | PpaTRZ1 | TM-type tRNase Z^S^ | XP_001785449.1 | NCBI | 295 |
| *Physcomitrella patens* (Ppa) | PpaTRZ2 | TM-type tRNase Z^S^ | Pp1s17_61V6 | Phytozome | 420 |
| *Physcomitrella patens* (Ppa) | PpaTRZ3 | Bacterial-type tRNase Z^S^ | Pp1s409_46V6 | Phytozome | 336* |
| *Physcomitrella patens* (Ppa) | PpaTRZ4 | tRNase Z^L^ | Pp1s126_39V6 | Phytozome | 984* |
| *Physcomitrella paten* (Ppa) | PpaTLP1 | tRNase Z^L^-like | Pp1s3_568V6 | Phytozome | 923* |
| **LYCOPHYTES** |  |  |  |  |  |
| *Selaginella moellendorffii* (Smo) | SmoTRZ1 | TM-type tRNase Z^S^ | 167717 | Phytozome | 299 |
| *Selaginella moellendorffii* (Smo) | SmoTRZ2 | tRNase Z^L^ | 111409 | Phytozome | 771* |
| *Selaginella moellendorffii* (Smo) | SmoTRZ3 | tRNase Z^L^ | 97243 | Phytozome | 819* |
| *Selaginella moellendorffii* (Smo) | SmoTLP1 | tRNase Z^S^-like | 404484 | Phytozome | 303 |
| *Selaginella moellendorffii* (Smo) | SmoTLP2 | tRNase-like | XP_002967989 | NCBI | 346 |
| *Selaginella moellendorffii* (Smo) | SmoTLP3 | tRNase Z^L^-like | 416540 | Phytozome | 731 |
| **GREEN ALGAE** |  |  |  |  |  |
| *Chlamydomonas reinhardtii* (Cre) | CreTRZ1 | TM-type tRNase Z^S^ | Cre10.g431400 | Phytozome | 335 |
| *Chlamydomonas reinhardtii* (Cre) | CreTRZ2 | tRNase Z^L^ | Cre01.g039900 | Phytozome | 880* |
| *Chlamydomonas reinhardtii* (Cre) | CreTLP1 | tRNase Z^S^-like | Cre12.g492250 | Phytozome | 425* |
| *Chlamydomonas reinhardtii* (Cre) | CreTLP2 | tRNase Z^S^ -like | Cre07.g321900 | Phytozome | 378 |
| *Chlamydomonas reinhardtii* (Cre) | CreTLP3 | tRNase Z^L^-like | Cre01.g068350 | Phytozome | 710* |
| *Chlamydomonas reinhardtii* (Cre) | CreTLP4 | tRNase Z^L^-like | Cre09.g394700 | Phytozome | 1073 |
| *Micromonas pusilla* (Mpu) | MpuTRZ1 | TM-type tRNase Z^S^ | 5692 | JGI | 383* |
| *Micromonas pusilla* (Mpu) | MpuTRZ2 | Bacterial-type tRNase Z^S^ | 18700 | JGI | 398* |
| *Micromonas pusilla* (Mpu) | MpuTRZ3 | tRNase Z^L^ | 54013 | JGI | 953* |
| *Micromonas pusilla* (Mpu) | MpuTLP1 | tRNase Z^L^-like | C1N4P0 | UniProt | 866 |
| *Ostreococcus lucimarinus* (Olu) | OluTRZ1 | TM-type tRNase Z^S^ | 26345 | JGI | 313 |
| *Ostreococcus lucimarinus* (Olu) | OluTRZ2 | Bacterial-type tRNase Z^S^ | XP_001416263 | NCBI | 369* |
| *Ostreococcus lucimarinus* (Olu) | OluTRZ3 | tRNase Z^L^ | A4S9E8 | UniProt | 781* |
| *Ostreococcus lucimarinus* (Olu) | OluTLP1 | tRNase Z^L^-like | A4RR41 | UniProt | 780 |
| *Volvox carteri* (Vca) | VcaTRZ1 | TM-type tRNase Z ^S^ | 80918 | Phytozome | 308* |
| *Volvox carteri* (Vca) | VcaTRZ2 | tRNase Z^L^ | 118548 | Phytozome | 881* |
| *Volvox carteri* (Vca) | VcaTLP1 | tRNase Z^S^-like | 103834 | Phytozome | 445* |
| *Volvox carteri* (Vca) | VcaTLP2 | tRNase Z^L^-like | 100314 | Phytozome | 723* |
