# Supplementary material for: A survey of green plant tRNA 3'-end processing enzyme tRNase Zs, homologs of the candidate prostate cancer susceptibility protein ELAC2
Source: BMC Evol Biol. 2011 Jul 23;11:219. doi: 10.1186/1471-2148-11-219 (PMC3161902; doi:10.1186/1471-2148-11-219)
Supplement: Additional file 2 — Pairwise sequence comparisons of S. bicolor and S. italica tRNase ZSs. The accession numbers for proteins are listed in Additional file 1. The pairwise percent identity (I) and percent similarity (S) between tRNase ZSs from S. bicolor and S. italica were calculated using the Clustal W program [68]. [file 1471-2148-11-219-S2.DOC]

Additional file 2: Pairwise sequence comparisons of *S. bicolor* and *S. italica* tRNase Z^S^s

|  | SbiTRZ2  I S | SbiTRZ3  I S | SitTRZ1  I S | SitTRZ2  I S | SitTRZ3  I S | SitTRZ4  I S |
| --- | --- | --- | --- | --- | --- | --- |
| SbiTRZ1 | 48 60 | 62 73 | 70 83 | 47 59 | 80 84 | 91 95 |
| SbiTRZ2 |  | 47 59 | 57 68 | 93 95 | 44 56 | 48 62 |
| SbiTRZ3 |  |  | 73 80 | 44 56 | 57 68 | 62 72 |
| SitTRZ1 |  |  |  | 48 59 | 64 75 | 72 83 |
| SitTRZ2 |  |  |  |  | 43 57 | 47 61 |
| SitTRZ3 |  |  |  |  |  | 78 82 |

The accession numbers for proteins are listed in Additional file 1. The pairwise percent identity (I) and percent similarity (S) between tRNase Z^S^s from *S. bicolor* and *S. italica* were calculated using the Clustal W program (68).
