# Supplementary material for: A survey of green plant tRNA 3'-end processing enzyme tRNase Zs, homologs of the candidate prostate cancer susceptibility protein ELAC2
Source: BMC Evol Biol. 2011 Jul 23;11:219. doi: 10.1186/1471-2148-11-219 (PMC3161902; doi:10.1186/1471-2148-11-219)
Supplement: Additional file 3 — Alignment of candidate tRNase ZSs from S. bicolor and S. italic. The accession numbers for the candidates are listed in Additional file 1. The annotation of the alignment is described in the legend to Figure 1. [file 1471-2148-11-219-S3.DOC]

Additional file 3: Alignment of candidate tRNase Z^S^s from *S. bicolor* and *S. italica*

**EGxS**

**SbiTRZ1 ( 1 ) --------------MAKSGESTVEVASSSSSP-LTPASASATRPK-------------------------------AKHRMEIEGYPVDGVS**

**SitTRZ4 ( 1 ) --------------MAKVSESAAEAASTAAAPPLTSASAPPSRPK-------------------------------AKHRLEIEGYPVEGLS**

**SbiTRZ3 ( 1 ) --------------MTKGGQSASSSGPEDESAAATSTSAPTPAPGR--------------------------RPRATQQRLEIEGYHVEGIS**

**SitTRZ1 ( 1 ) --------------MAKG----SKSAAAADESAVISTSAPTRP--------------------------------RAMQRVEIEGYSVEGIS**

**SbiTRZ2 ( 1 ) MATASLFSLPSLRALSRTSARCSRFQTLAARKPVESSSSTATSGSRRSGGKGGGLLSVLDRALADEEEYRRARAQVQRKGVEVEGYAVEGIS**

**SitTRZ2 ( 1 ) MATASLFSPPSLRLLSRTTARLSRFQTLAARKPPASTVSGGGGGG--GGGKGGGLLSVLDRALADEEEYRRARAQVQRKGVEVEGYAIEGIS**

**SitTRZ3 ( 1 ) --------------MAKIGEFAAEKAHTPTHP-GTPASAPATRSNN------------------------------AKHLLEIEGYPVEGVS**

**xxG Motif I Motif II**

**SbiTRZ1 ( 47) IGGQETCVIFPTLSLAFDIGRCPQRAVSQEFLFVSHGHLDHIGGLPMYVATRGLFRLRPPTIFVPACLRELVERLFEVHRAIDQSELNHNLV**

**SitTRZ4 ( 48) IGGQETCVIFPTLSLAFDIGRCPQRAISQEFLFISHGHLDHIGGLPMYVATRGLFRLRPATIFVPACLRDLVERLFEVHRAIDQSELKHNLV**

**SbiTRZ3 ( 53) IAGHETCVMFPSLNLAFDIGRCPPLAVSQDFLFVSHAHMDHIGGLPVYVATRGRRRMRPPTVFVPACLADLVRRLFDVHRAMDQSDLDHKLV**

**SitTRZ1 ( 43) IAGHETCVMFPSLNLAFDIGRCPPFAVSQDLLFISHAHMDHIGGLPLYVATRGRRRMRPPTVFIPACLADLVRKLFEVHRAMDQSDLDHKLV**

**SbiTRZ2 ( 93) VGGHETCVTVPSLNVAFDIGRGPQFAVSQDYLFITHAHLDHIGGLPMYIATRGLYNLKPPTVFVPPCIKDDVEDLLQVHRRMSQIELKVELV**

**SitTRZ2 ( 91) VGGHETCVTVPSLNVAFDIGRGPPFAVSQDYLFITHAHLDHIGGLPMYIATRGLYNLKPPTVFVPPCIKDDVEELLQVHRRMSQIELSVELV**

**SitTRZ3 ( 48) VGGKETCVIFPTLSLAFDIGMCPQQAISQEFLFVSHGHLDHIGGLHIYVAARAFLGLRPPTIFVPACLQDHVARLFEVYHAIAHSELNYNLV**

**Motif III KL motif Motif IV**

**SbiTRZ1 (139) PLEVGEEYEFRRDLKVRAFRTYHTIPSQGYVIYSVKQKLKQEFIGLPGSEIKHLKLSGVEITNTVSTPEIAFTGDTTADFILDPDN-ADVLQ**

**SitTRZ4 (140) PLEVGEEYELRRDIKVRAFRTYHAIPSQGYVIYSVKQKLKQEFIGLPGSEIKRLKLSGVEITNTVSTPEIAFTGDTTSDFILDPDN-ADVLG**

**SbiTRZ3 (145) PLEVGEEYQLTKDLSVRPFRTYHVIPSQGYVIYKVKQKLKEEYAGLPGKELSNLKKSGVEITNVESTPEIAFSGDTMSDFILDPDN-ADVLK**

**SitTRZ1 (135) PLEVGEEYELGKDLRVRPFKTYHVVPSQGYVIYRLKHKLKDEYAGLPGKELGTLRKSGVEITNTVSTPEIAFTGDTMSDFILDPDN-ADVLK**

**SbiTRZ2 (185) ALDLGETYEIRNDLVARPFQTYHAIPSQGYVIYSIRRKLKKQYAHLKGSQIMKLKQSGTEITDTILYPEVAFTGDTKSDFILDPRN-ADALR**

**SitTRZ2 (183) ALDLGETYEIRNDLVARPFQTYHAIPSQGYVIYSIRRKLKKQYAHLKGSQIMKLKQSGTEITDTILYPEVAFTGDTKSDFILDPRN-ADALR**

**SitTRZ3 (140) PLEVGEEYQLRTDLKVRAFRTCHVIPSQGYVIYSVNKKLKQEFIGLPGSEIKQLRLSGVEITNMVSTPEIAFTGDTTLDFILDPDNNADVLR**

**xExT HxH Motif V**

**SbiTRZ1 (230) AKILVVESTFLDDS-ISVEHAREYGHTHLYEIASQSDKLGNKAILLIHFSARYTTEEIDAAINRLPP------SFRSRVYALKEGF-----**

**SitTRZ4 (231) AKILVVESTFLDDS-ISVEHAREYGHTHLFEIASQSDKLGNKAILLIHFSARYTTEEIDAAINRLPP------SFRSRVYALKEGF-----**

**SbiTRZ3 (236) AKILVVESTFIDDS-KSIEDARERGHIHLSEIVSLSDKLKNKAILLNHFSLRYTAEILFSFLKKVISSTLVCLGQRIFIVTFEPIFNTFS-**

**SitTRZ1 (226) AKILVVESTYIDDS-KSIEDAREKGHTHLSEIASLSDKLENKAILLNHFSNRYTAEDIDVAINRLPP---PFRSR---VYALKEGF-----**

**SbiTRZ2 (276) AKVLITEATFLDDH-VDVEHAREHGHMHLSEIMEHSQWFRNETIVLTHFSNRYSLEDIHQAVSRLQP------KLNSKIVALTEGFKSEYR**

**SitTRZ2 (274) AKVLITEATFLDDH-VDVEHAREHGHMHLSEIMENSQWFRNETIVLTHFSNRYSLEDIRQAVSRLQP------KLHSKVVALTEGFKSEHR**

**SitTRZ3 (232) AKILVVESTFLDDESHSVEHARKYGHTHLSEIARQSDKLENKAILLFHFSARYTTEEIDAAINRLPP------YFRSRIYALKEGFE----**
