# Supplementary material for: A survey of green plant tRNA 3'-end processing enzyme tRNase Zs, homologs of the candidate prostate cancer susceptibility protein ELAC2
Source: BMC Evol Biol. 2011 Jul 23;11:219. doi: 10.1186/1471-2148-11-219 (PMC3161902; doi:10.1186/1471-2148-11-219)
Supplement: Additional file 4 — Alignment of candidate TM-type tRNase ZSs identified in green plants. The accession numbers for the candidates are listed in Additional file 1. Annotation is as described in the legend to Figure 1. [file 1471-2148-11-219-S4.DOC]

Additional file 4: Alignment of candidate TM-type tRNase Z^S^s identified in green plants

**AcoTRZ2 ( 1 ) --------------------------------------------------MKISLGISTSPILLPFSKPTLKIPHNRVC--------IPSNQRNQHQNQHQHTLFALK-------**

**AlyTRZ2 ( 1 ) ---------------------------------------------------MQLSSFPISPSKIFPFTKYHAPPVIIHQ--------LAAQIQSHRSNFVSPVKVSG--------**

**AthTRZ2 ( 1 ) --------------------------------------------------MQLSSSFPISPPKIFPSTKHHKPPVITHQ--------LAAQIQSNRRHFVSPVKVSG--------**

**PtrTRZ2 ( 1 ) -----------------------------------------------MQISIPLFSSPPNPKIPSLFPFQHLPSIFHGK--------GKPTTLTTRNFHFLPLKIKNKPF----I**

**CclTRZ2 ( 1 ) ----------------------------------------------------MQLSLPTSPSKLPTIFPF-----HPSS--------IPKTPQSPHHLSLQSHVGPLN------A**

**CsiTRZ2 ( 1 ) ----------------------------------------------------MQLSLPTSPSKLPTIFPF-----HPSS--------IPKTPQSPHHLSLQSHVGPLN------A**

**CpaTRZ2 ( 1 ) ----------------------------------------------------MLTSVPILPSKLQSVFPFGHHHYHSIS--------HKPTPIHQFSFLVQNHVNPVN------S**

**PpeTRZ2 ( 1 ) ----------------------------------------------------MQIFLTISPCKAPLILPFHPPISKTPK--------TQRTSLKTLS------------------**

**RcoTRZ2 ( 1 ) ----------------------------------------------------MQTSLPISHSKFPSIFPFNHPISHKPT--------TTRTTTEHQYPFQTHIKKPIGPT----S**

**EgrTRZ2 ( 1 ) ----------------------------------------------------MRISIPISPSKSPQIFPFHHHHRRRHQ--------IPDLRRRPPALSLP--ASAVN------P**

**CsaTRZ2 ( 1 ) ----------------------------------------------------MQISIPISPLRPPQVFPFHQPLLHTPK--------PPGVALQSHLN-------PVN------S**

**GmaTRZ2 ( 1 ) ----------------------------------------------------MQISLSDLAFKTPQLFPIHHPIFPPKP--------PLNHQVSTQSHVSNVLKGSS--------**

**MtrTRZ2 ( 1 ) ----------------------------------------------------MQIPLTNPTFKPPQIFPFHHTIPSPK---------------QQLSFPTNSLKGS---------**

**MguTRZ2 ( 1 ) -----------------------------------------------------MITLNPTTSNLLNLHPFHPTPATHKH--------HLPPQTRRSVIACVSDAVVAGGGGGGGG**

**VviTRZ2 ( 1 ) ----------------------------------------------------MQISLPFSTSKVPYLSPLPNPTPQPPL--------TIPKPHPKSYITAHSSRPKGS-------**

**BdiTRZ2 ( 1 ) -------------------------------------------------------MATISLFSLPPLRLTRGLLSPSSG--PASR--FQTLASKKAGAP--TVSGPGAGGG----**

**OsaTRZ2 ( 1 ) -------------------------------------------------------MAATSLLSLPSLRLTHRLLVPASSSAPASRSQFQTLAAKKAAVA--TGTGEGGGGGR---**

**SbiTRZ2 ( 1 ) -------------------------------------------------------MATASLFSLPSLRALSRTSARCSR--------FQTLAARKPVESSSSTATSGSRRSG---**

**ZmaTRZ2 ( 1 ) -------------------------------------------------------MVTASLFSLPSLRVLSRTSAHGPR--------FQILAARKPVES--STATSGSRRGG---**

**SitTRZ2 ( 1 ) -------------------------------------------------------MATASLFSPPSLRLLSRTTARLSR--------FQTLAARKPPAS--TVSGGGGGGGG---**

**CreTRZ1 ( 1 ) -------------------------------------------------------------------------------------------MDTLTRALKEQTNLAHSPDTRATD**

**VcaTRZ1 ( 1 ) -------------------------------------------------------------------------------------------------------------------**

**MpuTRZ1 ( 1 ) ------------------------------------------MRAAATTRAPFRLALQLAAGRSRAASASASASARYPSLATVEGRRHRGGVRDPRAAVAVDVDAASASASTVAA**

**OluTRZ1 ( 1 ) ---------------------------------------------------------------------------------------------------------MATARDRPAG**

**PpaTRZ1 ( 1 ) -------------------------------------------------------------------------------------------------------------------**

**PpaTRZ2 ( 1 ) MHSLPQWRTATVVCAALSSHMTSHVRVCSLARIAAREFVPNCASVCCFGRRLQFQGRGGGGGGGVTGCGAVDRFAPRASTEGVAAMASIATVQVETEPDSDVTFAVGCRVSSSSF**

**SmoTRZ1 ( 1 ) -------------------------------------------------------------------------------------------------------------------**

**BdiTRZ1 ( 1 ) ----------------------------------------------------------------------------------------------------------MAKSR----**

**OsaTRZ1 ( 1 ) ----------------------------------------------------------------------------------------------------------MANSG----**

**SbiTRZ1 ( 1 ) ----------------------------------------------------------------------------------------------------------MAKSG----**

**ZmaTRZ1 ( 1 ) ----------------------------------------------------------------------------------------------------------MAKSGG---**

**SitTRZ4 ( 1 ) ----------------------------------------------------------------------------------------------------------MAKVSE---**

**SitTRZ3 ( 1 ) ----------------------------------------------------------------------------------------------------------MAKIG----**

**SbiTRZ3 ( 1 ) ----------------------------------------------------------------------------------------------------------MTKGGQSAS**

**SitTRZ1 ( 1 ) ----------------------------------------------------------------------------------------------------------MAKG----S**

**CsaTRZ1 ( 1 ) -------------------------------------------------------------------------------------------------------------------**

**AlyTRZ1 ( 1 ) -------------------------------------------------------------------------------------------------------------------**

**AthTRZ1 ( 1 ) -------------------------------------------------------------------------------------------------------------------**

**PpeTRZ1 ( 1 ) -------------------------------------------------------------------------------------------------------------------**

**MesTRZ1 ( 1 ) -------------------------------------------------------------------------------------------------------------------**

**RcoTRZ1 ( 1 ) -------------------------------------------------------------------------------------------------------------------**

**PtrTRZ1 ( 1 ) -------------------------------------------------------------------------------------------------------------------**

**CclTRZ1 ( 1 ) -------------------------------------------------------------------------------------------------------------------**

**CsiTRZ1 ( 1 ) -------------------------------------------------------------------------------------------------------------------**

**CpaTRZ1 ( 1 ) -------------------------------------------------------------------------------------------------------------------**

**GmaTRZ1 ( 1 ) -------------------------------------------------------------------------------------------------------------------**

**MtrTRZ1 ( 1 ) -------------------------------------------------------------------------------------------------MDPTRAFVTSVNRVIVKG**

**MguTRZ1 ( 1 ) ---------------------------------------------------------------------------------------------------------------MASS**

**AcoTRZ1 ( 1 ) -------------------------------------------------------------------------------------------------------------------**

**EgrTRZ1 ( 1 ) ---------------------------------------------------------------------------------------------------------------METK**

**VviTRZ1 ( 1 ) -------------------------------------------------------------------------------------------------------------------**

**EGxSxxG Motif I Motif II**

**AcoTRZ2 ( 51) -GSSGFLSAVDRVIE-EEEYRKARAQINRKGVDLEGYLIEGISIGGHETCVIVPRLNAAFDIGRCPSRAIQQDFLFITHAHLDHIGGLPMYVATRGLYSLKPPTIFVPPCIKEDV**

**AlyTRZ2 ( 49) -----YFSSISRAIEEEEEYRKARAAVNRKGVELESYAIEGISVGGHETCVIVPELKCVFDIGRCPSRAIQQKFLFITHAHLDHIGGLPMYVASRGLYNLEPPKIFVPPSIKEDV**

**AthTRZ2 ( 50) -----YFSSISRAIEEEEEYRKARAAVNRKGVELESYAIEGISVGGHETCVIVPELKCVFDIGRCPSRAIQQKFLFITHAHLDHIGGLPMYVASRGLYNLEPPKIFVPPSIKEDV**

**PtrTRZ2 ( 57) VKASSFLSEIGRAIEEEEDYRKARAAVIRKGIDLGGYAIEGLSIGGQETCIIIPEFKCAFDIGRCPTRAIHQNFVFITHAHLDHIGGLPMYVASRGLYSLKPPTIFVPPCIKDDV**

**CclTRZ2 ( 45) LKSAGFLSSISRAIDEEEEYRKARAAVVRKGIDLEGYTIEGVSIGGHETCVIIPELKCAFDIGRCPTRAIQQNFVFITHGHLDHIGGLPMYVASRGLYNLKPPTIFVPPSIKEDV**

**CsiTRZ2 ( 45) LKSAGFLSSISRAIDEEEEYRKARAAVVRKGIDLEGYTIEGVSIGGHETCVIIPELKCAFDIGRCPTRAIQQNFVFITHGHLDHIGGLPMYVASRGLYNLKPPTIFVPPSIKEDV**

**CpaTRZ2 ( 50) LKGSGYLSSIGLAIEQEEEYRKARAAVTRKGVDLEGYFIEGVSVGGQETCVIIPELKCAFDIGRCPSRAIQQKFVFITHAHLDHIGGLPMYVASRGLYNLEPPKIFVPPCIKEDV**

**PpeTRZ2 ( 38) -----------------KPYRLARSQVLRKGVDLEGYSIEGLSIGGHETCIIIPEFKCAFDIGRCPSRAIHQNFVFITHAHLDHIGGLPMYVASRGLYNLKPPTVFVPPCIKEDV**

**RcoTRZ2 ( 52) LKSTGYLSAIGRVIEEEEEYRKARAAVIRKGIDLEGYSIEGLSIGGQETCLIIPEFKCAFDIGRCPSRAIHQNFVFITHAHLDHIGGLPMYVASRGLYNLKPPTVFVPPCIKEDV**

**EgrTRZ2 ( 48) LKSSGYLSTIHRAMEEEEEYRKARAEVARKGVDVEGFSIEGVSVGGHETCVIVPEFKCAFDIGRCPTRAIHQNFVFITHAHLDHIGGLPMYVAFRGLYNLKPPTVFVPPCIKEDV**

**CsaTRZ2 ( 43) FRDSGLLSTIG----VEEEYRRARSQVNRKGVDLEGYSIEGISVGGQETCVIIPEFKCAFDIGRCPSRAIQQNFVFITHAHLDHIGGLPMYVASRGLYSLSPPTVFLPASIKEDV**

**GmaTRZ2 ( 48) ----GYLSEISKVIDHEEQYRVARSQVSRKVLDLEGYSIEGLSVGGQETCIIIPEFKCSFDIGRCPSRAIQQNFLFITHAHLDHIGGLPMYVASRGLFNLKPPTVFVPPCIKEDV**

**MtrTRZ2 ( 40) ----GYLSEISKAIDYEEQYRVAKSQVQRKGLDLEGYSIEGVSIGGHETCIIIPEFKCAFDIGRCPTRAIHQNFVFITHAHLDHIGGLPMYIGSRGLYNLKPPTVFVPPCIKDDV**

**MguTRZ2 ( 55) GGGSGFLSAIGRAIE-EEEYRKARAEVNRKGVGLDGYSVEGLSIGGHETCVIVPELKTAFDIGRCPSRAVQQNFLFITHAHLDHIGGLPMYIATRGLYNLKPPTVFVPPCIKEDV**

**VviTRZ2 ( 49) ----GYLSLIGRAIEEEEEYRKARAEVARNGVDLEGYSIEGISIGGHETCVIIPELKSAFDIGRCPSRAIQQNFLFITHAHLDHIGGLPMYVATRGLYNLKPPTIFVPPCIKEDV**

**BdiTRZ2 ( 51) -GGRGLLSVLDRALTDEEEYRRARAQVQRKGVEVEGYAIEGISVGGHETCITVPSLNVAFDIGRGPLFAVRQDYLFITHAHLDHIGGLPMYIATRGLYNLKPPTVFVPPCIKDDV**

**OsaTRZ2 ( 56) GAGGGLLSVLDRAMADEEEYRRARAQVHRKGVEVEGYAIEGVSVGGHETCVTVPSLNVAFDIGRGPLFAVSQDYLFITHAHLDHIGGLPMYIATRGLYNLKPPVVFVPPCIKDDV**

**SbiTRZ2 ( 50) GKGGGLLSVLDRALADEEEYRRARAQVQRKGVEVEGYAVEGISVGGHETCVTVPSLNVAFDIGRGPQFAVSQDYLFITHAHLDHIGGLPMYIATRGLYNLKPPTVFVPPCIKDDV**

**ZmaTRZ2 ( 48) SKGAGLLSVLDRAMADEEEYRRARAQVQRKGVEVEGYAIEGISVGGHETCITVPSLNVAFDIGRGPQFAVSQDYLFITHAHLDHIGGLPMYIATRGLYNLKPPTVFVPPCIRDDV**

**SitTRZ2 ( 48) GKGGGLLSVLDRALADEEEYRRARAQVQRKGVEVEGYAIEGISVGGHETCVTVPSLNVAFDIGRGPPFAVSQDYLFITHAHLDHIGGLPMYIATRGLYNLKPPTVFVPPCIKDDV**

**CreTRZ1 ( 25) PPMKAASTTAATKGTTKEGATALMADTRQTKLAVSGWELEGISISGQETSIIVPRAKVTFDIGRCPQRACFQQTVLLSHTHLDHVGGLPFHVCTRNMLQLPPSKVVVPQGFSAGV**

**VcaTRZ1 ( 1 ) ---MSSAVAGASTAAPVLNPPAFPVDSRQNKIIVCGMELEGVSISGQETSIIFPRGKVTFDIGRCPQRACFQQTVLLSHAHLDHVGGLPFHVCTREMLSLPASRVVVPQGFAAGV**

**MpuTRZ1 ( 74) AEMPENDPAAVSAAAGRPSSSARGGKERHAVHTLAGFRVEGVSVGGQETCVILPQLKIAFDSGRCPQRAVYADTLCLSHTHMDHVGGAGFYIASRALVSLPPPSVLIPAERAAAF**

**OluTRZ1 ( 11) REGDGEGDGDGDDAAQKRTKN----RDRHAVHELAGYKIEGVSVGGRETSVVLPALGVAFDSGRCPQRCVYADVMCLSHTHMDHVGGCGMYIATRGLLSLTPPTVLLPSARREAF**

**PpaTRZ1 ( 1 ) ----------MKDTPSSRHEQLKLGRSKTGALEVDGLSIEGVSVGGQETCILIPALKLAFDIGRCPERAISQDFLFISHAHMDHIGGVCMYVATRGLFKMKPPTVIVPKCLKPTV**

**PpaTRZ2 (116) LLAPQDKPVRKKVAPGPGYGQTKLGRPTTGELEVGGFSIEGVSVGGQETCVMLPALKVAFDSGRCPGRIIDMDYVFITHAHMDHIGGLTMYIASRGLKKKKVPTVIVPKCIKATV**

**SmoTRZ1 ( 1 ) -------MEEVALEDAKLKSSVRGGRRDLASVNAGGFAVEGISIGGHETCVIVPSMKIAFDIGRCPQRAISQDFLFISHSHMDHIGGIAMYVATRGLYSMKPPTIFVPSSIKATV**

**BdiTRZ1 ( 6 ) ---KSAEAATSTAPPPPPPSSAAKPKAKTR-LEIEGYPVEGISIGGQETCVIFPTLSLAFDIGRCPQRAVSQDFLFISHAHLDHIGGLPMYVATRGLYRLRPPTIFVPKYLRELV**

**OsaTRZ1 ( 6 ) ---KSSPAATSTTAPPP-----GRPKAKAPPLTVEGYPVEGISIGGQETCVIFPTLSAAFDIGRCPQRAVSQEFLFISHAHLDHIGGLPMYVATRGLYRQRPPTIFIPACLRDPV**

**SbiTRZ1 ( 6 ) -ESTVEVASSSSSPLTPASASATRPK-AKHRMEIEGYPVDGVSIGGQETCVIFPTLSLAFDIGRCPQRAVSQEFLFVSHGHLDHIGGLPMYVATRGLFRLRPPTIFVPACLRELV**

**ZmaTRZ1 ( 7 ) -ESTVEVP-------IPASASATRPK-AKHRIEIEGYPVDGVSIGGQETCVIFPTLSLAFDIGRCPQRAVSQEFLFVSHGHLDHIGGLPMYVATRGLFRLRPPTIFVPACLRDLV**

**SitTRZ4 ( 7 ) -SAAEAASTAAAPPLTSASAPPSRPK-AKHRLEIEGYPVEGLSIGGQETCVIFPTLSLAFDIGRCPQRAISQEFLFISHGHLDHIGGLPMYVATRGLFRLRPATIFVPACLRDLV**

**SitTRZ3 ( 6 ) -EFAAEKAHTPTHPGTPASAPATRSNNAKHLLEIEGYPVEGVSVGGKETCVIFPTLSLAFDIGMCPQQAISQEFLFVSHGHLDHIGGLHIYVAARAFLGLRPPTIFVPACLQDHV**

**SbiTRZ3 ( 10) SSGPEDESAAATSTSAPTPAPGRRPRATQQRLEIEGYHVEGISIAGHETCVMFPSLNLAFDIGRCPPLAVSQDFLFVSHAHMDHIGGLPVYVATRGRRRMRPPTVFVPACLADLV**

**SitTRZ1 ( 6 ) KSAAAADESAVISTSAPTRP------RAMQRVEIEGYSVEGISIAGHETCVMFPSLNLAFDIGRCPPFAVSQDLLFISHAHMDHIGGLPLYVATRGRRRMRPPTVFIPACLADLV**

**CsaTRZ1 ( 1 ) --------MAAGTASFSSKNLDSNPMEMSKGIQIEGYPIKGLSIGGHETCIIFPTLNLAFDIGRCPQKAISQDFLFISHGHMDHIGGLPLYVATRGLYKMGPPTIIVPKSIKNDV**

**AlyTRZ1 ( 1 ) -------------------------MEKKKAMQIEGYPIEGLSIGGHETCIIFPSLRIAFDIGRCPHRAISQDFLFISHSHMDHIGGLPMYVATRGLYKMKPPTIIVPKSIKETV**

**AthTRZ1 ( 1 ) -------------------------MEKKKAMQIEGYPIEGLSIGGHETCIIFPSLRIAFDIGRCPHRAISQDFLFISHSHMDHIGGLPMYVATRGLYKMKPPTIIVPASIKETV**

**PpeTRZ1 ( 1 ) --MTGSRSRDSEQVTQKPNSDSDPASKKARGLTIEGYPVEGLSIGGHETCIIFPTLNLSFDIGRCPQRAISQDFLFISHAHMDHIGGLPMYVATRGLYSMKPPTIIVPTCIKEVV**

**MesTRZ1 ( 1 ) -----MSSKQ--QKGDPVSAPKDKRG-DRKIMTIEGYPVEGLSIGGHETCIIFPSLNLAFDIGRCPQRAISQDFLFISHAHMDHIGGLPMYVATRGLYRMKPPTIIVPTSIKETV**

**RcoTRZ1 ( 1 ) -----MSSNQQTPEADSVPNPNVKREKEKRVMTIEEYPIEGLSIGGHETCIILPSLNLAFDIGRCPQRAISQDFLFISHAHMDHIGGLPMYVATRGLYRMKPPTIIVPTSIKETV**

**PtrTRZ1 ( 1 ) ----MERRKERGEENESDDSVREKRKEEETKRIIEGYPVEGLSIGGHETCIIFSSLNMAFDIGRCPQRAISQDFLFISHAHMDHIGGLPMYVATRGLYRMKPPTVVVPTCIKETV**

**CclTRZ1 ( 1 ) ------MATKAKE-----EEKSGRG------MQLQGYKVEGLSIAGHETCIIFPSLDLAFDIGRCPSRALSQNFLFISHAHMDHIGGLPMYVATRGLYRMKPPTIIVPSCIKEDV**

**CsiTRZ1 ( 1 ) ------MATKAKE-----EEKSGRG------MQLQGYKVEGLSIAGHETCIIFPSLDLAFDIGRCPSRALSQNFLFISHAHMDHIGGLPMYVATRGLYRMKPPTIIVPSCIKEDV**

**CpaTRZ1 ( 1 ) ------MSNSGKEKQQGLESKSPKNN--KKRIEIQGYPVEGLSIAGHETCIIFPTLKLAFDIGKCPQRAISQDFLLISHGHMDHIGGLPMYVATRGLYGMKPPTIIVPTAIKKNV**

**GmaTRZ1 ( 1 ) --------------------------MTGKDLEIEGYTVGGLSIGGHETCVIFPTLKVAFDIGRCPPRAVSQNFLLITHAHMDHIGGLPMYVATRGLYRMKPPTIIVPISVKEDV**

**MtrTRZ1 ( 19) FSVLPQYHSIALMELGTEKSSETESKKKSKGLNIEGYQVEGLSIGGHETCVIFSNFRVAFDIGRCPPRAVSMDFLLISHAHMDHIGGLPMYVATRGLYRMKPPTIIVPISVKEDV**

**MguTRZ1 ( 5 ) DERAYESTNSESEK--PK-AKKKKKKASEEELRIEGYSIDGLSIAGHETCVILPTLNLAFDIGKCPQRAVSQQFLFISHGHMDHIGGLPMYVATRGLYRMAPPTIFVPKVIKENV**

**AcoTRZ1 ( 1 ) ---------------------METSKKK-ENLEIEGYPIEGLSISGHETCIIFPSLKIAFDIGKCPQRAIFQEFLFISHGHMDHIGGLPMYVATRGLYSLKPPTIIVPMEIKESV**

**EgrTRZ1 ( 5 ) PGSGKTEVGKESEQGPSESADSCTDRRSGKKLMIQGYPIEGLSIAGHETCVILPSLKLAFDIGRCPQRAISQDFLFISHAHMDHIGGVAMYVASRGLFSLKPPTVIVPKCVKEDV**

**VviTRZ1 ( 1 ) --------MKNSE------ANSIVTKKE-KGIQIEGYPVEGLSIGGQETCIILPSLKLAFDIGRCPQRAISQDFLFISHGHMDHIGGLPMYVATRGLYRMKPPIIIVPKAIKENV**

**Motif III KL Motif Motif IV**

**AcoTRZ2 (164) EKLFEVHRNMSQV-DLEFDLVPLDVGDTYELRNDLVVRSFKTHHVIPSQGYVVYSIRKKLKKQYAHLIMKGKQIEKLKKSGVEITDTILSPEMAFTGDTTSDFLLDPRN---ADA**

**AlyTRZ2 (159) EKLLEIHRTMGQV-ELNVELIPLDVGETYELRNDIVVRPFATHHVIPSQGYVIYSVRKKLQKQYAHL--KGKQIEKIKKSGVEITDTILSPEIAFTGDTTAEYMLDPRN---ADA**

**AthTRZ2 (160) EKLLEIHRTMGQV-ELNVELIPLAVGETYELRNDIVVRPFATHHVIPSQGYVIYSVRKKLQKQYAHL--KGKQIEKIKKSGVEITDTILSPEIAFTGDTTSEYMLDPRN---ADA**

**PtrTRZ2 (172) EKLFDIHRAMGQV-ELNFDLVALDVGETYELRNDVVVRPFRTQHVIPSQGYVIYSVRKKLKKQYIHL--KGKQIEKLKKSGVEITDIILSPEVAFTGDTTAEYMLDPRN---ADA**

**CclTRZ2 (160) EKLFEIHRSLGNV-ELNLDLVALDVGETYEMRNDIVVRPFKTHHVIPSQGYVIYLLRKKLKKQYIHL--KGKQIEKLKKSGVEITDIILSPEVAFTGDTTSEFMLNPRN---ADA**

**CsiTRZ2 (160) EKLFEIHRSLGNV-ELNLDLVALDVGETYEMRNDIVVRPFKTHHVIPSQGYVIYLLRKKLKKQYIHL--KGKQIEKLKKSGVEITDIILSPEVAFTGDTTSEFMLNPRN---ADA**

**CpaTRZ2 (165) EKLFEIHRAMGNV-ELNLDLVALDIGETYELRNDIVVRPFRTHHVIPSQGYVIYSIRKKLKKQYSHL--KGKQIEKLKKSGVEITDIILSPEVAFTGDTTSEYMLDPRN---ADA**

**PpeTRZ2 (136) EKLIDIHQTMGHV-ELNLDLVALDVGETYEMRNNLVVRPFRTDHAIPSQGYVIYSVRKKLKKQYMHL--KGKQIEKLKKSGVEITDVILSPEVAFTGDTTSDYMLDPRN---ADA**

**RcoTRZ2 (167) EKLFDIHRSMGQV-ELNLELVALDVGETYELRNNVVVRPFRTQHVIPSQGYVIYSVRKKLKKQYTHL--KGKQIEKLKKSGVEITDIMLSPEVAFTGDTTAEYMLDPHN---ADA**

**EgrTRZ2 (163) EKLLDIHRTMSQV-ELNLDLVALDVGETYELRNNLVVRPFKTNHVLPSQGYVIYSVRKKLKKRYIHL--KGNRIENLKKSGVEITDTILSPEVAFTGDTTSDYMLNPRS---ADA**

**CsaTRZ2 (154) EKLLEIHRNMGQV-ELDVDLVALDVGETYEMRNNLVCRAFETHHVIPSQGYVIYSVRKKLKKQYMHL--KGKQIEKLKKSGVEITDTILSPEVAFTGDTTPDFMLDPRN---ADA**

**GmaTRZ2 (159) EKLLDIHRTMGQV-ELNAEVVALDVGETYEIRNNLVVRPFKTQHVIPSQGYVVYSIRKKLRKQYAHL--NGKQIEKLKKSGVEITDMMLSPEVAFTGDTTSDFMLDPCN---ADA**

**MtrTRZ2 (151) QKLLDVHKTLGQV-ELNCELVALDVGETYEIRNDLVVRPFRTQHVIPSQGYIVYSIRKKLRKQYSHL--NGKQIEKLKKSGVEITDTILSPEVAFTGDTTSDFMLDPLN---ADA**

**MguTRZ2 (169) ERLIDIHRVMGRV-ELNVDLVPLDVGETYEMRNDIVVRPFETHHVIPSQGYVIYSVRKKLRKQYTHL--KGKQIEKLKKSGVEITDTILSPEVAFTGDTTSDFFLDPRS---ADA**

**VviTRZ2 (160) EKLFDIHRALSQV-ELKLDLVALDVGETYEMRNNLVVRPFKTHHVIPSQGYVIYTVRKKLKKQYIHL--KGKQIEKLKNSGIEITDTILSPEVAFTGDTRSDFMLEPRN---ADA**

**BdiTRZ2 (165) EELLQIHRRMSRI-ELEVELVALDLGETYELRNDLVARPFQTHHTVPSQGYVIYSVRRKLKKQYAHL--KGTQIVKLKQSGSEITDTILYPEVAFTGDTKSDFILDPRN---ADA**

**OsaTRZ2 (171) EDLLQIHRRMSQV-DLKVELVALDLGETFEIRNDLVARPFETHHAIPSQGYVIYSVRRKLKKQYAHL--KGNQIMKMKQSGAEITDTILYPEVAFTGDTKSDFILDPRN---ADA**

**SbiTRZ2 (165) EDLLQVHRRMSQI-ELKVELVALDLGETYEIRNDLVARPFQTYHAIPSQGYVIYSIRRKLKKQYAHL--KGSQIMKLKQSGTEITDTILYPEVAFTGDTKSDFILDPRN---ADA**

**ZmaTRZ2 (163) EDLLQVHRRTSQI-ELKVELVALDLGETYEIRNDLVARPFQTYHTIPSQGYVIYSIRRKLKKQYAHL--KGSQIMKLKQTGTEITDTILYPEVAFTGDTKSDFILDPRN---ADA**

**SitTRZ2 (163) EELLQVHRRMSQI-ELSVELVALDLGETYEIRNDLVARPFQTYHAIPSQGYVIYSIRRKLKKQYAHL--KGSQIMKLKQSGTEITDTILYPEVAFTGDTKSDFILDPRN---ADA**

**CreTRZ1 (140) RKLMDAVLELQSSPPIDYEVLELEAGQDFELPSGFLCRTFPTTHAIPSQGYVLYSQRKKLKAELQGK--SQAEIKELRFSGVDVSETHQVPEIAFTGDTTSAFLDAETNATLEDA**

**VcaTRZ1 (113) RKLVDAARELQGSPPLDYQVLELQLGEDHVLPSGYLCRCFPTTHTITSQGYVLYSQRRKLKAELQGK--SQEEIRQLRLAGQDVTDTVAVPEIAFTGDTTAGFLDGPGCATLEDA**

**MpuTRZ1 (189) DAFIRSLRELDAS-ELPHHLVPISPGETHVVSKLHVVRPFQTIHPVASQGYVVYGTKEKLKAEHAGK--TGAQIKALRDAGAKVTDTIEVPEVAFTGDTSAEWIRRATA---TDA**

**OluTRZ1 (122) GTFIESLRALDDS-ELNHRAIGIDPGERYAMNKLFEIAAFRTRHPVPSQGYVVYGTKQKLKPAYAGL--SGPEIKRLRDDGEQVTDKVEVPEVAFTGDTTGDWIDDPAN---ADA**

**PpaTRZ1 (106) EKLFDVHRELDGS-ELKHQLIGLDIGEEFNMGKNLVVKAFKTYHVVPSQGYVVYSVKNKLKPEYLGL--PGQKIKELKMSGVQITDTLRVPEVAFTGDTTPDFILDDAN---IDA**

**PpaTRZ2 (231) EKLLAVQRELDES-PLPVNLIGMDIGEEFDLGKGLIVKAFKTYHVVPSQGYVIYTVKQKLKAEHVGL--PGKEIKALRESGVEVSETVRISEVAFTGDTTSGFFLDEAN---EDV**

**SmoTRZ1 (109) EKLFDVYRELDQA-ELSMKLVGLDIGEEYDLGKGYIVKPFKTYHVIPSQGYIIYAVKNKLKPEYVGL--PGDKIKSLRFSGVEVTNVTRSPEVAFTGDTTVDFIGDENN---ADV**

**BdiTRZ1 (117) ERLFDVHRAMDQS-ELKHTLVPLEVGEEYELRRDLKVKAFKTYHVIPSQGYVIYSVKQKLKQEFLGL--PGSEIKRLKLSDVEITNTVTVPEIAFTGDTMSDFILDPDN---ADV**

**OsaTRZ1 (113) ERLFELHRSMDQS-ELSHNLVPLEIGQEHELRRDLKVKAFKTYHAIPSQGYVIYTVKQKLKPEYLGL--PGSEIKQLKLSGVEITNTLTVPEIAFTGDTMADFILDPDN---ADV**

**SbiTRZ1 (119) ERLFEVHRAIDQS-ELNHNLVPLEVGEEYEFRRDLKVRAFRTYHTIPSQGYVIYSVKQKLKQEFIGL--PGSEIKHLKLSGVEITNTVSTPEIAFTGDTTADFILDPDN---ADV**

**ZmaTRZ1 (113) ERLFEVHRAMDQS-ELNHNLVPLEVGEEYELRRDLKVRAFRTYHAIPSQGYVIYSVKQKLKQEFIGL--PGSEIKRLKLSGVEITNTVSTPEIAFSGDTTADFILDPDN---ADV**

**SitTRZ4 (120) ERLFEVHRAIDQS-ELKHNLVPLEVGEEYELRRDIKVRAFRTYHAIPSQGYVIYSVKQKLKQEFIGL--PGSEIKRLKLSGVEITNTVSTPEIAFTGDTTSDFILDPDN---ADV**

**SitTRZ3 (120) ARLFEVYHAIAHS-ELNYNLVPLEVGEEYQLRTDLKVRAFRTCHVIPSQGYVIYSVNKKLKQEFIGL--PGSEIKQLRLSGVEITNMVSTPEIAFTGDTTLDFILDPDNN--ADV**

**SbiTRZ3 (125) RRLFDVHRAMDQS-DLDHKLVPLEVGEEYQLTKDLSVRPFRTYHVIPSQGYVIYKVKQKLKEEYAGL--PGKELSNLKKSGVEITNVESTPEIAFSGDTMSDFILDPDN---ADV**

**SitTRZ1 (115) RKLFEVHRAMDQS-DLDHKLVPLEVGEEYELGKDLRVRPFKTYHVVPSQGYVIYRLKHKLKDEYAGL--PGKELGTLRKSGVEITNTVSTPEIAFTGDTMSDFILDPDN---ADV**

**CsaTRZ1 (108) ENLLEVHRRLDQS-ELRCNLIGLEVGEELSLRRDLKVRVFRTYHVIDSQGYLLYSVKQKLKKDYLGL--SGNEIKKLRLSGVEITYTITEPEVAFTGDTTSDFIVDENN---IDV**

**AlyTRZ1 ( 91) ESLFEVHRKLDSS-ELKHNLVGLDIGEEFIIRKDLKVKAFKTYHVIQSQGYVVYSTKHKLKQEYIGL--SGNEIKNLKASGVEITDSITTPEVAFTGDTTADFVVDESN---ADA**

**AthTRZ1 ( 91) ESLFEVHRKLDSS-ELKHNLVGLDIGEEFIIRKDLKVKAFKTFHVIQSQGYVVYSTKYKLKKEYIGL--SGNEIKNLKVSGVEITDSIITPEVAFTGDTTSDFVVDETN---ADA**

**PpeTRZ1 (114) EQLFEVHRKMDQS-ELKHNLIGLDVGEEVYLRKDLKVKAFKTYHVIPSQGYVVYSIKQKLKQEYVGL--SGNEIKNLKSSGVEITNTSTEPQVAFTGDTMSDFITDSSN---FDV**

**MesTRZ1 (108) EELFEVHRKLDGS-ELNHNLIGLDVGEEFYMRRDLKVRAFRTYHAIQSQGYVVYSMKQKLKQEYLGL--SGNEIKNLKASGVEITNTITSPEIAFTGDTMSDFIIDETN---SDA**

**RcoTRZ1 (111) EQLFEVHRKLDGS-ELNHKLIGLDVGEEFYVRKDLKVRAFRTYHAIQSQGYVVYSIKHKLKQEFLGL--SGNEIKNLKASGVEITNTVTAPEIAFTGDTMSDFIIDETN---IDA**

**PtrTRZ1 (112) EQLFEVHRRLDGS-ELKHHLIALDVGQEFYVRKDLKVRAFKTYHAIQSQGYVVYSVKQKLKQEYLGL--SGNEIKSLKSSGVEITNTVTSPEIAFTGDTMSDFIIDETN---IDV**

**CclTRZ1 ( 99) EQLFEIHRRMDHS-ELNHTLVGLDVGEEFCMRKDLFVKAFKTYHVIQSQGYVVYSVKQKLKQEYLGL--PGDEIKKLKSSGTEITYTVTTPEVAFTGDTMSDFIVDEAN---IDV**

**CsiTRZ1 ( 99) EQLFEVHRRMDHS-ELNHTLVGLDVGEEFCMRKDLFVKAFKTYHVIQSQGYVVYSVKQKLKQEYLGL--PGDEIKKLKSSGTEITYTVTTPEVAFTGDTMSDFIVDEAN---IDV**

**CpaTRZ1 (108) EQLFEVHRKMDHS-ELKHNLVGLDVGEEFYLRRDLKVRAFRTYHVIQSQGYVVYSIKQKLKHEYLGL--SANELKNLKSSGEEITNTVTAPEVAFTGDTTSDFIVDEAN---ADV**

**GmaTRZ1 (90) EKLFEIHRKMDQS-ELKHNLIGLDVGEEFYLRKDLKVKAFRTYHVIPSQGYILYSEKQKLKPEYVGL--SGNEIKNLKSSGVEITYTLTEPEIAFTGDTMSDFIVDENN---TDV**

**MtrTRZ1 (134) EKLFEIHRKMDQS-ELKHNLIGLDVGEEFSLRQDLKVKAFRTYHVIPSQGYILYSVKNKLKPEYIGL--SGDEIKKLKFSGVEITNTLKEPEIAFTGDTMSDFIIDENN---TDV**

**MguTRZ1 (117) EKIFEAHRAMDHS-ELNHTLIGLDVGEEFYLRRDLKVRAFRTYHVIPSQGYIVYSVRQKLKQEYVGL--TGAEIKNLKQSGVEITDTYTSPEVAFTGDTMSDFIVDPEN---IDA**

**AcoTRZ1 (94) EKLLQVHRDMDQS-ELKCNIIGLNIGEEYQLRKDLVVKAFRTYHVIPSQGYIVYCVKHKLKQEYVGL--PGNEIKKLKLSGVEITETSTSPEIAFTGDTMSDFIVDPNN---VDA**

**EgrTRZ1 (120) ERLFDVHRKMDNS-ELKHNLIGMDVGEEIYIRRDVKVRAFRTYHVIPSQGYVVYSVKEKLKQDYMGL--PGNKIRDLKLSGVEITYTTTTPEVAFTGDTTSDFIHDENN---ADV**

**VviTRZ1 (101) EMLFQVHRAMDES-ELKHNLIGLNVGEEFYLRKDLKVRAFRTYHVIPSQGYVVYSVKQKLKQEYVGL--PGNEIKNLKLSGVEITYTMTTPEIAFTGDTMSDFILDHTN---VDA**

**xExT HxH Motif V**

**AcoTrz2 (275) LRAKVLVTEATFLDGG-ISIEHARQHGHTHLFELMEHA--EWLRNKAILLTHFSSRYKKEDIDEAVLK----LQAKVSGKVVALTEGFK--------**

**AlyTrz2 (268) LRSKVLITEATFLDES-FSTEHAQALGHTHISQIIENA--KWIRSKTVLLTHFSSRYHVEEIREAVLK----LQSKVSAKVIPLTEGFRSRYS----**

**AthTrz2 (269) LRAKVLITEATFLDES-FSTEHAQALGHTHISQIIENA--KWIRSKTVLLTHFSSRYHVEEIREAVLK----LQSKVSAKVIPLTEGFRSRYS----**

**PtrTrz2 (281) LRAKVLITEATFLDED-FTTEHARQRGHTHLFEIIENA--KWIRSKAILLTHFSSRYSIEDIREAVSK----LQSRVSAKVVPLTEGFKSMYS----**

**CclTrz2 (269) LRAKILITEATFLDDE-MSIEHAQQHGHTHLSEIIENA--KWIRNKAVLLTHFSSRYHIEDIRQAVLK----LQSKVSAKVVPLTEGFKSVYTS---**

**CsiTrz2 (269) LRAKILITEATFLDDE-MSIEHAQQHGHTHLSEIIENA--KWIRNKAVLLTHFSSRYHIEDIRQAVLK----LQSKVSAKVVPLTEGFKSVYTS---**

**CpaTrz2 (274) LRAKILITEATFLDEG-FSIDHARQHGHTHLFEIIENA--QWIRNKAVLLTHFSSRYNIEDIRQAALM----LQSKVSAKVVPLTEGFKSVYS----**

**PpeTrz2 (245) LRAKVLITEATFLDEG-CSIEHARQHGHTHIDEIIENA--QWIRNKSVLLTHFSSRYHLEDIRQAVSK----LQSKLSAKVVPLTEGFKSMHA----**

**RcoTrz2 (276) LRAKVLITEATFLDEG-FSIEHARQHGHTHLLEIIENA--EWIRNKSVLLTHFSSRYTVEDIRQAVLK----LQSKVSAKVVPLTEGFKSTYS----**

**EgrTrz2 (272) LRAKVLITEATFLDDN-ISIEHAQQHGHTHLFEIIEHA--QWIRSKAILLTHFSSRYNVEDIRQAASK----LQSKVSAKVVTLTEGFKSAYS----**

**CsaTrz2 (263) LRAKILITEATFLDEA-VSIEHARQHGHTHIFEIIENA--QWIRNKAILLTHFSSRYHIEDIRKAVSK----LQSNLTAKVVPLTEGFKSEY-----**

**GmaTrz2 (268) LRAKIFITEATFLDDS-FSIDHARQHGHTHLFEIIANA--QWIRNEAVLLTHFSPRYTIEDIRQAASK----LQSRLSAKVVPLTEGFKSMYS----**

**MtrTrz2 (260) LRAKVLITEATFLDDS-TTVEHARQHGHTHISELIENA--QWIRNKTVLLTHFSSRYNIEDIRQAASE----LQSKTSAKVVPLTEGFKSQYT----**

**MguTrz2 (278) LRAKILITEATFLDES-VSVEHAREHGHTHLSEIMEHA--QWIRNQTVVLTHFSSRYHIEDIRQAVSK----LQSKVSAKVIGLTEGFKSLYNT---**

**VviTrz2 (269) LRAKVLITEATFLDNG-ISIDHAREHGHTHLFEIIENA--EWIRNKAVLLTHFSSRYHIEDIRKAVSK----LQSKVSARVVPLMEGFKSMYA----**

**BdiTrz2 (274) LRAKVLITEATFLDDQ-VDVDHAREHGHMHLSEIMEHS--QWFRNETVILTHFSNRYSLEDIRQAVSR----IQPKLLSKVVALTEGFKSEYS----**

**OsaTrz2 (280) LRAKVLITEATFLDDQ-IDVDHAREHGHMHLSEIMEHS--QWFRNKAIVLTHFSNRYSLEDIRQAVSK----LQSKLSSKVVALTEGFKSDYR----**

**SbiTrz2 (274) LRAKVLITEATFLDDH-VDVEHAREHGHMHLSEIMEHS--QWFRNETIVLTHFSNRYSLEDIHQAVSR----LQPKLNSKIVALTEGFKSEYR----**

**ZmaTrz2 (272) LRAKVLITEATFLDDH-VDVEHAREHGHMHLSEIMEHF--QWFRNETIVLTHFSNRYSLEDIRQAVSR----LQPKLNSKVVALTEGFKSDYR----**

**SitTrz2 (272) LRAKVLITEATFLDDH-VDVEHAREHGHMHLSEIMENS--QWFRNETIVLTHFSNRYSLEDIRQAVSR----LQPKLHSKVVALTEGFKSEHR----**

**CreTrz1 (253) LKAKVLCIEMTFISED-VTVEEARGKGHMHITDFVAHA--HRFQNETIVLIHFSPRYKRSEILHQLDI---MLPPALRAKCVPLLNGIE--------**

**VcaTrz1 (226) LKARVLIMEMTFLCDD-VTVDEARDKGHMHIADFVANA--HRFQNEAIVLIHFSPRYKRTDIITTLNT---MLPPSLMAKCVPLLNGIE--------**

**MpuTrz1 (298) LRAKLLICECTFVDDA-VSPEGARDFGHTHIDELAAAARDGKFQNEAVLLIHFSARYRGREIEDAIAE---KFPEEFAKRVTPLLVGFAP-------**

**OluTrz1 (231) LRAKLLIMECTFIDDA-VSKHDAERFGHTHIDDIVARA--DKFQNEAILLIHFSARYKAEEVRAALKA---KLPRALYEKCTPMLVGFD--------**

**PpaTRZ1 (215) LQAKLLIMETTFLDDA-VTIQHAREYGHTHLSEVIKYA--NRLLNKSILFIHFSARYKREEILRAVEE----LPPPLQGRVAALTEGF----------**

**PpaTrz2 (340) LHAKLLIMESTFLDDS-TSVEDANKYGHMHLFEVLAHA--DKFKNKDILFIHFSARYQQEDICKAIEN----IPAPLQGRVHALTEGF----------**

**SmoTrz1 (218) LRAKLLIMEATFINDS-GTVEHAREYGHSHLSEIVALS--DKFQNKAILLIHFSARYSKQEIIEAIEK----LPETLKSRTYALTEGFF--------**

**BdiTrz1 (226) LKAKILVVESTFLDDS-VSVEHAREYGHTHLFEIASQS--DKLQNRAILLIHFSARYTTEEIDAAISR----LPPSFRSRVYALKEGI----------**

**OsaTrz1 (222) LKAKILVVESTFVDDS-VTIEHAREYGHTHLFEILNQC--DKLENKAILLIHFSARYTAEEIDIAINK----LPPSFRSRVHALKEGF----------**

**SbiTrz1 (228) LQAKILVVESTFLDDS-ISVEHAREYGHTHLYEIASQS--DKLGNKAILLIHFSARYTTEEIDAAINR----LPPSFRSRVYALKEGF----------**

**ZmaTrz1 (222) LQAKILVVESTFLDDS-ISVEHAREYGHTHLFEITSQA--DKLQNKAILLIHFSARYTTEEIDAAINK----LPPSFRSRVYALKEGF----------**

**SitTrz4 (229) LGAKILVVESTFLDDS-ISVEHAREYGHTHLFEIASQS--DKLGNKAILLIHFSARYTTEEIDAAINR----LPPSFRSRVYALKEGF----------**

**SitTrz3 (230) LRAKILVVESTFLDDESHSVEHARKYGHTHLSEIARQS--DKLENKAILLFHFSARYTTEEIDAAINR----LPPYFRSRIYALKEGFE--------**

**SbiTrz3 (234) LKAKILVVESTFIDDS-KSIEDARERGHIHLSEIVSLS--DKLKNKAILLNHFSLRYTAEILFSFLKKVISSTLVCLGQRIFIVTFEPIFNTFS---**

**SitTrz1 (224) LKAKILVVESTYIDDS-KSIEDAREKGHTHLSEIASLS--DKLENKAILLNHFSNRYTAEDIDVAINR----LPPPFRSRVYALKEGF----------**

**CsaTrz1 (217) LRAKILVMESTFLENR-VKVEHAREYGHTHLFEIINHA--KKFKNKAILLIHFSARYTTEVFYYLFDR---YGCCGLRNYSTSLKEAGFSC------**

**AlyTrz1 (200) LKAKVLVMESTFLDDS-VSVEHARDYGHIHLSEIVNHA--AKFENKAILLIHFSARYTVKEIEDAVSA----LPPPLEGRVFALTQGF----------**

**AthTrz1 (200) LKAKVLVMESTFLDDS-VSVEHARDYGHIHISEIVNHA--EKFENKAILLIHFSARYTVKEIEDAVSA----LPPPLEGRVFALTQGF----------**

**PpeTrz1 (223) LKAKILVVESTFLDDS-VKVEHAREYGHMHLSEIISHA--EKFENKAILLVHFSARYTVKEIEQAVSV----LPSPLAGRVFALTEGI----------**

**MesTrz1 (217) LRARILVMESTFVEDT-VKVEHARDYGHTHLSEIVSYA--DKFENKAILLIHFSARYTVEDIQEAISR----LPPPLAGRAFALTEGF----------**

**RcoTrz1 (220) LRARILVMESTFIDDT-VKVEHARDYGHTHLSEIVNYA--DRFENKAILLIHFSARYTVEEIQEAISR----LSPPLAGRVFTLTEGF----------**

**PtrTrz1 (221) LRAKVLVMESTFVDGT-VTVEHARDYGHTHLFEIVNYA--DKFQNKAILLIHFSARYTVKEIQEAVQR----LPQPLAGRVFALTEGF----------**

**CclTrz1 (208) LRARILVMESTYVDDS-TTVEQARDYGHTHLSEIVEYA--EKFENKAILLIHFSARHKVDEIRRAVDA----VPAPLAGRVFALTEGF----------**

**CsiTrz1 (208) LRARILVMESTYVDDS-TTVEQARDYGHTHLSEIVEYA--EKFENKAILLIHFSARHKVDEIRRAVDA----VPAPLAGRVFALTEGF----------**

**CpaTrz1 (217) LSAKILVMESTFVDES-VEVVHARDYGHMHLSEVVNYA--DRFKNKAILLIHFSARYMLEEIQKAVSA----LSPPLAGRVFALTEGFDGWSGEKNI**

**GmaTrz1 (199) LRARILVLECTFVNNS-ITVEHARDYGHTHLSEIISYA--ESLQNRAILLIHFSARYTVEEIQHAVSA----LPPSLSGRTFALTEGF----------**

**MtrTrz1 (243) LRAKVLVLESTFVNNE-MTVEHARDYGHTHLSEIISYA--DKLQNKAILLIHFSARYTVEEIQQAVSA----LPPPLAGRTFALTEGF----------**

**MguTrz1 (226) LRAKILIMESTFVEDS-SKVEDAREYGHTHLSEIIGYA--DMFQNKAILLIHFSARYQLDVIEKAISG----LPPPLAGRVFALTKGF----------**

**AcoTrz1 (203) LSAKILVLESTYVDNT-MTVQNARDYGHIHLSEIKYHA--DRFHNKAILLIHFSARYKLDEIKAAVSA----MPPSLEGRVFALTNGF----------**

**EgrTrz1 (229) LRAKILIMESTFVDDK-VTVEHAKDYGHTHLSEIVGCA--DRFANKAILLIHFSARHKREEIEAAISA----LGPPLAGRVFAMTEGF----------**

**VviTrz1 (210) LMARVLIMESTFVDNT-VTVEHAREYGHTHLSEIVSYA--DRFQNKAILLIHFSARYTTDEIQEAVSA----LPPPLGGRVFALTEGF----------**
