# Supplementary material for: A survey of green plant tRNA 3'-end processing enzyme tRNase Zs, homologs of the candidate prostate cancer susceptibility protein ELAC2
Source: BMC Evol Biol. 2011 Jul 23;11:219. doi: 10.1186/1471-2148-11-219 (PMC3161902; doi:10.1186/1471-2148-11-219)
Supplement: Additional file 5 — Alignment of candidate tRNase ZLs identified in green plants. The accession numbers for the candidates are listed in Additional file 1. The annotation of the alignment is as described in the legend for Figure 1. [file 1471-2148-11-219-S5.DOC]

**Additional file 5: Alignment of candidate tRNase Z^L^s identified in green plants**

**CreTRZ2 ( 1 ) -----------------------------------------------------------------------------------------------------------------------------**

**VcaTRZ2 ( 1 ) -----------------------------------------------------------------------------------------------------------------------------**

**MpuTRZ3 ( 1 ) ------------------------------------------------------------------------------------------MLAGAFRCASRN------ARR--------------**

**OluTRZ3 ( 1 ) -----------------------------------------------------------------------------------------------------------------------------**

**PpaTRZ4 ( 1 ) ----------------------------------------------------------------------------------------MSLHRPLMHHWHFPVPSLSRFRGPGSAPASS------**

**SmoTRZ3 ( 1 ) -----------------------------------------------------------------------------------------------------------------------------**

**SmoTRZ2 ( 1 ) -----------------------------------------------------------------------------------------------------------------------------**

**AlyTRZ3 ( 1 ) -------------------------MINSMPYLH--KNLRLLLLLSSKS-------------SPFPLSLRPFSPRS-------------FSLSTLFSSSSSS-----------------------**

**AthTRZ3 ( 1 ) -------------------------MINSMPYLH--KNLRLLRLLSSKS-------------SPFPLSLRPFSPRS-------------FSLSTLFSSSSSSSS---------------------**

**AlyTRZ4 ( 1 ) ------------------------MLTSSMPHRHVPQNLSLFGFSPLKS-------------SSFALFLRPFSLYP-------------PIFASSSPSPSRRPPRTAGYRRSDPSHPPRRKWSSL**

**AthTRZ4 ( 1 ) ------------------------MLTSSMP-----QNLSLFGFSPLKS-------------SSFALILRPFSLYP-------------PIFASSSPAPSRRPPRTAGYRRSGPSPP-RRKWSSF**

**MguTRZ3 ( 1 ) MPQSTNLRLLLSSANCHRRHPFSAASNFFPKHLSFSSSFQFFLKPQFKT-------------REIPLLFATFSSYSK------------KPYATNNNSNNNNKNSRSFNRNRSTLSETAKKGDNN**

**GmaTRZ3 ( 1 ) --------------------------------MAQVSKFGYFLLHS---------------------SLPKPSN---------------IQFRSLLTVLASSS---KRHR---------------**

**GmaTRZ4 ( 1 ) --------------------------------MAQVSKFGHLLLHS---------------------SLPKPSNSN-------------IQFRSLLTLLASSS---KRHRSIPP-----------**

**MtrTRZ3 ( 1 ) --------------------------------MAQILNFRNFLFLP---------------------SYKPTTH---------------FRLRFLSTLVSSSS---RRSNINAPPL---------**

**CsaTRZ3 ( 1 ) ------------------------------MPLPHLSTLRFLFFSP-------------------SKLPFSPSLYS-------------PKSHSLFTVLASSP---PKRRRSATAPPSLNFKRRN**

**CclTRZ3 ( 1 ) -------------------------------MPFITPNLRLLFSSSSSS------------LFPLKLSVPLLSTKP------------TNRHRSLFTILSYSK----RQRSTPFPQQNQRRNRST**

**CsiTRZ3 ( 1 ) -------------------------------MPFITPNLRLLFSSSSSSS-----------LFPLKLSVPLLSTKP------------TNRHHSLFTILSYSK----RQRSTPFPQQNQRRNRST**

**CpaTRZ3 ( 1 ) -------------------------------MFLIYPNLRLLLN------------------PPLFLFSK-PNSTP----------------LSLFTVFASSSH---KRHRSVSYRDSPFGLHRR**

**MesTRZ3 ( 1 ) --------------------------------MPQISNLRFLLSPIKP-------------------SLPFPFS-K-------------PKPYSLFTVLCSSS--SSRRHRTTPNHQSLNFRSRS**

**PtrTRZ3 ( 1 ) --------------------------------MSHISNLRLLLSPLNP-------------------TLRFPFSSK-------------HRPYSLLTILSSSSPYPKRRHRTTPNHPSLNFRSRS**

**EgrTRZ3 ( 1 ) -------------------------------MPCVYSNLRLLFSSSATAAAATATAAASPFLSPLKLKLRRPSSSSSAFPLLPLPPLSSLRSRPLFTIFAAVEP---RRRGRRLRHSPDFARGRS**

**PpeTRZ3 ( 1 ) --------------------------------MPQVTNLRLLFFS-----------------PFPRLSLSSLSFKP-------------LKPRTLFTALASSY---RKRHRPIPNQSPNTG-ARN**

**VviTRZ3 ( 1 ) --------------------------------MPHLTSFRLLYCSPLLSPFKS---------PFLSFSTLSKSKSP-------------LLNPPSFFTVLSSS---SGRYPKLRRHPHHLR-RRN**

**AcoTRZ3 ( 1 ) -------------------------------MPQISSNLKLFFSKTNHS------------------PLFQFSFKASFCSSFLLSSKTPYKPLSSVTVISSSSSSSSSSRKGPKFPPLR------**

**AcoTRZ4 ( 1 ) -----------------------------------------------------------------------------------------------------------------------------**

**MesTRZ4 ( 1 ) --------------------------------MPNVLNFKLCLSDLLTQCCK----------ICLQHYILTFSSLS--------------HVLVSGKLQSSNPQTYFSINNPLPRSK--------**

**BdiTRZ3 ( 1 ) -------------------------------MPQVAAPLRLLLPLSQTLAP---------PAPLLHLSRRLLSFCS---------PASFRRAASLRALAYR--------RSRHPE----------**

**SbiTRZ4 ( 1 ) -------------------------------MPQVAGPLRRLLPLSQTLASA--------PAPLLHLSRRLLSSCS---------PASFGRAASLRALAYR--------RRRHPE----------**

**ZmaTRZ3 ( 1 ) -------------------------------MPQVAAPLRLLLPLSQTLAA---------PAPLLHHSRRLLSSC----------PASLSRAAGLRALAYR--------RRRHTE----------**

**SitTRZ5 ( 1 ) -------------------------------MPQVAAPLRLLLPLSQTLAP----------APLLHLSRRLFTSS----------SPSFGRAASLRALAYR--------RHHHP-----------**

**OsaTRZ3 ( 1 ) -------------------------------MPQLPSPLRRLLPLSQTLAAAT-------PAPLLHLSRRLFSSSSSP------SPSPSPRAACLRALAYRGGQAGGGGRRGHHNNL--------**

**ψ PxKxRN ψ Motif I**

**CreTRZ2 ( 1 ) ------------MGKNKGAAAEPQGQ---------------AKQDKKGKQKQE-RQPQQ---RFERK---EGGLTHYSSFLQVLP--MDIEHTSPA-VLLFFDKERYLFNAGEGIQRLFREHRLK**

**VcaTRZ2 ( 1 ) ------------MGK-KQAQAEVQPQ---------------TKQDGKRKQKQQQTQPQAQQKRSERS---AGGMRHYSSYLQVLA--LDINHTSPS-VLLFFNNERYLFNAGEGIQRLFREHKIK**

**MpuTRZ3 ( 16) ----------HARASASAEAFSAEAMG--------------KRRAKKAPGAGYEVCARDGRVYDARGGELGRKQVGGDAYAQVVALGVDAGEDTSPGVLIFTNRRRYVFNVGEGFQRFCVEHGVA**

**OluTRZ3 ( 1 ) -----------------------------------------------MSTRRVVETLARRMSGPSR------ARRVGGAYVQVLHPGADARDATCASVLLCTDDARVVFNVGEGFQRLCVERRVK**

**PpaTRZ4 ( 32) ----------WTRASLSGSAFVQNWSQFR----------GSSFFSSASSSLLLRGSWFGRIGVRSS--------STQSFSLQILGTGMDTGDTSPC-VLLFFDQRRFIFNAGEGLQRFCIEHKIK**

**SmoTRZ3 ( 1 ) --------------------------------------------MRDPREASATSRFDSLLFLLCD--------AHSGELLQIFGTGMDTGETSPC-VLLFFDSKRFVFNAGEGLQRFCQENKIK**

**SmoTRZ2 ( 1 ) ---------------------------------------------MAVEAMRLPRAVSLRPPQRSS--------KLGTSYIQILGTGMDSEDTMPS-VLLFFDNKRFIFNAGEGMQRFCVEHKIK**

**AlyTRZ3 ( 50) ------------MENNESTNGTKSSS--------------FVFNKRRAEGIDKTDKKKRNLERKTQ----KLNPSNTIAYAQILGTGMDTQDTSSS-VLLFFDKQRFIFNAGEGLQRFCTEHKIK**

**AthTRZ3 ( 52) ------------MENNEATNGSKSSSNS------------FVFNKRRAEGFDITDKKKRNLERKSQ----KLNPTNTIAYAQILGTGMDTQDTSSS-VLLFFDKQRFIFNAGEGLQRFCTEHKIK**

**AlyTRZ4 ( 76) EEQKRKG--RSPMEKEKASSFNHSSDS-------------FEFNKRRAEGLDKIDKPKKNLKRNTR----TLNPTNTIAYVQILGTGMDTQDTSPS-VLLFFDKQRFIFNAGEGLQRFCTEHKIK**

**AthTRZ4 ( 70) EEQKRKG--RSPMEKDKAISFNHSSDS-------------FEFNKRRAEGLDKVDKPKKNLKRNTR----TLNPTNTIAYVQILGTGMDTQDTSPS-VLLFFDKQRFIFNAGEGLQRFCTEHKIK**

**MguTRZ3 (101) NNNNKNN--SDKGGFFAMEEKNTSADEK------------FGFNRKRAEGRDDSDRPKKHLQLKSR----KLNPANTISYVQILGTGMDTQDTSPS-VLLFFDKQRFIFNAGEGLQRFCSEHKIK**

**GmaTRZ3 ( 40) ----------RKSTTPKPMEVKEES---------------SSFNKRRAQGRDKNDISKKNLLLKVR----KLNPINTISYVQILGTGMDTQDTSPS-VLLFFDNQRFIFNAGEGLQRFCTEHKIK**

**GmaTRZ4 ( 46) ---FR-----RKSTTPKPMEVKEES---------------SSFNKRRAQGRDKNDISQKNLYLKVR----KLNPINTISFVQILGTGMDTQDTSPS-VLLFFDNQRFIFNAGEGLQRFCTEHKIK**

**MtrTRZ3 ( 46) HLRRR-----STTTSTTPMEVEENSS--------------VGFNKRRAEGTENSGLPKKNLQLKVR----KLNPINTISYVQVLGTGMDTQDTSPA-VMLFFDKQRFIFNAGEGLQRFCTEHGIK**

**CsaTRZ3 ( 61) SSSLR-----ETKGKENSVPMEETEA------------TSFGFNKRRAEGRDKTDLPKKNLQLKVR----KLNPANTISYVQILGTGMDTQDTSPS-VLLFFDKQRFIFNAGEGLQRFCTEHKIK**

**CclTRZ3 ( 67) FKKEK-----DKEDHSMEESAKE---------------SSFGFNKRRAEGRDKDVHDKKKLQLKVR----KLNPINTLSYVQILGTGMDTQDTSPS-VLLFFDNQRFIFNAGEGLQRFCTEHKIK**

**CsiTRZ3 ( 68) FKKEK-----DKEDHSMEESAKE---------------SSFGFNKRRAEGRDKDVHDKKKLQLKVR----KLNPINTLSYVQILGTGMDTQDTSPS-VLLFFDNQRFIFNAGEGLQRFCTEHKIK**

**CpaTRZ3 ( 57) RRNFT-----TFKERDKRTSMEENESSSS--S----SSSSFGFNKRRAEGLDKNDRPKKNLQLKVR----KLNPTNTIAYVQILGTGMDTQDTSPS-VLLFFDKQRFIFNAGEGLQRFCTEHKIK**

**MesTRZ3 ( 59) NSTSSREDNSKSRERGKGLPMEDTGN------------GTSGFNKRRAEGIDKSDKPKRNLQLKTR----RLNPTNTISYVQILGTGMDTQDTSPS-VLLFFDKQRFIFNAGEGLQRFCTEHKIK**

**PtrTRZ3 ( 62) KTTS--R---ETRDRDKGQSMDESGK------------ENFGFNKKRAEGRDN---PKRNLQLKVR----KLNPINTISYVQILGTGMDTQDTSPS-VLLFFDKQRFIFNAGEGLQRFCTEHKIK**

**EgrTRZ3 ( 92) RRAFS-----DDPGRDKRASMEE---------------GAAGFNRKRAEGRDRSDGPRKELQLKKRP---RANPVSTISYVQILGTGMDTQDTSSS-VLLFFDHQRFIFNAGEGLQRFCTEHKIK**

**PpeTRZ3 ( 60) KTTLR-----ESRGRDKAMEETKET-------------ETAGFNKRRAEGNDKNDRPKKNLQRKVR----TLNPINTLSYVQVLGTGMDTQDTSPS-VLLFFDKQRFIFNAGEGLQRFCTEHKIK**

**VviTRZ3 ( 68) SSSFR-----ETNRRDKGMSTEETES------------GSVGFNKRRAEGRDKNDRP-KTLQLKAR----KLNPVNTICYVQILGTGMDTQDTSSS-VLLFFDKQRFIFNAGEGLQRFCTEHKIK**

**AcoTRZ3 ( 71) -----------SRSNTTLSGNNKNSKEKV--EFMEEASSSIGFNKRRAEGKDKNDKFKN--LQLKTR---KLNPVNTISYVQILGTGMDTQDTSPS-VLLFFDKQRFIFNAGEGLQRYCTEHKIK**

**AcoTRZ4 ( 1 ) ----------MEEISTKADKLNKNKK-------------------RKAKGKDKPINCSS--KKVRTM---DSN--NSTAYFQILGNGMDTQDTSPS-ILLFFEKQRFIFNAGEGLQRYCTEHGIK**

**MesTRZ4 ( 62) ----------NSRTESKILSMVEETKT-------------YRYNESRAKGVDKKGHP-QKFQLSGR----TVHNPNTTAYVQVLGTGMDTHDTLPS-ILLFFDKQRFIFNAGEGLQRFCAEHKIK**

**BdiTRZ3 ( 59) ----------PRRGSSTLGKAP----------AKEEMDKEVAFNRKRAEGKDG--AKRGSMELKTR----RLNPVNTTCYVQILGTGMDTQDTSPS-ILLFFDKQRFIFNAGEGLQRFCTEHKIK**

**SbiTRZ4 ( 60) ----------PRRGSSTLGKAP----------AKEEMDREVAFNRKRAEGKDG--GKRGTMELKAR----RLNPVNTTCYVQILGTGMDTQDTSPS-ILLFFDKQRFIFNAGEGLQRFCTEHKIK**

**ZmaTRZ3 ( 58) ----------PRRGTSTLGKAP----------AKEEMDKEVAFNRKRAEGKDG--GKRGSMELKTR----RLNPVNTTCYVQILGTGMDTQDTSPS-ILLFFDKQRFIFNAGEGLQRFCTEHKIK**

**SitTRZ5 ( 56) -----------RRGSSTLRKPPRE-------EMAGGGDKEVAFNRKRAEGNDG--GKRGSMELKTR----KLNPVNTTCYVQILGTGMDTQDTSPS-ILLFFDKQRFIFNAGEGLQRFCTEHKIK**

**OsaTRZ3 ( 74) ----------LRRGNSTLGKRSKEKMGGGGGGGGGGGEAEVAFNKTRAEGKDG--RKGRSMELKSR----KLNPINTICYVQILGTGMDTQDTSPS-ILLFFDKQRFIFNAGEGLQRFCTEHKIK**

**ψ Motif II**

**CreTRZ2 ( 89) IRQ-VNAYFITRVSTETMSGLAGMALSVTPGDAAGL--------LGKQVSANIKGPRG-LAGYVAAFRTYVNKENTVAVEEFDSNTTEP-----LLKSDVVSITPFLVHSAVSSGSAAAAAEAEA**

**VcaTRZ2 ( 92) ISQ-INAYFITRITTETLGGLPGMALSVLPADAGGL--------LSKQVACAVKGPPG-LSSYVAAFRNYINQENTVAVEEFDHATSTP-----VLQTDVVSITPILVRVGETNISSEGPPVAAD**

**MpuTRZ3 (117) LRK-LERVFLTRASARTSGGLTGMLLTVADERGNPVATGAQRSEACDIPELTVHGPPR-VGRLLGAFHTLVGTNRNVKVNSRPFELTAPGGP-TERHPHAHKDEQMEVTPVVISLPATTATRGGA**

**OluTRZ3 ( 73) ALKRCERVLLTRADSRAAGGLVGTLLTMSDEAETRRGLDA----GVERRRVEVCGPDGRLRALTRAVRTLFGSGRAVELTTRGAARRAR---------S------VVADDGKVAISAVVLGGETR**

**PpaTRZ4 (128) LSK-IDHIFLTRVCSETAGGLPGLLLTLAGIGDA-------------GMAVNIWGPSE-LKYLVDAMRTFVPGASVVHTHSFGGSVGSRNEG---TKAQAEKSSEVLLEDDVVKITAVLLRSQTS**

**SmoTRZ3 ( 73) LSK-VDHVLLTRVCSETAGGLAGALLTLA--GDV-------------GLTLNIWGPSD-LQCLITAMSTYLPNASFFYTHAFGKDGKLLPEH---AKDPN---IAVLLEDDLVKISAVLLVPFSD**

**SmoTRZ2 ( 72) LSK-IDHILFTRVCSETCGGLPGALLTLAR--DL-------------EATVNIWGPSK-LQFLLNAMSTFIPNSSILHAHIFGSDTMPQ--------------GGTIIDDDVVKISGTLLQPCNG**

**AlyTRZ3 (144) LSK-IDHVFLSRVCSETAGGLPGLLLTLAGIGEE-------------GLAVNVWGPSD-LNYLVDAMKSFIPRAAMVHTRSFGPSS--------------TPDPIVLVNDEVVKISAIILKPCHS**

**AthTRZ3 (148) LSK-IDHVFLSRVCSETAGGLPGLLLTLAGIGEE-------------GLSVNVWGPSD-LNYLVDAMKSFIPRAAMVHTRSFGPSS--------------TPDPIVLVNDEVVKISAIILKPCHS**

**AlyTRZ4 (181) LSK-VDHIFLSRVCSETAGGLPGLLLTLAGIGEE-------------GLSVNVWGPSD-LKYLVDAMRSFIPRAAMVHTRSFGPSLNASDSTPQSGLSKPKDDAYVLVDDEVVKISAILLEPSHL**

**AthTRZ4 (175) LSK-VDHIFLSRVCSETAGGLPGLLLTLAGIGEQ-------------GLSVNVWGPSD-LKYLVDAMRSFIPRAAMVHTRSFGPSLNISDSAPQIGLSKPKDDAYVLVDDEVVKISAILLEPSRL**

**MguTRZ3 (207) LSK-IDHIFLSRVCSETAGGLPGLLLTLAGMGDE-------------GMSVNVWGPSD-LKYLVDAMKSFIPNAAMVNTRSFGPTPDSNESSR--SMPHRIDEPFVLIDDEVVKLSAILLRPSLL**

**GmaTRZ3 (135) LSK-IDHIFLSRVCSETAGGLPGLLLTLAGMGEE-------------GMSVNIWGPSD-LKYLVDAMRSFIPNAAMVHTKSFGPISNIDGPI--VQCQSKLLDPIVLIDDEVVK-ISAIIL----**

**GmaTRZ4 (143) LSK-IDHIFLSRVCSETAGGLPGLLLTLAGMGED-------------GMSVNIWGPSD-LKYLVDAMRSFIPNAAMVHTRLLDPIVLIDGEV--VKISAIILQPNCIEGQVLTPSESSSQE----**

**MtrTRZ3 (147) LSK-IDHIFLSRVCSETAGGLPGLLLTLAGMGDE-------------GMTVNVWGPSD-LKYLVDAMRSFIPNAAMVHTKSFGPTFGTESTV--KS-QS---DPIVLVDDEVVKISAIILQPC--**

**CsaTRZ3 (164) LSK-IDHIFLSRVCSETAGGLPGLLLTLAGIGDV-------------GMSVNVWGPSD-LKYLVDAMKSFIPNAAMVHTRSFGPTVSSDADA--VHELSKCREPIVLVDDEVVKISAILVHPRPN**

**CclTRZ3 (167) LSK-VDHIFLSRVCSETAGGLPGLLLTLAGIGDE-------------GLSVNVWGPSD-LKYLVDAMKSFIPHAAMVHTHCFGPAPSSDAS---LPDSAKSANHIILVDNELAKISAILLKPSCS**

**CsiTRZ3 (168) LSK-VDHIFLSRVCSETAGGLPGLLLTLAGIGDE-------------GLSVNVWGPSD-LKYLVDAMKSFIPHAAMVHTHCFGPAPSSDAS---LPDSAKSANHIILVDNELAKISAILLKPSCS**

**CpaTRZ3 (166) LSK-VDHIFLSRVCSETAGGLPGLLLTLAGIGEE-------------GMTVNVWGPSD-LKYLVDAMRSFIPRAAMVHTRSFGPESCSDTNI--LPDPSKFVDPIVLVDDEVVKVSAILLRSSCS**

**MesTRZ3 (167) LSK-IDHIFLSRVCSETAGGIPGLLLTLAGMGEE-------------GMSVNLWGPSD-LQYLVDAMKSFIPHAAMVHATSFGSDATA------QSHASNFMNPIGLIKNEVVKISAILLRP---**

**PtrTRZ3 (162) LSK-IDHIFLSRVCSETAGGIPGLLLTLAGMGEE-------------GMSVNIWGPSD-LKYLVDAMKSFIPHAAMVHTKSFGSDNVG------LVDANKFIDPIVLINDEVVKISAILLRP---**

**EgrTRZ3 (193) LSK-IDHIFLSRVCSETSGGLPGLLLTLAGIGDE-------------GMSVNIWGPSD-LKYLVDAMKSFIPNAAMVHTNSFGPAPSSDAAA--VPDLNQFADPIILVDDDVVKISAVLMWPEES**

**PpeTRZ3 (162) LSK-IDHIFLSRVCSETAGGLPGLLLTLAGMGEE-------------GMSVNVWGPSD-LKYLIDAMRCFIPNAAMVHTRSFGPSVG---GL--MASQTKFTEPIVLVDDEVVKISAIVLQPIFS**

**VviTRZ3 (170) LSK-IDHIFLSRVCSETAGGLPGLLLTLAGMGDE-------------GMSVNIWGPSD-LKYLVDAMRSFIPNAAMVHTRSFGQALGSDGAP--IPDLREFSDPIVLIDDEVVKISAILLRP---**

**AcoTRZ3 (177) LSK-IDHIFLSRVCSETAGGLPGLLLTLAGMGDA-------------GMSVNIWGPSD-LKFLVDAMRSFIPNAAMVHTHSFGSTSSADGAKVS--SQEKFAEPIVLIEDEVVKISALLIRPMAL**

**AcoTRZ4 ( 89) LAK-IDHILLSRVCSETAGGLPGILLTLAGMGEE-------------GLSVEVWGPSD-LHHLGEAMESFIS-ANVIHTRCFGPTPEGDGATDS--GFGKLVN--LPIPGQAVKISAIPIKPSSH**

**MesTRZ4 (158) LSK-IDHICLSRVCSETAGGLPGLLLTLAGMGN--------------GLSVNIWGPPS-LELLINAMKSFIPHGSVVNAKEIGLTTCSSSAA--LLDTSNSAEPFVVVENELVKISAILLLPSSL**

**BdiTRZ3 (157) LSK-IDHIFLTRVCSETAGGLPGLVLTLAGIGDE-------------GMSVNIWGPSD-LDFLAGAMRSFIPNRAMLHTHSFGTDQNAS--------SPQSKDSIIILDDEVVRISAMFVKPRYH**

**SbiTRZ4 (158) LSK-IDHIFLTRVCSETAGGLPGLVLTLAGIGDE-------------GMSVNIWGPSD-LDFLAGAMRSFIPNRAMLHTHSFGTDRNAS--------SPQSKESIIILDDEVVRISAMFVKPRYH**

**ZmaTRZ3 (156) LSK-IDHIFLTRVCSETAGGLPGLVLTLAGIGDE-------------GMSVNIWGPSD-LDFLAGAMRSFIPNRAMLHTHSFGADRNAS--------SPQSTDSVIVLDDEVVRVSAMFVKPKYN**

**SitTRZ5 (156) LSK-IDHIFLTRVCSETAGGLPGLVLTLAGIGDE-------------GMSVNIWGPSD-LDFLAGAMRSFIPNRAMLHTHSFGAERSAS--------SSQPKDSVIILDDEVVRISAMFVKPKYH**

**OsaTRZ3 (182) LSK-IDHIFLTRVCSETAGGLPGLVLTLAGIGEE-------------GMSVNIWGPSD-LDFLASAMRSFIPNRAMLHTHSFGVEQNVS--------SSQSKDATVIVDDEVVRISAMFVKPRYN**

**Flexible arm**

**GP motif**

**CreTrz2 (199) AKGSPAKGSRAERGHS--PAAKRRRVAPDNSPEMSDAEEDAKAAAEGRERPADLSPPATAVYLVRLSGRPGKFLPDKA-RQLGVKPGPLFGELQRGHSVTLEDG--RVVTGADVCEPPVPGPAVM**

**VcaTrz2 (202) GDG------DGEGDGG--PAAKRQRTEPSS----TANEQDS---P-GRQRPAGLSHPDIAIYAIQLAGRPGKFMPEKA-IELGVEPGPLFGQLQRGLSVTTKSG--RVVSPGEVMEAAVPGPAVL**

**MpuTrz3 (239) SGPFGDVASLGRHVAKRQKTMEEGGGDVGGLDDAGDPGSNGHNQGVTSALVLDETEAEAVCYGLQMAPSAGKFDNAKA-EALGIPKGPWCSDLVHGYSITLENG--TVIEPSMVVGAPTTGARIF**

**OluTrz3 (179) DDGTDSAVG----------------------------------RAAKRARADEDASDEVASYDVKLAAIPGKFDMRAA-VELGVPNGPQRGRLVRGESITLDDG--SVVSPEMCVGPEQPGPRVV**

**PpaTrz4 (235) LPSDKK------------------RSKRARVEE-----------TTAESSFADESYDVSVAYVCELPEVKGRFDIEKA-RKFFNRPGPHYGLLQSGKSVLASDG-VTMVHPEDVMDPSSPGPIFI**

**SmoTrz3 (175) KQGEG--------------------SSKK-----------------FR------PGDVAVVYVCELPEVRGKFDPVKA-AALGLRPGRKYALLQSGTSVLSDDG-TTEILPESVMEPSSPGPKVI**

**SmoTrz2 (166) KNCEGT------------------ILLVP-----------------------------SVVYVCELPEVKGRFDPAKA-VSLGLQPGQKYGQLQRGMTVETDDG-ARTIHPDDVMEPSTSGPVFI**

**AlyTrz3 (240) EEDS-----------------------------------------------GNKSGELSVVYVCELPEILGKFDLEKAKKIFGVKPGPKYSKLQSGESVKSDER-DITVYPSDVMGPSLPGPIVL**

**AthTrz3 (244) EEDS-----------------------------------------------GNKSGDLSVVYVCELPEILGKFDLEKAKKVFGVKPGPKYSRLQSGESVKSDER-DITVHPSDVMGPSLPGPIVL**

**AlyTrz4 (291) EES------------------------------------------------GSKPGETAVIYVCELPEIKGKFDPKKA-MALGLRAGPKYSYLQSGQSVKSDFK-DITVHPSDVMGPSVPGPVVL**

**AthTrz4 (285) EES------------------------------------------------GSKPGETAVIYVCELPEIKGKFDPKKA-MALGLRAGPKYSYLQSGQSVKSDFK-DITVHPSDVMGPSVPGPVVL**

**MguTrz3 (315) EVSEP------------------------------------------MKEKSSANGDISVVYICELAEIKGKFDPKKA-IALGLRPGPKFRELQLGNSVKSDNQ-DVMVHPSDVLGPSVAGPIVI**

**GmaTrz3 (238) --------------------QPNCIEETLDS-------------PNGKKLSAAKPGDMSVVYVCELPEIKGKFDPEKA-KALGLRPGPKYRELQLGNSVKSDRQ-NIMVHPSDVLGPSVPGPIVL**

**GmaTrz4 (247) --------------------RMDHSPETLDS-------------PNGKKLPAAKPGDMSVVYVCELPEIKGKFDPEKA-KALGLRPGPKYRELQLGNSVKSDHQ-NIMVHPSDVLGPSVPGPIVL**

**MtrTrz3 (249) -------------QIP--SQKTDHSIDIADS-------------LNGKKLLAAKPGDMSVVYVCELPEIQGKFDPEKA-KALGLRPGPKYRELQLGNSVESDRQKNVMVHPSDVMDPSIPGPVVL**

**CsaTrz3 (272) R----RLGLFDEDDMG--HSQEQRRNDNSEARS-----------STTKTSSTVKPGDMSVVYVCELPEIKGKFDPSKA-AALGLKPGPKYRELQLGNSVMSDHQ-KIMVHPSDVLGPSVPGPVVL**

**CclTrz3 (274) DG------------------------------------------------SPVKPGETSVIYVCELPEITGKFDPKKA-VALGLKPGPKYRELQSGKSVKSDTL-DIMVHPSDVLGPSLPGPIVL**

**CsiTrz3 (275) DG------------------------------------------------SPVKPGETSVIYVCELPEITGKFDPKKA-VALGLKPGPKYRELQSGKSVKSDTL-DIMVHPSDVLGPSLPGPLVL**

**CpaTrz3 (274) VG------------------------------------------------SAVKPGEMSVLYVCELPEIMGKFDPKKA-VALGLKAGPKYGQLQSGKSVKSDRL-DIMVHPSDVMGPSVPGPIVF**

**MesTrz3 (268) ---------------------------------------------SYIEGAAVKPGDMSVIYVCELPEIMGKFDPEKA-KALGLKPGPKFSELQSGISVKSDHQ-DIMVHPSDVMGPSVPGPVVF**

**PtrTrz3 (263) ---------------------------------------------SQSQGSALKPGDMSVIYLCELREIMGKFDPEKA-KALGLKPGPKYRELQSGRSVMSDLQ-SIMVHPSDVMDPSVPGPIVL**

**EgrTrz3 (301) GQN-------------------------------------------NGVHSAMRPGDMSVVYVCELPELKGKFHPERA-KEKGLRPGPEYRELQLGKSVFSKKL-NITVHPSDVMDPSIPGPVVL**

**PpeTrz3 (267) NGAQLLNELSITQNPT--EKVFNDGVDVSKPFS-----------PNGKNSPTGKPGDMSVIYVCELPEIKGKFDPEKA-KALGLKPGSKYRELQLGNSVKSDFQ-NITVHPSDVMDPSIPGPIVF**

**VviTrz3 (275) ----------------------------SE---------------DSKAGAMVKPGDISVIYVCELPEIKGKFDPQKA-VALGLKAGPKYRELQLGKSVVSDRK-NIMVHPSDVMGPSIPGPLVL**

**AcoTrz3 (285) DEAP----------------------LLPHSRN-----------PEMFDSSSLKPGDISVIYVCELPEIKGKFDPAKA-AALGLKPGPKYRELQLGNPVKSDKQ-NIMVHPSDVLGPSIRGPIVL**

**AcoTrz4 (194) AVCQP--------------KENFTKLALNEGTE-----------GPLKSGLALKTDVISVIYVCELPEIKGKFDVNKA-KALGVPQGPKYGKLQCGYSVMSDDQ-NIMVHPSDVLAPSRPGPIVL**

**MesTrz4 (265) EG------------------------------------------------AGKKPSDISVIYVCELHEILGKFDKEKA-DALGLKERKKYGLLQKGECVKSDCL-DIMVHPSDVMDPPIPGPVVF**

**BdiTrz3 (259) NGTG------------------------------------------SSNDSDMKPGDTAIVYACELPEIKGKFDPSKA-AALGLRPGPKYRELQLGNSVQSDQF-DEMVHPSDVLGPSIPGPTVL**

**SbiTrz4 (260) NGTG------------------------------------------SSNDSDMKPGDTAIVYACELPEIKGKFDPSKA-AALGLRPGPKYRELQLGNSVQSDQF-DEMVHPSDVLGPSIPGPTVL**

**ZmaTrz3 (258) NGTG------------------------------------------SSNDSDMKPGDTAIVYACELPEIKGKFDPSKA-AALGLRPGPKYRELQLGNSVQSDQF-DEMVHPSDVLGPSIPGPTVL**

**SitTrz5 (258) NGAG------------------------------------------SLNDIDLKPGDTAIVYACELPDIKGKFDPAKA-AALGLKPGPKYRELQLGNSVQSDKF-DEMVHPSDVLGPSIPGPTVL**

**OsaTrz3 (284) KEAS------------------------------------------CLNDSNLKPGNTAIIYACELPELKGKFDPAKA-AALGLKPGPKYRELQLGNSVQSDAF-DKMVHPSDVLGPSIPGPTVL**

**CreTRZ2 (319) VVDTPDAASLAAVAAHPR---------LLSAAAEATAET----------DGAGRLCLVVHLLPAALAADGAA-LAAWRGALGPAWRHVVVSSGQ-----HQPSAIPRATVFQAQLHAVHPTAFPL**

**VcaTRZ2 (308) LLDTPSLESLRAVAKDER---------VRRFVADATAGEGNDGKAAVASGGSPRVCVAIHLMPAELAVNGAEDLAAWRAQLGPSWRHVVVSSGA-----QQPSSIPRATVFQAKLHALSAACFPV**

**MpuTRZ3 (361) IADVPTTRHLAEITREGSPSYNALRKLGHVSDKKVSDEDLAVSEDTDAAFPIGDLGCVFHLTPSSVANTPEY--EAWTRDCEATFPGVSLRPSHLAFN-PDTPRRLSAPPDAFQLHPDNKVFPPR**

**OluTRZ3 (267) VFDAPTTAHVREATTR-----------GGELFGELS-----------------DLSVVVHLASAAIVSTKEY--AALVKSVFGGAPKDAAHVFANSDAMDDVPVFASSARIQARLHAVSATVFPE**

**PpaTRZ4 (329) LVDCPTAAYIPALITN-P---------VLCSFQDQ---------------GSKQVTLVVHISPASISQLPEY--QSWMSRFAGAQHVMTGHGTLN----MSQPVLKSSARVVSRLNRICPQVFPI**

**SmoTRZ3 (255) LVDCPTMFHIPSLLTANG---------LKGLYTSD---------------GAASVACIVHMSPAIVVNDSKY--QDWMRLFGSTEHILAGSGCSN----SLSPILKSSAKILSKLNFACPYVFPV**

**SmoTRZ2 (242) VVDCPTVSHIPALTSAAS---------LEALYSSPR--------------TKAEVVCVVHLSPSPVVEDAGY--SCWMTRFAAAQHVLAGPGWSK----TRCPIFKSSALLLAKLNMVCPRVFPF**

**AlyTRZ3 (317) LVDCPTESHAAELFSVKS---------LDSYYSCTDDQT----------DGPKFVNCIIHLSPSSVTSSPTY--QSWMNKFHSSQHILAGHQRKN----MAFPILKASSRITARLNYLCPQFFPA**

**AthTRZ3 (321) LVDCPTESHAAELFSLKS---------LESYYSSPDEQT----------IGAKFVNCIIHLSPSSVTSSPTY--QSWMKKFHLTQHILAGHQRKN----MAFPILKASSRIAARLNYLCPQFFPA**

**AlyTRZ4 (366) LVDCPTKSHAEELLSIPS---------MKSYYSCLDNST----------DGAKLVNCIIHLSPASVTNSSTY--QSWMKRFHSAQHILAGHEAKN----MEFPILRASSRITARLNYLCPQFFPA**

**AthTRZ4 (360) LVDCPTESHAEELLSIPS---------MKTYYSCLDNST----------DGAKLVNCIIHLSPASVTNSSTY--RSWMKRFHSAQHILAGHEAKN----MEFPILRASSRITARLNYLCPQFFPA**

**MguTRZ3 (396) LVDCPTSSHLHELSSLKC---------LAPYYIDTAYDV---------PEGSKLVNCVIHLTPESVSKTEDY--RMWMSKFGGAQHIMAGHEMKN----IEVPILKASARIAARLNYLCPQFFPS**

**GmaTRZ3 (328) LVDCPTESHLEALLSVQS---------LASYCDQADN-Q---------PEAGKSVTCVIHLTPSSVVSCSNY--QKWMKKFGSAQHIMAGHEKKN----VEIPILKASARIATRLNYLCPQFFPA**

**GmaTRZ4 (337) LVDCPTESHLEALLSMQS---------LASYCDQTDN-L---------PEAGKSVTCVIHLTPASVVSCSNY--QKWMKKFGSAQHIMAGHEKKN----VEIPILKASARIATRLNYLCPQFFPA**

**MtrTRZ3 (345) VVDCPTESHLEALLSAKS---------LDTYGDQVGN-L---------PKAGKSVSCVIHLTPESVVCCSNY--QNWMKTFSSAQHIMAGHEKKN----IEVPILKASARIATRLNYLCPRFFPA**

**CsaTRZ3 (378) LIDCPTESHLSELMSLES---------LRPYYEDLSSDQ---------TETGKVVTCVIHLSPASILGNPNY--QKWARRFESAQHIMAGHHRKN----VAIPILRASAKIAARLNHLCPQLFPA**

**CclTRZ3 (349) LVDCPTESHVLELLSAES---------LNSYYADFSGD----------PQCAKTVNCIIHLSPVSVTGTSNY--QKWMKRFGSAQHIMAGHEMKN----VEIPILKSSARITTRLNYLCPQLFPA**

**CsiTRZ3 (350) LVDCPTESHVLELLSAES---------LNSYYADFSGD----------PQCAKTVNCIIHLSPVSVTGTSNY--QKWMKRFGSAQHIMAGHEMKN----VEIPILKSSARITTRLNYLCPQLFPA**

**CpaTRZ3 (349) LVDCPTEHHAQELLSTES---------LSIYYADFLGKS---------EGTTKTVNCIIHLTPASVIHTQSY--QEWMKRFGSAQHIIAGHEMQN----VEVPILKASARIAARLNYLCPQFFPA**

**MesTRZ3 (346) LVDCPTESHAQELLSIQS---------LNCYTADYSDSP---------PQNVKTVTCIIHLSPASVITSPSY--QKWMKKFFSSQHIMAGHEMKN----VEIPILKSSSRITARLNYLCPQFFPS**

**PtrTRZ3 (341) LVDCPTESHLQELLSMES---------LNNYYVDFSGNP---------TQSGKTVNCIIHLSPASVTSSPTY--QKWMKKFGSAQHIMAGHEMKN----VEIPILKSSARIAARLNYLCPQFFPA**

**EgrTRZ3 (381) LVDCPTQSHFQRLSSLGS---------LNDYYADFLG-----------KETQKAVTCVIHLSPASVVSSPDY--TNWMKRFGSAQHIIAGHEMKN----IEIPILSSSSRIAARLNYLCPQLFPA**

**PpeTRZ3 (377) LVDCPTESHLQELLSMQC---------LSSYYADFSG-P---------PENANVVTCVIHLGPASLISNPNY--QSWMKRFGSAQHIMAGHERKN----VEIPILRSSARIAAQLNYLCPQFFPA**

**VviTRZ3 (355) LVDCPTESYLQDLLSVES---------LSSYYAGSSSNP---------PESAKTVNCVIHLSPASVVRAPNY--QVWMKRFGAAQHIMAGHEMKN----VEIPILKSSARIAARLNYLCPRFFPA**

**AcoTRZ3 (375) LIDCPTPSHVQGLLSIQS---------LSCYFADSTKRQ---------PDDVKSVDCIIHLSPSSVTTTAIY--QKWMKRFGGVQHIMAGHEIKN----TEIPILKSSARIAARLNYLCPQFFPA**

**AcoTRZ4 (292) LVDCPTIMHLQELVTIQS---------LNNYYSDLQLYG------------SKSVNFIIHLSPPSVTTTVTY--QKWMARFGEAKHIMTGHEIKN----MKIPILQASVRVETQLNYLCPQFFPA**

**MesTRZ4 (340) IVDCPTNSHAKELLSIQT---------LNGYYPDFVGNS---------PKSSKAVNCIIHLTPPPVINSPNY--EKWMKKFPTVQHIMAGHSMKH----VEIPILKSSTRMAARLNYLCPQFFPV**

**BdiTRZ3 (340) LVDCPTQYHMPELFSLQP---------LSCFYEDS---P---------EQSGKKVNCIIHLGPSSVTRSVDY--QNWMKFFGTTQHIMAGHEIKN----MEIPILKGSARISSRLNFVCPQLFPS**

**SbiTRZ4 (341) LVDCPTQYHMPELFSLQP---------LSCFYEDS---P---------EQSGKKVNCIIHLGPSSVTRSVDY--QNWMKKFGATQHIMAGHEIKN----MEIPILKGSARISSRLNFVCPQLFPS**

**ZmaTRZ3 (339) LVDCPTQYHMPELFSLHP---------LSCFYEDS---P---------EQSGKKVNCIIHLGPSPVTRSVDY--QNWMKKFGATQHIMAGHEIKN----MEIPILKGSARISSHLNFVCPQLFPS**

**SitTRZ5 (339) LVDCPTQYHMPELFSLQS---------LSCFYEDSSD-P---------TKSGKTVNCIIHLGPSSVTKAVDY--QNWMKKFGATQHIMAGHEIKN----MEIPILKGSARISSRLNFVCPQLFPS**

**OsaTRZ3 (365) LVDCPTKYHMHELFSLQS---------LIRFYEDSSE-Q---------AGSPKKVNCVIHLGPSSVTEALDY--QNWMRKFGATQHIMAGHEIKN----MEIPILKGSARISSRLHFVCPHLFPS**

**CreTRZ2 (419) FALDNAGAAAAAAVAAVLAPQGQ--APVLPLPESGVVEGALSALRVNLVPPARQGLEYGDVFRRVYGN-VYDPPAATLELMRTNPDFAPVMAAAAAAQR----------------------ASGM**

**VcaTRZ2 (419) FALGNAVAPPPVAEAELDP------GSVIQAAS---------AVRVNLMPLTRLGLEYGDVFK-------YDSSSKVLEDVRADPAYAPILAAAATAVPGDSAAAAADGGANGKKTEATGMAAAD**

**MpuTRZ3 (483) SAKAAWTQRIAWATDR-------------------AKVADDNWVRYVLMPVDKQRVDATDARAPPAMSGSYINYDLAPHALKKIVADAEATRSAAAAKAEAEAEASS-------------ALELE**

**OluTRZ3 (362) NNVPRSPPPSPSKDDKRRDG---------VPADVPNALAGKNMFKFTLIPTKNAGADMSAVPRYVPG--FVHRRDIEQRTIDLANEAMKPTPPPSPSAEARPG---------------------A**

**PpaTRZ4 (423) SGLQSSGRQDQIKDTQEKDSSL------------DLISVGENLLKFRLRPLSSLGLDR---SAVPE---PFSMQHVQDQMLLDIPELLEATNKISDFWKSTSIESVSADALV-----SMDEVTKY**

**SmoTRZ3 (350) PGYGYESSKNQQILLQQQNN--------------LSVTIAKNLLKFRLLPLSGLGMDG---SSVPE---AFDGKVHTEELIECVPEILPLTEKLKEFWKAYG--------------------SKD**

**SmoTRZ2 (338) N-LGVDKSDTKFSEVKHSPVSISTELTCYYCRIHALWLLTTFSRQFQLLPLPLLGLEK---SPAVE---DFNLEVIQTEFLNEIPELLSIKQDVKLLYDENA-----------------------**

**AlyTRZ3 (417) PGFWP-QLADMSMIDSTPWN-------KCSSSNLAESISAENLLKFNLRPVAIRGIDR---SCIPA---RLTSSEVLDELFDEIPEIKDKCGEIMRFWNK--------------------QHDKT**

**AthTRZ3 (421) PGFWPSQLTDNSIIDPTPSN-------KCSSSNLAESISAENLLKFNLRPVAIRGIDR---SCIPA---PLTSSEVVDELLSEIPEIKDKSEEIKQFWNK--------------------QHNKT**

**AlyTRZ4 (466) PGFWS-PQHVNNSINSTSLS-------KCFDSNLGESISAENLLKFTLRPHSNLGVDR---TCIPS---RLTSLRVMDELLSEIPEISSKTEEIKRLWNG--------------------QHNKM**

**AthTRZ4 (460) PGFWS-HQHDNNSINPTSLS-------KCFDSNLGESISAENLLKFTLRPHGNLGVDR---SSIPS---RLTALRVMDELLSEIPEISSKTEEIKQLWNG--------------------QHNKM**

**MguTRZ3 (497) PGFWSLQNLNLLPSEAMASPP------KIPLLRTSALIPAENLLKFQLRPYANLGLDK---SSIPS---LSSPSEIIEELLSEIPEVKDASQQITSFWSDNKKD------I---------NMEKT**

**GmaTRZ3 (428) PGLWSLPNHNSSKFGCLASSEG-------SFSELSEVISAENLLKFTLRPYAHLGLDR---SCIPT---TAASSEIIDELLSEIPEVLEAVRHVSQLWQECSQTKEDLTPV---------ADHGM**

**GmaTRZ4 (437) PGLWSLPNHDSSKFGCLASRE--------SLSEFSEVISAENLLKFTLRPYAQLGLDR---SCIPT---RADSSEIIDELLSEIPEVLEAVKHVSQLWQECSQTKEDLTPV---------ADHGM**

**MtrTRZ3 (445) PGFWSLPNQNCSKPVSLASSED-------SFSAPSNVIYAENLLKFTLRPYVNLGLDR---SCIPP---KASSSEIIDELLLEIPEVVEAAQHVRQLWEDSSQAKEDSIPL---------ADHSE**

**CsaTRZ3 (479) PGFWSHQQLTMPGSDSCASTEI-------EVSNHYKSTLAENLLKFTLRPYAQLGFDR---SNIPS---QESLPEIINALHSEIPEIVDAVEHVSQLWRGSAET-DERTPV---------EENNA**

**CclTRZ3 (449) SGFWSLPHFNTSAAESSASEGP------------VPSICAENLLKFTLRPLANLGIDR---TNIPS---LEAPSEITNELLSEVPEVVDAAHQISQFWQGPREL-KDDCPM---------LDNEV**

**CsiTRZ3 (450) SGFWSLPHFNTSAAESSASEGP------------VPSICAENLLKFTLRPLANLGIDR---TNIPS---LEAPSEITNELLSEVPEVVDAAHQISQFWQGPREL-KDDCPM---------LDNEV**

**CpaTRZ3 (450) PGFWSLHHSNNSAVEPITSIKSP-------DSKLCESVSAENLLKFTLRPHAHLGLDR---SSIPT---PSTSSEVIDDLLSEIPEIVDAAQHVRQVSLG--------------------QDGKN**

**MesTRZ3 (447) PGFWSLKQLNCSNVEPIFSGED-------YVLKAPEIISAENLLKFTLRPHAHLGLDK---SNIPS---LIAPSDVIEELVTEIPEIVDAAQHVRQFWHGSGETKGYMTLG---------QDNKV**

**PtrTRZ3 (442) PGFWSLTHLNNSRPDSILSGEG-------CVSKLCENTSAENLLKFTLRPHAHLGFDK---SNIPS---LMAPSEIINELLTEIPEIVDAAKHVRKFWSGPGGLEADINAI---------QGNKV**

**EgrTRZ3 (480) SGFWSLEQSDSVHLSEGTSLS-------------CPSISAENLLKFTLRPRDKIGLDK---SYIPN---KMAHSNIIDELHTEIPEIVDAAQYIHHLWNGTGET-GEERRF---------MQ---**

**PpeTRZ3 (477) PGFWSLQHLDCLAPESTPSSEG-------SVSKVCESISAENLLKFTLRPYARLGLDR---SVIPS---QVASSEIIDELLSEIPEVVDAAQCVSQLWHQSTETKEEIRLT---------HDDKV**

**VviTRZ3 (456) PGFWSLRHLNHSIPELIASSEVCL--LSHCFDTLCESVAAENLLKFHLRPYAQLGLDR---SGIPS---LSSPSEIIDDLVSEIPEVVDAAQEVGQFWNGFGEAKGEITPM---------HDDKV**

**AcoTRZ3 (476) PGFWSLQNLKDFAPDSSEGSGP----------KLCDSISAENLLKFHLRPYAQLGLDR---SGIPS---CMSSKEVVNELLFDIPEIADSVEQVRQLWNGSADTERAKLHT---------QESII**

**AcoTRZ4 (390) SGFLSLQHIQDNNDLS-KGSGP----------KLCDSISGENLMKIQLLPQPRLNEENQHTKLIPE---LLDQEKIVNQLISEIPEIVDSVECVRQLWNESTDPNRE--S-----------TQEV**

**MesTRZ4 (441) PAVGSFQQHNDAAQGSITSSEV-------LISQLCESTSAENLMKFTLRPHNHLGLDK---SNVPS---LMAPSEVIDELLSENPEIVDAAQLVSQFWSEPGE--MEDTSI---------TDDTT**

**BdiTRZ3 (438) SGFWSIQPPNDVMEN-----------DKNASLEACGSVSAQNLLKFHLRPYAQLGLDR---ASIPN---LFTYKDIIQELLSEIPEVSEVPEQVRKFWQNNVNDKNTLPP-----------TGNR**

**SbiTRZ4 (439) SGFWSVQPPNDVMEN-----------DKNASLEACGSVSAQNLLKFHLRPYAQLGLDR---ASIPN---LFTYKDIVQELVSEIPEVSEVPEQVCKFWQNNVNDKNTLPP-----------TGNR**

**ZmaTRZ3 (437) SGFWSEQPSNDVMEN-----------DKSVSSEACGSVSAQNLLKFHLRPYAQLGLDR---TSIPN---LFTYKDFVEELVSEIPAIREVPEQVCKFWQNNVNDNNILTP-----------TGNH**

**SitTRZ5 (439) SGFWSVEPANDVMEN-----------DKRTSSEICGSVFAQNLLKFHLRPYAQLGLDS---ASIPS---LFTYKDIVEELVSEIPEVREVPEQVHKFWQNNVNYKNTL-----------------**

**OsaTRZ3 (465) SGFWPVEPINDVDSEK----------NKVSSLQACESVSAANLLKFHLRPYAQLGLDR---SSIPS---LTTYGDIVDELLSEIPEIKEVPEQISKFWQSNSVGKHML-----------------**

**PxKxRN Motif I**

**CreTRZ2 (519) QVEVPTAPTPAAADGAKAEGAAGAGDVEMAERGP-----------------ASAAAADMDPPSTVASGDRRQAELTFLGTASSQPSKFRNVSGCYVDLFE---RGGLLVDCGEDAMGQLARRYGG**

**VcaTRZ2 (522) VADAAVEGAGVDTDKTAAEAVEATEVMEVEVPEA-----------------RPALAADQPVPEPLTSGDRRMAELTFLGTASSQPSKYRNVSGCYVDLFE---AGGLLVDCGEDAMGQMKRRFGV**

**MpuTRZ3 (576) LEPEAKPAEAK----------------PKPP-----------------------TGAELPTPRALRGLREGDVELSFLGTGSSAPAKYRNVSGIFLDVPS---RGCMFLDAGEGTFGQLNRLYG-**

**OluTRZ3 (455) ELPEP---------------------------------------------------------EYLRKLKPGDVELVFLGTGSAVPAKYRNVSGFSMQFGGEGYRGNIMLDTGEGSLAQMIRRFG-**

**PpaTRZ4 (525) YCEEPWLMESTPVTRTNHAGTTAAPSSDIVELHGENHESEVQETVAGSKSLTLNKISLEDIPPCLQGITREEMEIVFLGTGSSQPSKYRNVSAIYMHLFE---RGGIILDCGEGSYAQLRRRY--**

**SmoTRZ3 (435) LVEEPWFDKG-----------------------------------ADSRSLSS-SSLTKEIPDTLKAIGRKEVELIFLGTGSSQPSTYRNVSAIYVHLFA---NGGMLLDCGEGTYGQLLRRYR-**

**SmoTRZ2 (433) ---------------------------------------------------------LTTTAPACISSKADDLEIVFLGTGSMHPSKHRNVSAIYLHLFE---RGGMLLDCGEGTYGQLKRRYG-**

**AlyTRZ3 (508) IIEKLWLTESN----------------TVLP-------------------------------SCLENIRRDDMEIVILGTGSSQPSKYRNVSAIYIDLFS---RGSLLLDCGEGTLGQLKRRYG-**

**AthTRZ3 (513) IIEKLWLSECN----------------TVLP-------------------------------NCLEKIRRDDMEIVILGTGSSQPSKYRNVSAIFIDLFS---RGSLLLDCGEGTLGQLKRRYG-**

**AlyTRZ4 (557) MIEEPWLGES------------------TVP-------------------------------SCLENIRRDDMEIVLLGTGSSQPSKYRNVTAIYVDLFS---RGSILLDCGEGTLGQLKRRYG-**

**AthTRZ4 (551) MIEEPWLGES------------------TVP-------------------------------SCLENIRRDDMEIVLLGTGSSQPSKYRNVTAIYIDLFS---RGSILLDCGEGTLGQLKRRYG-**

**MguTRZ3 (595) ITEEPWLSEN------------------TLP-------------------------------PCLENLTRKDMEIVLLGTGSSQPSKYRNVSSIFIDLFS---KGSLLLDCGEGTLGQLKRRFG-**

**GmaTRZ3 (531) MIEEPWLCAN-----------------GIP--------------------------------ACLENIRRDDLEIVLLGTGSSQPSKYRNVSSIYINLFS---RGGLLLDCGEGTLGQLKRRYG-**

**GmaTRZ4 (539) MNEEPWLCAN-----------------GIP--------------------------------ACLENIRRDDLEIVLLGTGSSQPSKYRNVSSIYINLFS---RGGLLLDCGEGTLGQLKRRYG-**

**MtrTRZ3 (548) VIEEPWLSED-----------------GITP-------------------------------ACLENIRRDDLEIVLLGTGSSQPSKYRNVTSIYINLFS---KGGLLLDCGEGTLGQLKRRYG-**

**CsaTRZ3 (581) MVEEPWLDEN-----------------KVP--------------------------------SCLENIRRDDLEIVLLGTGSSQPSKYRNVSSIYINLFS---KGSMLLDCGEGTLGQLKRRYG-**

**CclTRZ3 (546) MIEESWLDEN-----------------RLP--------------------------------NCLDNVRRDDLEIVLLGTGSSQPSKYRNVSSIYVNLFS---KGSLLLDCGEGTLGQLKRRYG-**

**CsiTRZ3 (547) MIEESWLDEN-----------------RLP--------------------------------NCLDNVRRDDLEIVLLGTGSSQPSKYRNVSSIYVNLFS---KGSLLLDCGEGTLGQLKRRYG-**

**CpaTRZ3 (542) MIEEPWLSEN-----------------SAP--------------------------------NCLENISRDDLEIVLLGTGSSQPSKYRNVSSIYLNLFS---KGSLLLDCGEGTLGQLKRRYG-**

**MesTRZ3 (550) MIEEPWLVDN-----------------ALP--------------------------------SCLENIRRDDLEIVLLGTGSSQPSKYRNVSSVYINLFS---KGGLLLDCGEGTLGQLKRRYG-**

**PtrTRZ3 (545) FTEEPWLEEN-----------------TLP--------------------------------SCLENIRRDDLEVVLLGTGSSQPSKYRNVTSIYINLFS---KGSLLLDCGEGTLGQLKRRYG-**

**EgrTRZ3 (573) --EEPWLSEN-----------------SLP--------------------------------NCLENIRRDDLEIVLLGTGSSQPSKYRNVSSIYINLFA---KGGMLLDCGEGTLGQMKRRFG-**

**PpeTRZ3 (580) IVEEPWFDEN-----------------TLP--------------------------------SCLENIRRDDLEIVLLGTGSSQPSKYRNVSSIHINLFS---KGGLLLDCGEGTLGQLKRRYG-**

**VviTRZ3 (564) MIEEPWLNWN-----------------TLP--------------------------------GCLENITREDMEIVLLGTGSSQPSKYRNVTSIYINLFS---KGSLLLDCGEGTLGQLKRRFS-**

**AcoTRZ3 (576) MTEEPWLNGDY----------------ASIPNINTGDNGTSTSGKVISGLSITEFERDNALPSCLENITKEDMEVVLLGTGSSQPSKYRNVSSIYVNLFS---KGSLLLDCGEGSLGQLKRRFG-**

**AcoTRZ4 (488) VVKESLINGNA----------------SLQNMKLGVQNVCDFVERILS-----EDKLECPLPSSLEKISREDMEIVLLGTGSSQPSTFRNVSSIFVNLFS---KGGLLLDCGEGTLGQLKRRYG-**

**MesTRZ4 (542) ISEKPLLDGN------------------TVP-------------------------------SFLQNIRRDDLEIVLLGTGSSQPSKYRNVSSIYINLFS---KGSLLLDCGEGTLAQLKRRYG-**

**BdiTRZ3 (535) MVEEPWINQIS----------------DKLDDGTPFQEI----------VWRKHPRDNQETPCCVEDATREDMEITFLGTGSSQPSKYRNVSSIYIKLFA---RGGILLDCGEGTLGQLKRRFG-**

**SbiTRZ4 (536) MVEEPWINQKS----------------DKLDDGASFQEI----------VWGKYPRGNQETPCCVEDATREDMEITFLGTGSSQPSKYRNVSSIYINLFA---RGGILLDCGEGTLGQLKRRFG-**

**ZmaTRZ3 (534) MGEEQWINKKS----------------DKVDDGTLFQEI----------VLRKHPRDNQEIPCCVEDATREDMEITFLGTGSSQPSKYRNVSSIYINLFE---RGGILLDCGEGTLGQLKRRFG-**

**SitTRZ5 (530) MVEEPWINQKS----------------DKLDDGT---------------IFQKHPRDNQEIPCCVEDATREDMEITFLGTGSSQPSKYRNVSSIYINLFA---RGGILLDCGEGTLGQLKRRFG-**

**OsaTRZ3 (557) MVEEPWITENSSVCNFVDENSNS----GKLQDGTPLRAS----------GWRKHPKDTPDIPCCVENATREDMEITFLGTGSSQPSKYRNVSSIYINLFT---QGGILLDCGEGTLGQLKRRFG-**

**Motif II**

**CreTRZ2 (624) AGAAARL-EELALVWVSHMHADHHGGLYRLLEWRARRGCP-----PL--LVVGPQRLFEVLVKYSSVVPIQFTFVPNLALSNASA----------------------------------------**

**VcaTRZ2 (627) QEAERRLGEQLAAVWVSHMHADHHGGLYRLLEWRARRGAP-----PL--LIIGPTSLFEILLRYSAVVPMQFLFCRNVSLWGG------------------------------------------**

**MpuTRZ3 (658) AAGADARLRRLKMIWISHVHADHHVGVPTLLAERRARMIASGIENPPPLVVVGPPALRRYLLAYEQVQPLCYHFVACHEVREDRWAAAAADAR--------------------------------**

**OluTRZ3 (522) ASAVDEKLREMRMVWISHIHADHHVGLPRILTARAEIFRRDGVE-PPIIPVVGPRALRRFLDYYEDLERLHCDFIDLSETTQDKWS---------------------------------------**

**PpaTRZ4 (645) GSQTDDVLAGLKLVWISHIHADHHTGLVRILAVRKAILEAR-GA-FSPVLVIGPKQLKRFLDAYGQLEDLGMEFVDCSQTTYDADDIAEVEQAASRSDTEDIVETPNSSRSPEKEIATASSRIGL**

**SmoTRZ3 (520) VFRTDSVVAGLRLIWISHIHADHHGGLSRILSVRRQLLSKS-GN-VEPLLVVGPKLLKRVLEAYDMVEDLGVEFLDCSQTTLEASDIAAGGQPK-----------------------------GF**

**SmoTRZ2 (497) IQGADSVLANLKCIWISHIHADHQSGLTRILTARKALLQAQ-GR-VQPILVIGPMFLRRYLTAYERLETLAMDFLDCSQTTIAAGSYNSKFRDT-------------------------------**

**AlyTRZ3 (582) LNGADEAVRKLRCIWISHIHADHHTGLARILALRSKLLKG--VT-HEPVIVVGPRPLKRFLDAYQRLEDLDMEFLDCRSTTATSWASLESGGG----------------------------DAEG**

**AthTRZ3 (587) LDGADEAVRKLRCIWISHIHADHHTGLARILALRSKLLKG--VT-HEPVIVVGPRPLKRFLDAYQRLEDLDMEFLDCRSTTATSWASLESGG-----------------------------EAEG**

**AlyTRZ4 (629) LEGADEAVRNLRCIWISHIHADHHTGLARILARRRELLKG--VA-HEPAIVVGPRPLKNFLDAYQRLEDLDMEFLDCRNTTTTSWASQETTRPEKNTNS---------------------GNAEG**

**AthTRZ4 (623) LEGADEAVRNLRCIWISHIHADHHTGLARILARRRELLKG--LA-HEPAIVVGPRSLKNFLDAYQRLEDLDMEFLDCRNTTTTSWASVETSRPEKNTSS---------------------GNAEG**

**MguTRZ3 (667) VQGADEAVRKLRCIWISHIHADHHTGLARILALRRDLLKG--TS-HEPVIVVGPRQLKRFLAAYQRLEDLDMQFLDCSQTTEASIESNQGDNNNNNNNNKN-------------------GNADS**

**GmaTRZ3 (603) VTGADDAVRTLRCIWISHIHADHHTGLARILALRRDLLRG--VP-HEPLLVVGPRQLKRYLDAYQRLEDLDMLFLDCKHTTAASLEAFEDDFPGNSVNSRNLN---------NNNGDLIASKVDS**

**GmaTRZ4 (611) VTGADDAVRTLRCIWISHIHADHHTGLARILALRRDLLRG--VP-HEPVLVVGPRQLKRYLDAYQRLEDLDMLFLDCKHTTAASLEAFEDDFPGNSVNSQNLK---------NNNGDLIASKVNS**

**MtrTRZ3 (621) VSGADDVVRSLSCIWISHIHADHHTGLTRILALRRDLLKG--VP-HEPVLVVGPRMLKRYLDAYHRLEDLDMLFLDCKHTFEASLADFENDLQ-ETVNSLDLN---------NNNAEINASKVDS**

**CsaTRZ3 (653) VEGADAAVRSLRCIWISHIHADHHTGLARILALRRDLLRE--VP-HEPVLVIGPRQLRRYLNAYQRLEDLDMQFLDCKDTTEASLEAFQKLAS-DIDNSPSESPISSTNENSTLIDGTIGRKTES**

**CclTRZ3 (618) VEGADSAVGKLRCIWISHIHADHHAGLARILALRRDLLKG--VP-HEPLLVVGPGPLKRYLDAYERLEDLDMQFLHCRYTREASWNDFEGNGEPVKNLSTPGSP---------FSTEGLINKTEA**

**CsiTRZ3 (619) VEGADSAVRKLRCIWISHIHADHHAGLARILALRRDLLKG--VP-HEPLLVVGPGPLKRYLDAYERLEDLDMQFLHCRYTREASWNDFEGNGEPVKNLSTPGSP---------FSTEGLINKTEA**

**CpaTRZ3 (614) VEGADNAVKNLRCIWISHIHADHHTGLARILALRRDLLQG--VP-HEPLLVVGPRQLKRFLDAYQRLEDLDMLFLDCKYTTEVSWNSFESDLESKKSYSSPGSVID-----FEDTNNKNAKNTQG**

**MesTRZ3 (622) LEGADIVVRNLKCIWISHIHADHHTGLSRILALRRDLLKG--MA-HEPLVVVGPRQLKRFLDAYQKLEDLDMQFLDCRSTNLASWEAYEGNSE-HKDHSATGNP------NNLEDVSTPTVSTEA**

**PtrTRZ3 (617) VEGADNAVRNLRGIWISHIHADHHTGLARILALRRDLLKG--MT-HEPVLVVGPWQLKKFLDAYQRLEDLDMQFIDCRSTTEASWVAFEDDSESKKDDLSLGSP------NNFAEMKNPTLNTES**

**EgrTRZ3 (643) VGGADDAVKGLRCIWISHIHADHHSGLARILAQRRDLLKG--VP-HEPLLVIGPRQLKRFLDAYQRLEDLDMQFLDCRNTTEASLNFFEEASELSIDQSSPESPNSH-DVLRKVTNNSSTNVPDS**

**PpeTRZ3 (652) VEGADNAVRGLRCIWISHIHADHHTGLARILTLRRDLLKG--VP-HEPLLVVGPRKLKFFLDAYQRLEDLDMQFLDCKHTTEASLHAFEGVTETNKDHSFLGSPASFEDLIDKNTDRQVAQKVDS**

**VviTRZ3 (636) VEGADNAVRGLRCIWISHIHADHHAGLARILTLRRDLLKG--VP-HEPLLVIGPRQLKRYLDAYQKLEDLDMQFLDCRHTTEVSLNAFECSFETNKEHSSP-----------E--VELMNQNIDS**

**AcoTRZ3 (681) VKGADDAIRSLKCIWISHIHADHHTGLARILSLRRELLKG--VS-HEPLLVIGPRQLKRFLDAYQRLEDLDMQFVDCRHTTEASLASFESSIELRGNHSPPIDV----------HQSLVAQNSET**

**AcoTRZ4 (588) IRGADDVVRSLRCIWISHIHADHHTGLTRILALRCELLRG--VP-HEPLLVIGPIQLEKFLEAYQKLEDLDMQFLDCRRTTEASMTDFES-----------------------------------**

**MesTRZ4 (614) MEGAENAVRNLRCIWISHIHADHHAGIARILALRRDLLKG--VP-HERLLVIGPMQLELFLDAYQRLEDLDMQFLDCRSTMDTSWNALECDAESKSNQFFAGSSTN-----SEDLNDKNKNHMGS**

**BdiTRZ3 (630) VNGADEAVKGLRCIWISHIHADHHTGLARILALRSKLLKG--MP-HKPLLVIGPRPLERFLNAYSTLEDLDMQFLDCRHTLKSSVEAFLSENDSESAIP----------------------QLEN**

**SbiTRZ4 (631) VNGADEAVKGLRCIWISHIHADHHTGLARILALRSKLLRG--MP-HKPLLVIGPRPLERFLNAYSTLEDLDMQFLDCRHTLKSSVEAFLSENDSEPAIP----------------------QLAN**

**ZmaTRZ3 (629) VNDADEAVKGLRCIWISHIHADHHTGLARILALRSKLLKG--VP-HKPLLVIGPRPLERFLNAYSTLEHLDMQFLDCRHTLKSSVEAFLSENDTDPATP----------------------QLET**

**SitTRZ5 (620) VNGADEAVKNLRCIWISHIHADHHTGLARVLALRSKLLKG--MP-HKPLLVIGPRPLERFLNAYSTLEDLDMQFLDCRHTLKPSVEAFLSENVTGSATP----------------------QLEN**

**OsaTRZ3 (664) VSGADDAVKSLKCIWISHIHADHHTGVARVLALRSKLLKG--VP-HKPLLVIGPRPLERFLNAYSTLEDLDMQFLDCRQTLKPSIEAFLSDNATESATS----------------------QLGS**

**Motif III Motif IV HEAT**

**CreTRZ2 (701) ---------D------------RQLPPAVQRVLESTLARLGLGWRRLPPRGFRPFYVHHIHDAHGLRLEGEA----------GWSIVFSGDTRPCNETIAAARGATLLVHEATFEESMVGEAKAK**

**VcaTRZ2 (703) ----------------------RVGARGMSVMPKAVAAAYEAAKERLGLAALAPFPVEHIKDAHGLVLEGRA----------GWRLVFSGDTRPCRQTVEAARGATLLVHEATFEESMEGEARAK**

**MpuTRZ3 (751) RFPSLGFYEER------------------------SLNLVRAACEEMELARVVSAPVVHCAQSYAVSVEAAD-------VVPGWKLVYSGDTRPCESLTRLATDATVLVHEATFENDMDEDAIKK**

**OluTRZ3 (607) -----------------------------SETMSPQVARLRTALMGSDLDEIVAVPVHHCAHAYGAKLVGKR----------GWTMVYSGDTRPCPSLVAAARDATLLVHEATFENGMEEEAVKK**

**PpaTRZ4 (768) VRRSYSNSKTSPKKGQMRNYWLLPGANLMEGIDWSGRDKLRKTLSSLKLASLTSVPVVHCPHAFGVVLESEARTQPDGKRRKGWKIVYSGDTRPCQALVDASEGATVLIHEATFDDSMPEEAYAK**

**SmoTRZ3 (614) VSSIVSGSQEG-RSG--LMQWVQRGYHLRNGLDEAGRSKLQDTLQALGLSSLVSVPVIHCPHAFGVVLEAQNKADTS---KAGWKLAYSGDTRPCKAFIEASYGATVFIHEATFEDGMSEEAVSK**

**SmoTRZ2 (589) LSLLCGGKRKHIDTG-------ETGYDLATGLDATGRKKLDQVLQELGLKSLLSIPVIHCAHSFGIVLESAQ---------SGWKFAFSGDTRPCDAFVEAAKGATIFVHEATFDDGLLAEALEK**

**AlyTRZ3 (676) SLFSDGSPMQSVFKR--------SDIPTDNSSVLLCLKNLKKVLSEIGLDDLISFPVVHCPQAYGVVIKAAERVNSVGERILGWKMVYSGDTRPCPEIVEASRDATVLIHEATFEDALIEEALAK**

**AthTRZ3 (680) SLFTQGSPMQSVFKR--------SDISMDNSSVLLCLKNLKKVLSEIGLNDLISFPVVHCPQAYGVVIKAAERVNSVGEQILGWKMVYSGDSRPCPETVEASRDATILIHEATFEDALIEEALAK**

**AlyTRZ4 (730) SLFSKGSPMQSIYKRS-------STPLTDNSSALPFLKKLKNVLGEMGLEDLISFPVVHCPQAFGFVVKAAKRKNIAGDEIPGWKMVYSGDTRPCPEMVEASKGATVLIHEATFEDALVEEAVAK**

**AthTRZ4 (724) SLFSKGSLMQSIYKRP-------SSPLTDNSSALPFLKKLKKVLGEMGLEHLISFPVVHCPQAFGVSLKAAERKNIAGDEIPGWKMVYSGDTRPCPEMVEASKGATVLIHEATFEDALVEEAVAK**

**MguTRZ3 (770) TLFTRGVPMQSYWKR--------PISPSQVAAALPILESLKKVLNEAGLEALISFPVIHCPQAFGVAIRAADRVNAAGKNIPGWKIVYSGDTRPCPELVRASQGATVLIHEATFEDSMIDEAVAR**

**GmaTRZ3 (716) TLFARGSRMQTYFKR--------PGSPVDKDVVSPILKKFKEVIQEAGLKALISFPVVHCPQAFGVVLKAEERTNTVGKVIPGWKIVYSGDTRPCPELIEASGGATVLIHEATFEDAMVEEAIAR**

**GmaTRZ4 (724) TLFARGSLMQSYFKR--------PGSPVDKDVVSPILKKFKGVIQEAGLKALISFPVVHCPQAFGVVLKAEERTNSVGKVIPGWKIVYSGDTRPCPELIEASRGATVLIHEATFEDAMVEEAIAR**

**MtrTRZ3 (733) TLFARGSPMQSLWKR--------PGSPVDKDTVYPLLRKLKGVIQEAGLNTLISFPVVHCSQSYGVVLEAEKRINSVGKVIPGWKIVYSGDTRPCPELIKASRDATVLIHEATFEEGMVLEAIAR**

**CsaTRZ3 (774) SLFVKGSRMQSYWKG--------PSSPVDINAAVPLLKCLNEVLNEAGLEALISFPVVHCPQAYGVVLKAAERVNLDGKVIPGWKIVYSGDTRPCPKLMEASRGATLLIHEATFEDSLVDEAMAK**

**CclTRZ3 (731) NLFAKGSCMQSVWKG--------PGIPVDNNAAFPLLKNLKNVLNEAGLETLISFPVVHCPQAFGFALKAAERINSVGKVIPGWKIVYSGDTRPCPELVEASRGATVLIHEATFEDGMMEEAIAK**

**CsiTRZ3 (732) NLFAKGSCMQSVWKG--------PGIPVDNNAAFPLLKNLKNVLNEAGLETLISFPVVHCPQAFGFALKAAERINSVGKVIPGWKIVYSGDTRPCPELVEASRGATVLIHEATFEDGMMEEAIAK**

**CpaTRZ3 (731) SLFARGSPMQSIWKK--------PGSPTDNNAAFPFLKTLRRVLNEAGLESLISFPVVHCPQAYGIVLKAAERINSVGKVIPGWKVAYSGDTRPCPELVEASCGATILIHEATFEDSMVEEAVTK**

**MesTRZ3 (737) TLFARGSRMQSYWKR--------PGSPVDNAMSFPVLKSLKKVLSEAGLEALISFPVVHCPQAFGIMLKAAERINAVGKIIPGWKIVYSGDTRPCPELVEASKGATVLIHEATFEDDLVEEAVAR**

**PtrTRZ3 (733) NLFARGNRMQSYWKR--------PGSPVDNGMVFPSLKRLKEVLSEAGLEALISFPVVHCPQAFGIALKAAERINTVGKVIPGWKIVYSGDTRPCPELVEASRGATILIHEATFEDALVEEAIAR**

**EgrTRZ3 (764) TLFARGAPMQSFWKR--------PGSPPDISVAFPVLQNMKKMLKEAGLDALVSFPVVHCPQAFGVVLKASERLNSDGKLIEGWKIVYSGDTRPCPELTEASRGATVLIHEATFEDSMVDEAIAR**

**PpeTRZ3 (774) TLFAKGSRMQSYWKR--------PGSPVDNNVVFPILKSLQKVLEEAGLEALMSFPVIHCPQAFGVVLRASERLNSVGKVIPGWKIVYSGDTRPCPELTEASRGATVLIHEATFEDGMVDEAIAR**

**VviTRZ3 (745) SLFAKGSRMQSYWKR--------PGSPVDHSVAFPILKNLKKVLCEAGLEALISFPVVHCPQAFGVVLKASERINSVGKVIPGWKIVYSGDTRPCPELIEAARGATVLIHEATFEEGMVDEAIAR**

**AcoTRZ3 (793) SLFARGSRMQSYLNR--------PSSPVDIAMGLEILRNLKKVLGEAGLEALVSVPVVHCPQAYGLVLRAAERTNSAGKIIPGWKLVYSGDTRPCPELIEASRGATVLIHEATFEDGMVEEAIAR**

**AcoTRZ4 (675) -------------------------------------THLKKVLVEANLDSLVSIPVLHCPNAFGLVVKAAERINSIGKTIPGWKLVYSGDTRPCPELEKAADGATVLIHEATFEDGMEKDADAK**

**MesTRZ4 (731) TLFAGESCLQGCSKR---------MKLSMPVENDSLLRSLRNVLWGAGLEGLISFPVVHCPEAFGVVLKAAERTNAVGEIIQGWKIVYSGDTRPCSEVIEASHGATVLIHEATFEDCMVDEAVEK**

**BdiTRZ3 (730) TMFAPGSRMENYNRK--------PASPRDTT----ALANFKEVLQESGLEILYSVPVLHCPQAFGVVFRAMEKTNSAGKVIPGWKVVYSGDTRPCPALIDASRDATVLIHEATFEDSMKDEAIAR**

**SbiTRZ4 (731) TIFAPGSRMENYNRK--------PASPRDTT----ALANFKEVLQESGLEILYSVPVLHCPQAFGVVFRAMEKTNSTGKVIPGWKVVYSGDTRPCPALIDASRDATVLIHEATFEDSMKDEAIAR**

**ZmaTRZ3 (729) TMFAPGTRMENYNRK--------PASPRDTT----ALANFKEVLQESGLEILYSVPVLHCPQAFGVVLKAMEKANSTGKVIPGWKVVYSGDTRPCPGLIDASRDATVLIHEATFEDSMKDEAIAR**

**SitTRZ5 (720) TMFAPGSRMENYNRK--------PASPRDTT----ALANFKEVLLESGLEILYSVPVVHCPQAFGVVLRAMEKVNSAGKVIPGWKVVYSGDTRPCPALIDASRDATVLIHEATFEDSMKDEAIAR**

**OsaTRZ3 (764) TIFAPGSKMENYSRK--------PASPRDTT----ALTNLKDVLHESGLEVLYSVPVLHCPQAFGVVLRAKEKVSSAGKAIPGWKVVYSGDTRPCPALVDASRDATVLIHEATFEDSMKDEAIAR**

**HST Motif V**

**CreTRZ2 (795) KHSTTAEAVSVGERAGAYRIILTHFSTRYPTLPELDLAPHPRVSVAMDLQVVNLA-DLPWQPALVRPLGLLFKQLEAEKLKDDDDDE----------------------**

**VcaTRZ2 (796) RHSTTAEAVGVGEEAGVYRTVLTHFSTRYPTLPELDLSRHPRVAVAMDLMSINLA-DLPWLPRLVRPMGLLFKHLEAEKLAADDDDD----------------------**

**MpuTRZ3 (845) RHSTTRDAVRTGVEARAYRTILTHFSQRYPKIPVLDESVSERTAVAFDMMRVDFARDLPRLPSLVPAVQAVFLDPEEVFGGGGNNTAAANKKKKWGPPPRGGSKSPPRR**

**OluTRZ3 (693) RHSTTKEAVQTGIDAGAYRTILTHFSQRYPKIPIFDGTYTERTAVAFDLMSVDFA-GLSSLPKLLPAVRSLFESDDEIAREAQIDGDDDE-------------------**

**PpaTRZ4 (893) KHSLTREAIETGVSAGVYRTILTHFSQRYPKIPVFDDSYTSQTCIAFDMMSVNLA-DLPLLPSLLPALKLLFKDDMASMEEEKEEVTEEAMVL----------------**

**SmoTRZ3 (733) MHSSTHEAIQAGALARAYRTILTHFSQRYSKVPVFDDSYNDRTCVAFDLMSIDLV-DLPLLPSLIPVLKLLFKDDQAEEQAVPEAVAQ---------------------**

**SmoTRZ2 (698) NHSLTCEAVQAGAAAGAYRTILTHFSQRYPQIPVFDASYNERTCIAFDMMSVNLV-DLPLLPKLVPVMKLMFKEE----------------------------------**

**AlyTRZ3 (793) NHSTTKEAIDVGSSANAYRIVLTHFSQRYPKIPVIDESHMHNTCIAFDLMSINMA-DLQVLPKVLPYFKTLFRDEMVEDEDADGVAMEEAL------------------**

**AthTRZ3 (797) NHSTTKEAIDVGSAANVYRIVLTHFSQRYPKIPVIDESHMHNTCIAFDLMSINMA-DLHVLPKVLPYFKTLFRDEMVEDEDADDVAMDDLKEEAL--------------**

**AlyTRZ4 (848) NHSTTKEAINVGSSAGVYRTVLTHFSQRYPKIPVIDESHMHNTCIAFDMMSVNMA-DLHVLPKILPYFKTLFRNQVVEEEEEE--TDNDSLMSDKVPSFFIN-------**

**AthTRZ4 (842) NHSTTKEAIKVGSSAGVYRTVLTHFSQRYPKIPVIDESHMHNTCIAFDMMSINMA-DLHVLPKILPYFKTLFRNQVVEEEEEEEETDDDSLIRDKVPSFFIN-------**

**MguTRZ3 (887) NHSTTKEAVEVGNSAGAYRIILTHFSQRYPKIPVFEESHMHKTCVAFDMMSVNLA-DVHVLPRVVPYLKLLFRDEMIVDESEDVDLVTAIA------------------**

**GmaTRZ3 (833) NHSTTNEAIEMGQSANAYRTILTHFSQRYPKIPVFDETHMHKTCIAFDMMSVNVA-DLSVLPKALPYLKLLFRNEMMVDESDDVVEAVTSAS-----------------**

**GmaTRZ4 (841) NHSTTNEAIKMGQSANAYRTILTHFSQRYPKIPVFDETHMHKTCIAFDMMSVNVA-DLSVLPKVLPYLKLLFRNEMMVDESDDVVEAVTSAS-----------------**

**MtrTRZ3 (850) NHSTTNEAIETGEAANVYRIILTHFSQRYPKIPVINKEHMDITCIAFDLMSINIA-DLPVLPKVLPYLKLLFRNDMTVDESNDVVVTVDESDDVVDVATSAS-------**

**CsaTRZ3 (891) NHSTTSEAIDIGNSAGAYRIILTHFSQRYPKIPVVDEKHMHKTCIAFDLMSVNVA-DLSVLPKVLPYLTLLFRDEMMVDESDDVTMES---------------------**

**CclTRZ3 (848) NHSTTKEAIDVGSSAGVYRIILTHFSQRYPKIPVVDETHMHKTCIAFDLMSINLA-DLPILPKVLPYFKLLFKDEMPVDESDDVVDAVSAAS-----------------**

**CsiTRZ3 (849) NHSTTKEAIDVGSSAGVYRIILTHFSQRYPKIPVVDETHMHKTCIAFDLMSINLA-DLPILPKVLPYFKLLFKDEMPVDESDDVVDAVSAAS-----------------**

**CpaTRZ3 (848) NHSTTKEAVEVGSSAGAYRIILTHFSQRYPKIPVVDETYMHKTCIAFDMMSINIA-DLSVLPKVVPYLKLLFRNEMALDESDDVYRCYNCSFIINIY------------**

**MesTRZ3 (854) NHSTTKEAIEVGDSAGAYRIILTHFSQRYPKIPVFDETHMHKTCIAFDMMSVNVA-DLPVLPKVLPYLKLLFKNEMIVDELDDAADAVSAVS-----------------**

**PtrTRZ3 (850) NHSTTEEAIEVGNSAGAYRIILTHFSQRYPKIPVFDETHMHKTCIAFDMMSVNIA-DLPVLPRVLPYLKMLFRNEMVVDESDDVVDAASAVAN----------------**

**EgrTRZ3 (881) NHSTTEEAIQMGASAGVYRIVLTHFSQRYPKIPVFDEAHMHKTCIAFDLMSINIA-DLPMLPRVLPYLKMLFRNEMIVDESDDTGDSVSVAS-----------------**

**PpeTRZ3 (891) NHSTTKEAIEVGNSAGVFRIILTHFSQRYPKIPVFDETHMHKTCIGFDMMSINIA-DLPVLPKVLPYLKLLFRNELIIDESDEVVDAAASVAS----------------**

**VviTRZ3 (862) NHSTTNEAIEVGNSAGAYRIILTHFSQRYPKIPVFDDAHMHKTCIAFDLMSVNMA-DLPVLPKVLPYLKLLFRNEMTVDELDDVISAGLAS------------------**

**AcoTRZ3 (910) NHSTTKEAIDVGDSAGAYRIILTHFSQRYPKIPVFDETHMHKTCIGFDLMSINIA-DLPVLPKILPHIKLLFRDEMITDEVDDIVDSVL--------------------**

**AcoTRZ4 (763) NHSTTKQAIKVGDKA--YRIILSHFSQRYPKIPVFDETHMHKTCIGFDLMSINIA-DLHVLPKILPYLKLLFRDKITAPESEDMQDTLE--------------------**

**MesTRZ4 (847) NHSTTKEAIEVGDSAGAYRVILTHFSQRYPKIPALDEISMKKTCIAFDLMSVNIA-DLPMLPKILPYLKLLFRTDIAS-------------------------------**

**BdiTRZ3 (843) NHSTTKEAIEVGTSAGAYRIILTHFSQRYPKIPVIDEVDMEKTCIAFDLMSVNLA-DLPVVPKVLPHLKVLFKDELVVDEADEIQEAAVY-------------------**

**SbiTRZ4 (844) NHSTTKEAIEVGTSAGAYRIILTHFSQRYPKIPVIDEVDMEKTCIAFDLMSVNLA-DLPVVPKVLPHLKVLFKDELVVDEADELQEAAVY-------------------**

**ZmaTRZ3 (842) NHSTTKEAIEVGTSAGAYRIILTHFSQRYPKIPVIDEVDMEKTCIAFDLMSVNLA-DLPVVPKVLPHLKVLFKDELVVDEAEEVQEAAVY-------------------**

**SitTRZ5 (833) NHSTTKEAIEVGTSAGAYRIILTHFSQRYPKIPVIDEVDMEKTCIAFDLMSVNLV-DLPVLPKVLPHLKVLFKDELVVEEADEIQEAAAY-------------------**

**OsaTRZ3 (877) NHSTTKEAIAVGTSAGAYRIILTHFSQRYPKIPVFDEVDMQKTCIAFDLMSVNLA-DLPVLPKVLPHLKLLFKDEMVVDESDEIQEAVM--------------------**
